# Marine Biodiversity in Japanese Waters

**DOI:** 10.1371/journal.pone.0011836

**Published:** 2010-08-02

**Authors:** Katsunori Fujikura, Dhugal Lindsay, Hiroshi Kitazato, Shuhei Nishida, Yoshihisa Shirayama

**Affiliations:** 1 Institute of Biogeosciences, Japan Agency for Marine-Earth Science and Technology, Yokosuka, Kanagawa, Japan; 2 Atmosphere and Ocean Research Institute, University of Tokyo, Kashiwa, Chiba, Japan; 3 Seto Marine Biological Laboratory, Kyoto University, Shirahama, Wakayama, Japan; George Mason University, United States of America

## Abstract

To understand marine biodiversity in Japanese waters, we have compiled information on the marine biota in Japanese waters, including the number of described species (species richness), the history of marine biology research in Japan, the state of knowledge, the number of endemic species, the number of identified but undescribed species, the number of known introduced species, and the number of taxonomic experts and identification guides, with consideration of the general ocean environmental background, such as the physical and geological settings. A total of 33,629 species have been reported to occur in Japanese waters. The state of knowledge was extremely variable, with taxa containing many inconspicuous, smaller species tending to be less well known. The total number of identified but undescribed species was at least 121,913. The total number of described species combined with the number of identified but undescribed species reached 155,542. This is the best estimate of the total number of species in Japanese waters and indicates that more than 70% of Japan's marine biodiversity remains un-described. The number of species reported as introduced into Japanese waters was 39. This is the first attempt to estimate species richness for all marine species in Japanese waters. Although its marine biota can be considered relatively well known, at least within the Asian-Pacific region, considering the vast number of different marine environments such as coral reefs, ocean trenches, ice-bound waters, methane seeps, and hydrothermal vents, much work remains to be done. We expect global change to have a tremendous impact on marine biodiversity and ecosystems. Japan is in a particularly suitable geographic situation and has a lot of facilities for conducting marine science research. Japan has an important responsibility to contribute to our understanding of life in the oceans.

## Introduction

Understanding the biodiversity and function of marine ecosystems, and how they respond to global change and human activities, is essential to maintaining sustainable human life in harmony with nature, because humans are directly or indirectly dependent on marine life. However, the resources to identify and inventory marine biodiversity have not increased commensurately with this demand [1]. To contribute to our understanding of marine ecosystems, a global network called the Census of Marine Life (Census) was implemented in 2000 (http://www.coml.org/). The purpose of the Census is to assess and explain the diversity, distribution, and abundance of marine life. To strengthen support for marine biodiversity research at the country or regional scale, the Census formed National and Regional Implementation Committees (NRICs) in 12 countries or regions. The role of the NRICs is to identify research and data priorities for marine biodiversity. The work reported here contributes to the Japan NRICs efforts.

Japan has a rich marine species diversity because of a combination of various historical and environmental (geological and physical oceanographic) factors [2]. Traditionally, the marine biota has constituted an important food resource in Japan owing to the overpopulation of the country. According to the Food and Agriculture Organization of the United Nations statistics, Japan's average per capita consumption of fishery products was 66.9 kg per year for 2001–2003, approximately four times the world average. Many Japanese people understand the importance of the marine biota, and marine biology, including taxonomy, ecology, and physiology, is more well studied in Japan than in many other nations.

Japan is surrounded by the sea, and marine ecosystem services are expected to be affected by global climate change and human impacts on the ocean. Thus, to understand marine biodiversity in Japanese waters, we have compiled related marine biodiversity information in Japan, including species richness indicators—such as the number of described species (NDS), the number of endemic species (NES), the number of identified but undescribed species (NUS), the number of known introduced species (NIS), history of marine biology, state of knowledge, list of taxonomic experts and identification guides with consideration of the oceanic environmental background (see Tables 1 and 2 for a list of abbreviations used in this study).

**Table 1:** Terminology abbreviations used in this study.

Acronym	Word or Phrase
AUV	Autonomous Underwater Vehicle
EEZ	Exclusive economic zone
ENS	Expected number of species
HOV	Human occupied vehicle
ND	No data
NDS	Number of described species
NDSo	Number of species recorded in Japanese waters in OBIS
NES	Number of endemic species
NIS	Number of known introduced species
NUS	Number of identified but undescribed species
PES	Percentage of endemic species
PRO	Percentage of species recorded in Japanese waters in OBIS
ROV	Remotely Operated Vehicle
tNDS	Total number of described species

**Table 2:** Abbreviations for institutions and organizations.

Acronym	Word or Phrase
AMSL	Akajima Marine Science Laboratory
AIST	Advanced Industrial Science and Technology
BIK	Biological Institute on Kuroshio
BISMaL	Biological Information System for Marine Life
CoML	Census of Marine Life
GBIF	Global Biodiversity Information Facility
HJC	Hakodate Junior College
IODP	Integrated Ocean Drilling Program
ISU	Ishinomaki Senshu University
JAMSTEC	Japan Agency for Marine-Earth Science and Technology
JMA	Japan Meteorological Agency
JODC	Japan Oceanographic Data Center
JSNFRI	Japan Sea National Fisheries Research Institute
KMNH	Kitakyushu Museum of Natural History and Human History
KMPC	Kushimoto Marine Park Center
LBM	Lake Biwa Museum
NHMIC	Natural History Museum and Institute, Chiba
NIES	National Institute for Environmental Studies
NIPR	National Institute of Polar Research
NITE	National Institute of Technology and Evaluation
NMNS	National Museum of Nature and Science, Tokyo,
NRICs	National and Regional Implementation Committees
NRIFS	National Research Institute of Fisheries Science
OBIS	Ocean Biogeographic Information System
OMNH	Osaka Museum of Natural History
ORI	Ocean Research Institute, the University of Tokyo
SFL	Sugamo Foraminiferal Research Laboratory
SNF	Seikai National Fisheries Research Institute
TAT	Tokyo University of Agriculture and Technology
TNFRI	Tohoku National Fisheries Research Institute
TSM	Toyama Science Museum
TUMSAT	Tokyo University of Marine Science and Technology
UBC	University of British Columbia
YNU	Yokohama National University

### General description of Japanese waters

#### Topographical and geological characteristics

Japan is an island arc located on the western Pacific side of the Northern Hemisphere and has no common land border with any other country. The Japanese archipelago is located between approximately 20°30′ N to 45°30′ N and 123° E to 150°E, and encompasses several climatic regimes from north to south, such as the subboreal zone, cool temperate zone, middle temperate zone, warm temperate zone, subtropical zone, and tropical zone. Japan's Exclusive Economic Zone (EEZ) extends from approximately 17°N to 48° N, and from approximately 122° E to 158° E. The land area of Japan is small at 3.78×10^5^ km^2^, but the EEZ is large at 4.05×10^6^ km^2^, or approximately 11 times the area of the land, and ranks as sixth largest in the world. The maximum water depth in Japanese waters is 9,780 m in the Izu-Ogasawara (Bonin) Trench.

**Figure 1:**
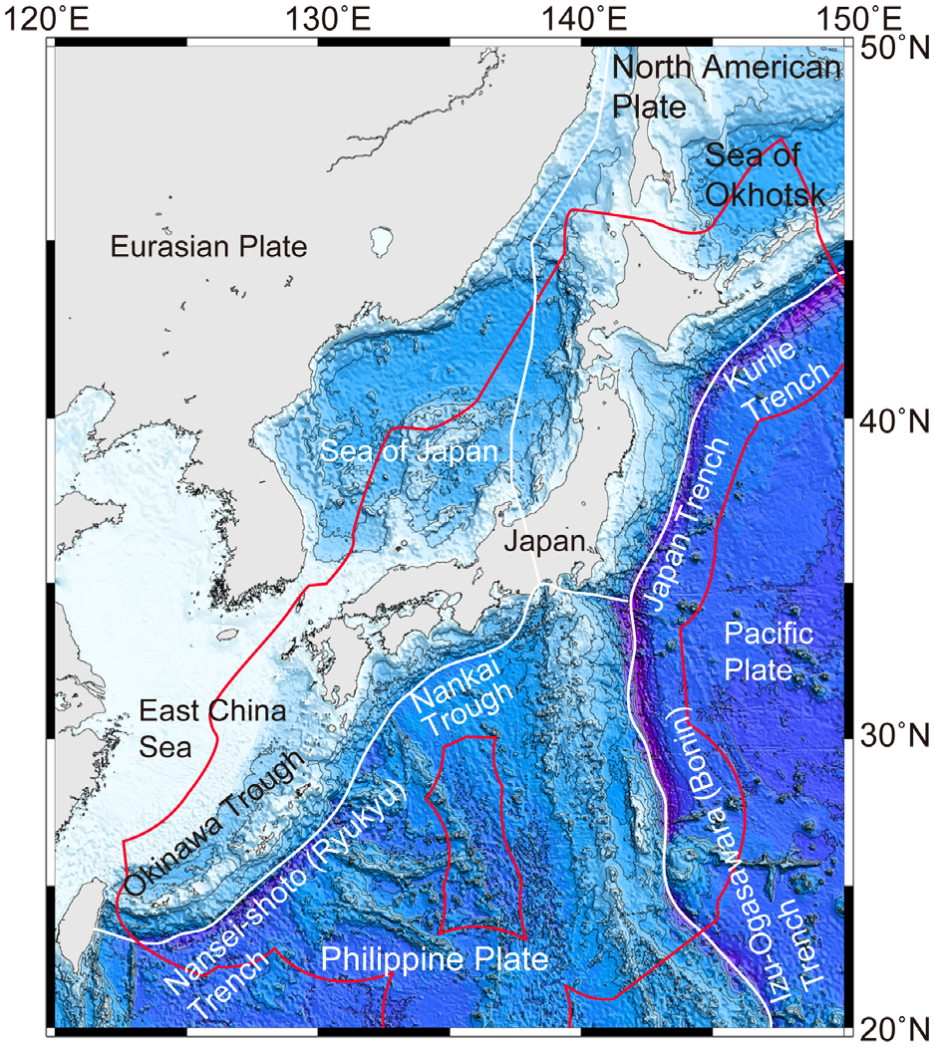
Ocean bottom topography around Japan. White and red lines indicate plate boundaries and Japan's Exclusive Economic Zone (EEZ), respectively.

**Figure 2:**
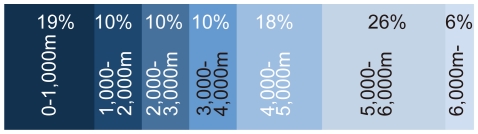
Areal ratio of each 1,000 m depth zone in Japan's Exclusive Economic Zone (EEZ). Modified from http://www.sof.or.jp/jp/news/101-150/123_3.php#1.

Japan is a nation composed of numerous islands. Hokkaido, Honshu, Shikoku and Kyushu islands form a line from north to south. There are some other groups of islands including the Chishima (Kurile) Islands off the northeast of Hokkaido, the Izu-Ogasawara (Bonin) Island chain stretching south of Honshu, and the Ryukyu Islands stretching south of Kyushu. The Japanese archipelago is situated between the North Pacific Ocean and several marginal seas, such as the Sea of Okhotsk to the north, the Sea of Japan to the west, and the East China Sea to the southwest (Figure 1). The length of the coastline is approximately 30,000 km. Sea floor depth within Japan's EEZ expressed as percentages of the total seafloor area claimed as territory by Japan is shown in Figure 2.

Topographic and geological characteristics of coastal areas and the deeper seafloor have been reported in many studies [3], [4]. There are varied topographies such as bays, beaches, inland seas, mud flats, and rocky shores along the coastlines. Land reclamation areas also are common in and around city areas. The major bays are Uchiura Bay in Hokkaido, Ise Bay, Mikawa Bay, Mutsu Bay, Sagami Bay, Suruga Bay, and Tokyo Bay on the Pacific side of Honshu, Toyama Bay and Wakasa Bay on the Sea of Japan side of Honshu, Tosa Bay in Shikoku Island, and Ariake Bay and Kagoshima Bay in Kyushu Island. Mud flats larger than one hectare in area number approximately 30, of which the largest is in Ariake Bay. The most distinctive inland sea is the Seto Inland Sea between Honshu, Shikoku, and Kyushu. This Inland Sea has an area of approximately 20,000 km^2^ and contains 720 small islands.

Four tectonic plates, namely, the Eurasian, North American, Pacific, and Philippine plates, converge in Japanese territory (Figure 1). The Pacific Plate is moving from the East Pacific Rise. A part of this plate subducts beneath the North American Plate in the Japan and Kurile trenches. Another part of this plate subducts beneath the Philippine Plate in the Izu-Ogasawara (Bonin) Trench. The northern part of the Philippine Plate subducts beneath the North American Plate. The northwestern part of the Philippine Plate subducts beneath the Eurasian Plate in the Nankai Trough and the Nansei-shoto (Ryukyu) Trench. In these plate subduction areas, island arc-trench systems are well developed. Usually, these systems are composed of active volcanoes and trenches. Sagami and Suruga troughs also belong to this system. Many submarine volcanoes are situated in the Okinawa Trough and on the west side of the Izu-Ogasawara (Bonin) Trench, namely, the Shichito-Iwojima Ridge. On the whole, sea bottom topography in Japanese waters is characterized by depression forms, such as trenches and troughs.

#### Physical and chemical characteristics

**Figure 3:**
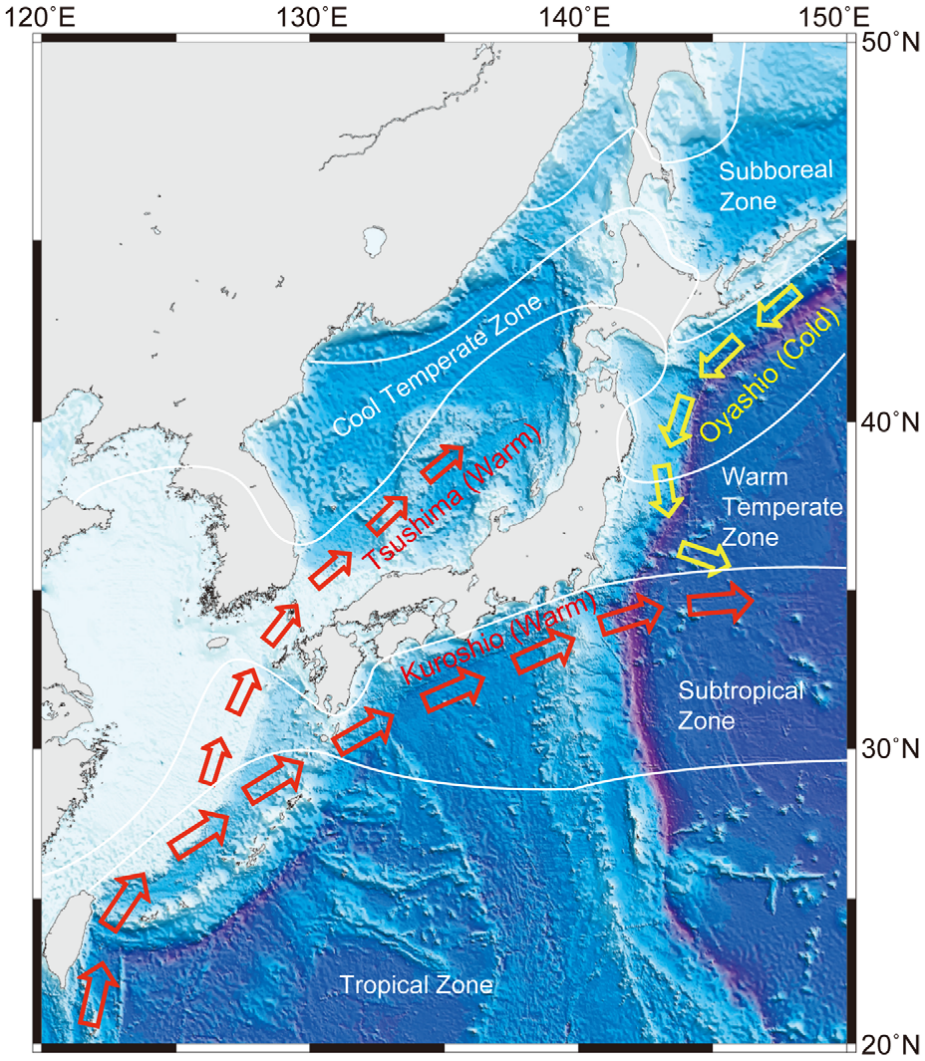
Schematic diagram of surface currents and climate regimes around Japan. Red and yellow arrows indicate warm (Kuroshio and Tsushima) and cold (Oyashio) currents, respectively.

The Kuroshio and Tsushima Currents are the major warm currents in Japanese waters, and the Oyashio Current is the major cold current (Figure 3). The Kuroshio is the largest current in the Pacific [5]. This current begins in the East China Sea and runs along the Pacific coast of Japan. The current is about 200 km wide and its influence can be recognized to as deep as 700 m. The speed in the center of the current axis is 150–250 cm sec^−1^. Transport volume is estimated at 5×10^7^ ton sec^−1^. The Tsushima Current splits from the Kuroshio Current and flows from off Kyushu into the Sea of Japan. The Oyashio Current flows southward through Japanese waters from off Hokkaido along the Pacific coast. The speed of this current is 20 cm sec^−1^ and the transportation ability of the Oyashio Current is smaller than that of the Kuroshio Current.

**Figure 4:**
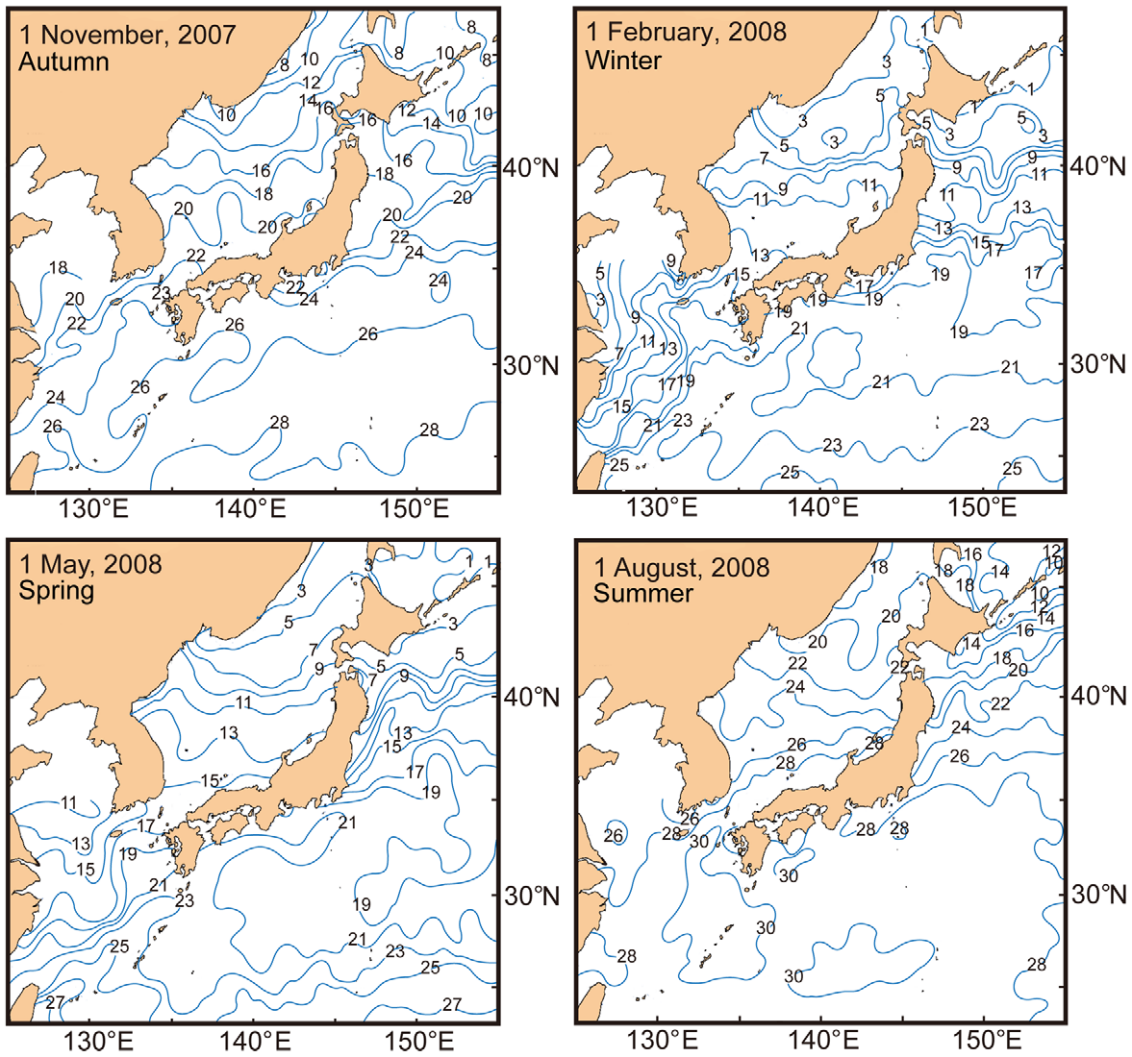
Sea surface temperature maps in each season around Japan.

**Figure 5:**
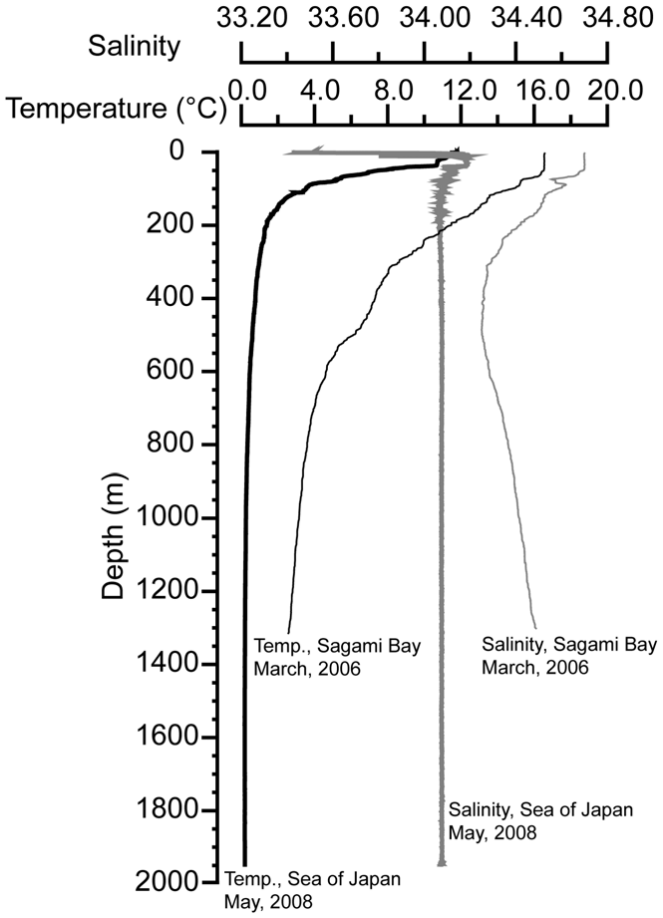
Vertical structure of temperature and salinity between Sagami Bay on the Pacific side and the Sea of Japan.

Generally, the distribution of sea surface water temperature in Japan follows the seasons and is characterized by spring, summer, autumn, and winter patterns. Figure 4 shows the sea surface temperature patterns for each season; summer is warmest and winter is coldest. The vertical temperature profile in the Sea of Japan differs sharply from that on the Pacific side (Figure 5). Temperatures in the Sea of Japan are much lower than in the Pacific. Climate regimes in Japanese waters are classified into six categories between the subboreal and tropical zones (Figure 3). The northernmost regions, such as the Sea of Okhotsk and the Pacific east of Hokkaido belong to the subboreal zone, while the southernmost areas such as the Ryukyu and Izu-Ogasawara (Bonin) island regions belong to the tropical zone. On a large scale, biogeographically, Japan belongs to the Indo-western Pacific regime.

Various ecosystems in Japanese waters are associated with each type of environment. For example, unique biological communities occur above and below drift ice on the sea surface in coastal areas in the Sea of Okhotsk off northern Hokkaido in winter. Contrastingly, coral reefs are common in the Ryukyu and Izu-Ogasawara (Bonin) island areas. Deep-sea organisms are found in bathyal, abyssal, and hadal zones such as in trenches and troughs and in the water column above them. Chemosynthesis-based communities, including hydrothermal vent and methane seep communities, are distributed along plate convergence areas because of the accompanying tectonic activity [6], [7]. Many seeps have been found in the Japan Trench, Nankai Trough, Ryukyu Trench, Sagami and Suruga bays, and the Sea of Japan. Several vent communities have been found in the Izu-Ogasawara (Bonin) Island area and in the Okinawa Trough.

#### Brief history of research in Japan

From 1616 to 1858, Japan had a foreign relations policy prohibiting the entry of foreigners into Japan proper. During this period, biological inventories were produced in the form of species lists for use in natural medicines and seasonal keywords (kigo) for Haiku poetry. However, the Dutch, who at the time were the only nationality with permission to trade with Japan, brought many marine organisms back to Holland, and these were then used by European researchers for the production of marine biological monographs. After 1858, several scientists were invited from Germany and the United States to lecture on natural history at Japanese universities. During this time, they conducted advanced investigations of the marine fauna of Japan. Before World War II, several museums and institutes were established and the foundations for marine biological research were laid (Table S1).

The fauna of Sagami Bay and the ocean off the Boso Peninsula were investigated in 1875 as part of a pioneering cruise by the HMS *Challenger* (1872–76). Scientists of the *Challenger* purchased many marine organisms at the fish market for their biological samples. Also, scientists from the United States investigated Sagami and Suruga bays using the HMS *Albatross* in 1906. The first human-occupied vehicle (HOV) designed specifically for studies on marine biology was named the Nishimura-shiki Mame Sensui-tei ichi-go and was developed in 1929 in Japan. This vehicle had sampling gear, a diesel engine, lights, two view ports, and an underwater telephone system. After 1955, large-scale investigations on marine fauna have been conducted using many different research vessels in collaboration with international projects. The former Emperor Hirohito actively studied the taxonomy of marine animals, and he and his colleagues published several monographs on the Arthropoda, Ascidia, Cnidaria, Echinodermata, Mollusca, and Porifera [8]–[18].

## Methods

### Species richness estimation

Three species richness indices—including the number of described species (NDS), the number of endemic species (NES), and the number of identified but undescribed species (NUS), as well as the number of known introduced species (NIS)—were estimated for each taxonomic order of organisms occurring in Japanese waters. In cases where it was impossible to classify species to order, these indicators were estimated at the superorder, infraorder, suborder, or family level. Additionally, the number of taxonomic experts and identification guides such as monographs, illustrated books, related scientific papers, or URLs for identification of species were identified and counted. In cases where many experts exist for each taxon, only two experts' names were shown. Identification guides were chosen for their ability to satisfy the basic requirements of students and scientists studying and working on marine biology [1].

For each taxon, an attempt was made to estimate the status of knowledge under a five-stage classification system based on the following definitions:

5: Very well known. Satisfies all of the following requirements: (1) more than 80% or more than 100 species occurring in Japanese waters have been described in the scientific literature, (2) identification guides including monographs, illustrated books, or related scientific papers have been published within the last 20 years, and (3) more than one taxonomic expert exists in Japan.

4: Well known. (1) More than 70% or more than 10 species occurring in Japanese waters have been described in the scientific literature, (2) identification guides including monographs, illustrated books or related scientific papers have been published, and (3) one or more taxonomic experts exist in Japan.

3: Poorly known. (1) More than 50% or fewer than 10 species occurring in Japanese waters have been described in the scientific literature, (2) at least one publication aiding identification has been published in the past, and (3) no taxonomic experts active in Japan.

2: Very poorly known. Falls under at least one of the following categories: (1) less than 50% or only a few species occurring in Japanese waters have been described or, 2) no taxonomic expert and or identification guide exists anywhere in the world.

1: Unknown. Falls under at least one of the following categories: (1) no described species have been identified from Japanese waters or, (2) no published information exists.

Many experts on the taxonomy or ecology of marine organisms collaborated in the gathering of this species richness data (Table S2).

The total number of described species (tNDS) was calculated by combining the NDS for all taxa. Also, the total combined number of both described and undescribed species in each phylum or division was calculated by combining the NDS with the NUS in all orders, superorders, infraorders, suborders, or families within the phylum or division. The NUS values were estimated based on the contributor's own samples or according to their experience and knowledge. We also calculated the expected number of species (ENS) by combining the NDS and the NUS.

Endemic species were defined as those that have only been reported from Japanese waters. The percentages of NES versus NDS were calculated as the percentage of endemic species (PES) according to the following equation: PES = (NES/NDS)×100. Known introduced species were defined as those that have been introduced into Japanese waters from outside their native distributional range by human activity. We not only estimated the NIS but also recorded the species names, presumed primary mechanism of transport to Japan, and the presumed origin of the introduced species.

Unfortunately, Japan does not yet have a national marine species inventory for all marine organisms that occur in Japanese waters. Thus, where there were no active experts for a taxon, indicators were estimated using published scientific papers, or databases such as the Japanese Biota Species Number Survey (http://research2.kahaku.go.jp/ujssb/search) and others. Marine biological studies have been carried out not only in the tidal zones but also in the open ocean and in deep-sea regions using an array of research vessels. Active ocean research vessels larger than 500 tons are shown in Table 3.

**Table 3:** Ocean research vessels (more than 500 gross tons) for marine biology in Japan.

Name of research vessel	Gross tonnage	Institution/Affiliation^1^	Main mission
*Bosei-maru*	2,174	Tokai University	Multi-purpose missions
*Chikyu*	57,087	JAMSTEC	Drilling
*Hakuho-maru*	3,991	JAMSTEC	Multi-purpose missions
*Hokko-maru*	568	NRIFS	Fisheries science
*Kairei*	4,628	JAMSTEC	Support of remotely operated vehicle
*Kaiyo*	3,350	JAMSTEC	Multi-purpose missions
*Kaiyo-maru*	2,942	NRIFS	Fisheries science
*Keifu-maru*	1,882	JMA	Oceanography
*Keiten-maru*	860	Kagoshima University	Fisheries science, Oceanography
*Koyo-maru*	2,703	NRIFS	Fisheries science, Oceanography
*Mirai*	8,687	JAMSTEC	Multi-purpose missions
*Nagasaki-maru*	842	Nagasaki University	Fisheries science, Oceanography
*Natsushima*	1,739	JAMSTEC	Support of remotely operated vehicle
*Oshoro-maru*	1,792	Hokkaido University	Fisheries science, Oceanography
*Ryofu-maru*	1,380	JMA	Oceanography
*Shinyo-maru*	649	TUMSAT	Fisheries science, Oceanography
*Shirase*	12,500	NIPR	Antarctic Expedition
*Shoyo-maru*	2,494	NRIFS	Fisheries science
*Shunyo-maru*	1,228	NRIFS	Fisheries science
*Soyo-maru*	1,234	NRIFS	Fisheries science
*Tansei-maru*	610	JAMSTEC	Multi-purpose missions
*Tenyo-maru*	1,020	NRIFS	Fisheries science
*Umitaka-maru*	1,886	TUMSAT	Fisheries science, Oceanography
*Wakataka-maru*	692	NRIFS	Fisheries science
*Yoko-maru*	608	NRIFS	Fisheries science
*Yokosuka*	4,439	JAMSTEC	Support of human occupied vehicle

1 Each abbreviation is shown in Table 2.

### Comparisons between NDS and the number of species recorded from Japanese waters in OBIS

The Ocean Biogeographic Information System (OBIS: http://www.iobis.org/), the Census of Marine Life's main repository for biogeographical information, is a useful database bringing together an array of information on marine species occurrence and distribution data throughout the world ocean. We referred to the number of species recorded in Japanese waters in OBIS (NDSo) for each taxon, using the advanced search function of OBIS on 6 June 2009. The search was done within a polygon, the boundaries of which corresponded to Japan's EEZ, as claimed by Japan on the same date. The percentages of the NDS versus the NDSo were calculated as the percentage of species recorded in Japanese waters in OBIS (PRO) according to the following equation: PRO = (NDSo/NDS)×100.

## Results

### Species richness in Japanese waters

**Figure 6:**
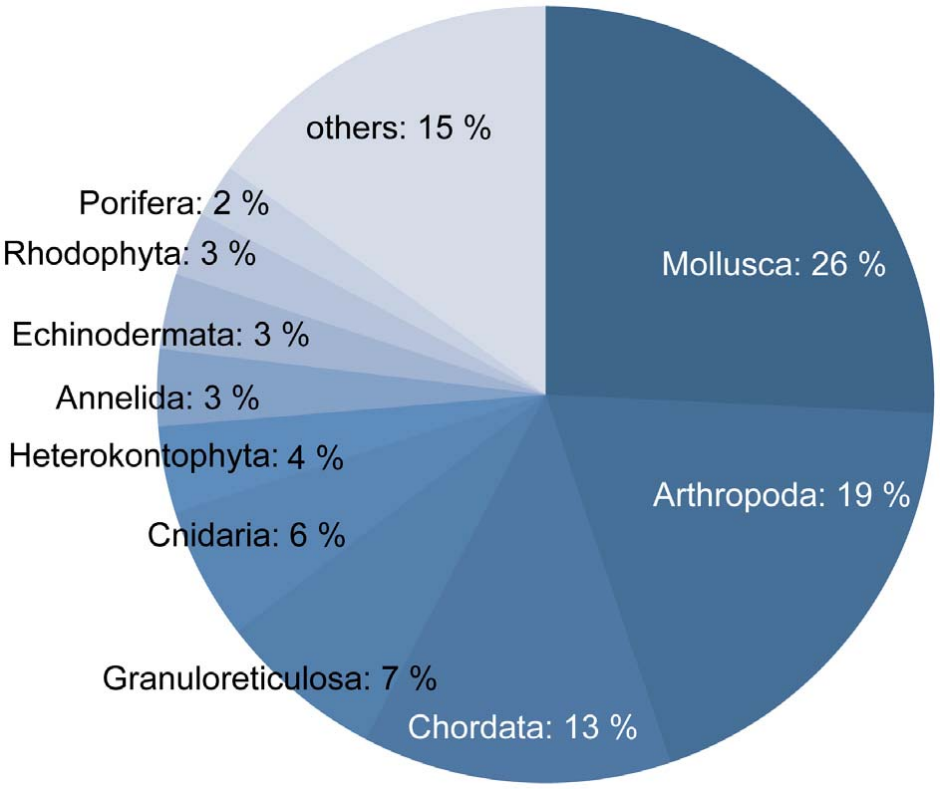
Percent ratio of the number of described species (NDS) in respective phyla. The ratio means NDS versus the total number of described species (tNDS) in all phyla ranked from top to 10th.

Summarized data concerning species richness including the NDS and NIS, and information on state of knowledge estimates, taxonomic experts, and identification guides were compiled in Table 4. More detailed data on species richness in each lower taxa (order or family levels) including the NDS, NES, NUS, ENS, NIS, and information on taxonomic experts, identification guides, and state of knowledge estimates for each taxon are shown in Table S3. The tNDS in Japanese waters reached 33,629. Among 79 phyla or divisions, 66 phyla or divisions contained more than one species. In 13 phyla or divisions, there was no information allowing the computation of NDS and NUS (Table S4). The phyla belonging to the Eukarya contain many conspicuous, often larger species, had members living in shallow water, and generally had a tendency to exhibit higher reported species richness. The phylum Mollusca had the highest reported value of 8,658 for the NDS. The second and third highest NDS were within the Arthropoda and Chordata, respectively. The 10 phyla with the highest totals for the NDS comprised about 85 percent of the tNDS (Figure 6). Contrastingly, phyla containing many inconspicuous, smaller species had a small NDS (Table S4).

**Table 4:** Taxonomic classification of species reported in the Japan's exclusive economic zone (EEZ).

Taxonomic group		NDS^1^	State of knowledge^2^	NIS^3^	No. experts	No. identification guides
Domain Archaea		9	1–3	ND	10	>10
Domain Bacteria (including Cyanobacteria)		843	3–5	ND	10	>10
Domain Eukarya						
Kingdom Chromista	Phaeophyta (Phaeophyceae)	304	3, 4	1	2	>3
	Other Chromista	943	3–5	ND	2	>3
Kingdom Plantae	Chlorophyta	248	3, 4	1	2	>3
	Rhodophyta	898	3–5	0	2	>3
	Angiospermae	44	4	0	2	>3
	Other Plantae	5	3, 4	ND	2	>3
Kingdom Protista (Protozoa)	Dinomastigota (Dinoflagellata)	470	3–5	0	4	>1
	Foraminifera	2,321	3–5	0	5	6
	Other Protista	1,410	1–5	0	16	>50
Kingdom Fungi		367	1–4	0	2	3
Kingdom Animalia	Porifera	745	1–5	0	1	14
	Cnidaria	1,876	1–5	1	16	>10
	Platyhelminthes	188	1–5	0	2	1
	Mollusca	8,658	1–5	11	10	>10
	Annelida	1,076	1–5	10	7	4
	Crustacea	6,232	2–5	10	>20	>10
	Bryozoa	300	5	0	2	>1
	Echinodermata	1,052	3–5	0	6	2
	Urochordata (Tunicata)	384	4, 5	2	4	>3
	Other invertebrates	1,314	1–5	2	>10	>10
	Vertebrata (Pisces)	3,790	3–5	1	15	>50
	Other vertebrates	152	3–5	0	4	>50
	Sub-Total Eukarya	32,777		39		
	Total Regional Diversity^4^	33,629		39		

1 Number of described species.

2 State of knowledge definitions: see Methods.

3 Number of known introduced species.

4 Total regional diversity including all taxonomic groups as reported in Table S3.

The total NES was at least 1,872 (Table S5). Three classes—Foraminifera, Actinopterygii and Gastropoda—contained 383, 358, and 286 endemic species, respectively. Two orders—Mysida and Gorgonacea (this is currently placed within Alcyonacea by many authors)—also had a high NES and relatively high PES, approximately 50%. Several taxa, such as Platycopioida within the Arthropoda, Nematomorpha, and Loricifera had an outstanding PES, but the NDS values for these taxa were very low, usually 1 or 2. In spite of a relatively high NDS, Phyllodocida within the Annelida and the Haptophyta had a very low NES. Totals of NUS and ENS were estimated at 121,913 and 155,542, respectively (Table S4). The total ENS is our best estimate of the total number of species currently occurring in Japanese waters. Nematoda had an exceptional NUS of 115,010, in spite of the fact that the NDS was only 70 (Table S4). This signifies that almost all species within the Nematoda are currently undescribed. Relatively well known taxa, such as the Chordata, Crustacea, and Mollusca still contained many undescribed species (Tables S3, S4). For example, Nudibranchia of the Gastropoda, Amphipoda and Isopoda of the Crustacea, and Gobiidae of the Chordata had more than 200 undescribed species. The state of knowledge varied greatly among the lower taxa, even for conspicuous organisms.

The total NIS was 39, including 11 Mollusca, 10 each of the Annelida and Arthropoda, 3 Chordata, 2 Myxozoa, and 1 of each of the Chlorophyta, Cnidaria, and Heterokontophyta (Table S6). The main presumed primary mechanism of transport is thought to be through hull fouling or in ballast water brought by ships, as well as through import as fisheries resources. On the other hand, the Japanese Association of Benthology has indicated recently that more than 40 of Japan's native species have dispersed to other nations as introduced species.

### State of knowledge

Twelve phyla—Acanthocephala, Amoebozoa, Blastocladiomycota, Chytridiomycota, Cycliophora, Glomeromycota, Heliozoa, Microsporidia, Oomycota, Opalozoa, Percolozoa, and Thaumarchaeota—were classified as Status 1 (Table S3). Many of these unknown phyla are characterized as parasites and belong to either the Fungi or Protista. Taxa containing many species targeted by fisheries and with large and conspicuous species had a tendency to be better known (Tables S3, S4). However, some unknown lower taxa were recognized even within conspicuous phyla such as Annelida, Cnidaria, Mollusca, and Porifera.

### Comparisons between NDS and the number of species in Japanese waters recorded in OBIS

Three phyla—the Nematoda, Phoronida, and Priapulida—had a PRO of almost 100% (Table 5). This means that in the present study the NDS corresponded to the NDSo, although we do not know whether the species contained are identical or not. Taxa having high NDS values, such as the Arthropoda, Chordata, Echinodermata, Heterokontophyta, and Mollusca, had low PRO values. The PRO of Annelida was moderate. The total NDSo was only 2,820. This is a very low number in spite of the very high tNDS of 33,629 in Japanese waters.

**Table 5:** Number of species recorded in Japanese waters in OBIS (NDSo) and the percentage of the species recorded in Japanese waters in OBIS (PRO).

Phylum/Division	NDS^1^	NDSo	PRO (%)
Nematoda	70	71	101
Phoronida	2	2	100
Priapulida	2	2	100
Cryptophyta	8	5	63
Annelida	1,076	529	49
Dinomastigota	470	187	40
Sipuncula	47	17	36
Ectoprocta/Bryozoa	300	85	28
Cyanobacteria	11	2	18
Hemichordata	11	2	18
Chlorophyta	248	42	17
Heterokontophyta	1,207	191	16
Arthropoda	6,393	663	10
Cnidaria	1,860	181	10
Echiura	21	2	10
Echinodermata	1,052	97	9
Chaetognatha	36	3	8
Chordata	4,330	242	6
Brachiopoda	73	4	5
Mollusca	8,658	415	5
Rhodophyta	898	39	4
Ctenophora	41	1	2
Ciliophora	530	12	2
Porifera	745	12	2
Haptophyta	304	3	1
Granuloreticulosa	2,321	11	0
	Total	2,820	

1 Number of described species.

## Discussion

According to OBIS, the total number of marine species described from the global ocean is estimated at about 230,000. The tNDS in Japanese waters is 33,629 (Table 4) and this approaches 14.6% of all marine species. The total area of Japanese waters is 4.48×10^6^ km^2^ and this is only 1.2% of the area of the global ocean, which is 360×10^6^ km^2^ in area. Also, the total volume of Japanese waters is 12×10^6^ km^3^, or 0.9% of the global ocean, which is 1,370×10^6^ km^3^ in volume. Thus, Japan's marine species richness is high considering the small area and volume of Japanese waters. The reason why such high diversity occurs is undoubtedly the varied environments existing in Japanese waters [19] including various topographical, geological, physical and chemical characteristics (see “General description of Japanese waters”). Japan's high reported species richness is also biased by investigative effort. More so than in many other countries, marine biologists in Japan have accumulated much taxonomic and ecological data concerning marine species, because the Japanese people have traditionally relied on marine fishery resources. Thus, Japanese marine species diversity seems relatively high compared with that of other areas.

In 2002, the Japanese Biota Species Number Survey Project, including all the terrestrial and marine species in Japan, was conducted by the Union of Japanese Societies for Systematic Biology (http://research2.kahaku.go.jp/ujssb/search). Japan's total number of all species on land and in its waters was estimated at about 90,000 species by this survey [20]. The taxon with the highest reported species richness was the Insecta, with about 30,000 species, and this comprised fully one-third of all of Japan's reported species. The present tNDS in Japanese waters (33,629) is also about one-third of all of Japan's reported species. The areal ratio of Japanese land (3.78×10^5^ km^2^) versus its waters (EEZ+territorial, 4.47×10^6^ km^2^) is approximately 1∶12. Thus, the richness of marine species per unit area is 7.5×10^−3^ species/km^2^, lower than the 2.4 species/km^2^ reported for land species, though the number of phyla is greater. Taxonomic and ecological studies are more advanced on land than in the sea because of logistic problems associated with research at sea, particularly concerning the deep-sea. For example, more than 500 novel species have been described over the last three decades in deep-sea hydrothermal vent areas [21]. In other words, marine species richness has a high potential to be underestimated, and species richness values potentially increase more rapidly per unit of investigative effort.

In 1981, the NDS values for several representative taxa occurring in Japanese waters were estimated by Nishimura [19]. In the 28 years since that publication, NDS values for Amphipoda, Asteroidea, Cephalopoda, Hydrozoa, Pisces, Polyplacophora, and Pycnogonida have increased considerably owing to taxonomic and ecological studies (Table 6). However, NDS values for Calcarea, Echinoidea, Scyphozoa, and Sipuncula have remained the same or have decreased. Recently, several researchers have become active in Japan working on the Calcarea, Echinoidea, and Scyphozoa, so their NDS values are expected to increase in the near future. However, the number of taxonomic experts studying the Sipuncula is too few—only a single researcher within Japan.

**Table 6:** Comparison of number of described species in selected taxa between present study and a previous study of Nishimura (1981) [19].

Taxon			NDS^1^ of previous study [19]	NDS of present study	Increase of NDS^2^
Phylum	Class	Order			
Chordata	Pisces		2700	3790	1090
Cnidaria	Hydrozoa		315	523	208
Chordata	Ascidiacea		281	313	32
Echinodermata	Ophiuroidea		ca. 260	308	48
Echinodermata	Echinoidea		192	161	−31
Echinodermata	Asteroidea		167	280	113
Platyhelminthes	Polycladida	Polycladida	149	150	1
Porifera	Calcarea		130	130	0
Mollusca	Cephalopoda		125	204	79
Arthropoda	Pycnogonida		67	153	86
Sipuncula			58	47	−11
Arthropoda	Crustacea	Amphipoda	57	544	487
Mollusca	Polyplacophora		56	129	73
Brachiopoda			55	73	18
Arthropoda	Crustacea	Stomatopoda	41	56	15
Cnidaria	Scyphozoa		38	37	−1
Echiura			17	21	4

1 Number of described species.

2 Difference between NDS reported in Nishimura (1981) [19] and NDS of the present study.

In Japanese waters, the NES is not great (Table S5), being only 5.6% of the tNDS. Because most marine species spend a part of or their whole life cycle within the pelagic zone, the number of endemic species in general in the oceans is few. An exception to this rule is the many endemic species that have been reported from unique habitats such as submarine caves, deep-sea hydrothermal vents, methane seeps, sunken wood, and whale falls [7], [22], [23].

Additionally, the strong ocean currents in Japanese waters (Figure 3) obviously allow marine organisms to disperse over a wide distributional range. For example, the Kuroshio Current transports marine organisms from the equatorial Pacific into Japanese waters, while the Oyashio Current transports them from the northeast Pacific [2]. Decapoda and Echinoidea in Japanese waters tend toward a high degree of endemism according to one paleontological study [19]. However, the PES of Decapoda in the present study is only 1.1% and is therefore not in agreement with this previous study.

NUS values were estimated for only 30 of the 93 phyla (Table S4). Total NUS in Japanese waters—121,913—is obviously an underestimate because we could not estimate NUS for many taxa containing predominantly inconspicuous, smaller species. The NUS is approximately four times the tNDS. In the Nematoda, incredibly high species diversity and the existence of numerous undescribed species have been suggested by previous investigations [24], [25]. The present study also suggests an exceptional NUS in the Nematoda within Japanese waters. In spite of this high species richness in the Nematoda, the number of taxonomic experts in this group is far too few in Japan. Taxa showing a smaller NUS than NDS suggest that they are relatively well known taxonomically, although the existence of cryptic species is still possible because of a lack of good morphological characteristics in some taxa. Examples of the above taxa include the Annelida, Arthropoda, Chordata, Cnidaria, Granuloreticulosa, Mollusca, and Radiozoa.

According to our study, the percentage of NUS versus NDS is low in the Rotifera, Cercozoa, Chordata, and Cyanobacteria, at 0.3%, 2.8%, 7.5%, and 9.1%, respectively. At a glance, this would suggest that the Cercozoa and Cyanobacteria are well known taxonomically. This assessment is probably erroneous, however, because a concentrated sampling effort has been lacking, and too few samples of these taxa have been studied.

In spite of the fact that Chordata is the most taxonomically well known taxon in Japanese waters, a high number of undescribed species was estimated. In particular, the family Gobiidae within the Actinopterygii contains 216 undescribed species, versus 316 described species. The high ratio of NUS to NDS in the Gobiidae is probably due to (1) difficulty of sample collection, (2) lack of good morphological characteristics enabling ready species identification, and (3) lack of funding for taxonomic studies [26]. These reasons are common to many groups with a high NUS-to-NDS ratio.

Another factor that needs to be borne in mind is that there may be a higher reported ratio of NUS to NDS when a taxonomic expert is actively working on a group, than when this is not the case. For example, before 1999 a total of 28 siphonophore species were reported from Japanese waters according to local taxonomic treatises [27], and many of these were reported under obsolete scientific names. Since 1999, it has become apparent that at least 65 species of siphonophore species occur [28], [29], and that at least 9 of these are undescribed, sometimes at the genus or even family level [28].

As maritime trade has increased, so have introductions of invasive species into foreign waters throughout the world. Introduced species can have severe impacts on local marine ecosystems and on fisheries, shipping and power stations [30]–[33]. At least 39 recently introduced species occur in Japanese waters (Table S6). Concrete examples include the gastropod *Nassarius sinarus*, which detrimentally affects mariculture [33], and the gastropod *Euspira fortunei*, which has also had an impact on the native bivalve *Ruditapes philippinarum* [34]. *Mytilopsis sallei, Mytilus galloprovincialis, Perna viridis* in the Mollusca, *Hydroides elegans* and *Hydroides dianthus* in the Annelida, and *Balanus amphitrite* and *Balanus eburneus* in the Arthropoda have had a highly detrimental effect on oyster aquaculture. These species attach to the hulls of ships and to the intake pipes of power plants. *Balanus amphitrite, Balanus glandula*, and *Carcinus aestuarii* of the Arthropoda, and *Mytilus galloprovinciali* of the Mollusca also have invaded several areas and excluded native species. *Caulerpa taxifolia* of the Chlorophyta, called the “Killer Algae,” has spread from the Indian Ocean to areas off the coasts of Australia, into the Mediterranean, and along the coasts of the United States, affecting many native marine ecosystems. This species was also recently introduced into Japanese waters. Transport within the ballast water of large ships is one of the major mechanisms responsible for the dispersal of nonnative marine organisms around the world. Japan is one of the largest nations for maritime trade, and ships traveling either to or from Japan account for about 10% of the total ballast water around the world [32]. This indicates that Japan has a high potential for causing the introduction of invasive species to other regions. To mitigate or avoid introductions of invasive species, the Invasive Alien Species Act was promulgated in Japan in 2004.

### State of knowledge

**Figure 7:**
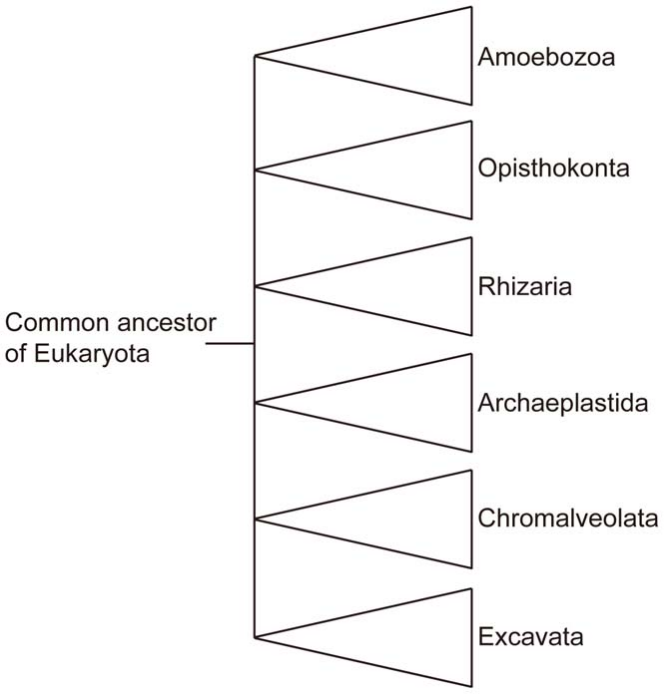
Supergroups of eukaryotes based on molecular data, after six supergroups of eukaryotes [**36**].

Taxa containing many conspicuous, larger species have a tendency to be well known taxonomically and ecologically. On the other hand, many taxa of which our knowledge is only of elementary status (State of Knowledge 1) are recognized to occur in Japanese waters (Table S3). Those less well known taxa include the Acanthocephala, Amoebozoa, Apicomplexa, Cycliophora, Heliozoa, Oomycota, Opalozoa, and Percolozoa. Except for the Acanthocephala, the remaining taxa predominantly contain small species. Difficulties in sample collection and morphological identification due to the organisms being so small, as well as the lack of taxonomic expertise in Japan (and indeed around the world), are the major reasons for our lack of knowledge about these taxa. To solve the problems arising from difficulties in identification based on morphology, modern molecular and microscopic techniques can be a useful tool. Recently, Eukarya were indicated to be classifiable into six major supergroups based on their molecular phylogeny [35], [36] (Figure 7). Amoebozoa is one of the supergroups, although in the case of Heliozoa, it is as yet unclear to which group it belongs. Each supergroup contains many small species, commonly called protists. Small species, including these protists, seem to exhibit a much higher species diversity than large species [36]. Thus, to understand diversity and evolution in the Eukarya, it is important to gather more taxonomic and systematic information on taxa containing many small species.

The present study has revealed that our state of knowledge concerning the taxonomy and ecology of many taxa in Japanese waters ranges from fairly well known to almost totally unknown. To more easily compare the state of knowledge for each taxon, we classify their state of knowledge into three categories—known, mostly unknown, unknown—for each phylum or division based on the following definitions; Known: almost all orders, superorders, infraorders, suborders, or families were estimated to have a status of either 5 or 4. Unknown: almost all orders, superorders, infraorders, suborders, or families were estimated to have a status of 1. Mostly Unknown: neither known nor unknown. The relative numbers of taxa belonging to each category were 22 known, 42 mostly unknown, and 14 unknown (Table 7). Japan therefore has a high percentage of mostly unknown or unknown taxa. It is necessary to encourage the development of taxonomists who specialize in these taxa in Japan.

**Table 7:** Current taxonomic status, Known, Mostly unknown and Unknown, for each Phylum or Division.

Taxonomic status			
Known	Mostly unknown		Unknown
Acoelomorpha	Acidobacteria	Hemichordata	Acanthocephala
Bacteroides	Actinobacteria	Heterokontophyta	Amoebozoa
Cercozoa	Annelida	Kinorhyncha	Apicomplexa
Chaetognatha	Aquificae	Loricifera	Blastocladiomycota
Chlorophyta	Arthropoda	Metamonada	Chytridiomycota
Choanozoa	Ascomycota	Nematoda	Cycliophora
Chordata	Basidiomycota	Nematomorpha	Glomeromycota
Ctenophora	Brachiopoda	Nemertea	Heliozoa
Cyanobacteria	Ciliophora	Nitrospirae	Microsporidia
Dicyemida	Cnidaria	Orthonecta	Oomycota
Echinodermata	Crenarchaeota	Phoronida	Opalozoa
Ectoprocta/Bryozoa	Cryptophyta	Placozoa	Percolozoa
Firmicutes	Deferribacteres	Platyhelminthes	Priapulida
Granuloreticulosa	Deinococci	Porifera	Thaumarchaeota
Haptophyta	Dinomastigota	Proteobacteria	
Labyrinthulomycota	Echiura	Sipuncula	
Magnoliopsida	Entoprocta	Tardigrade	
Mollusca	Euglenophyta	Thermotogae	
Myxozoa	Euryarchaeota	Verrucomicrobia	
Radiozoa	Gastrotricha	Zygomycota	
Rhodophyta	Glaucophyta		
Rotifera	Gnathostomulida		

### Databases concerning marine life in Japan

OBIS is a powerful tool and data source for marine biogeographical and other studies. Unfortunately, the total PRO is quite low at only 8.4%. Potentially, several databases concerning the diversity or distribution of marine organisms exist in Japan. Some of them are listed as follows;

- Algae resource database: http://www.shigen.nig.ac.jp/algae/
- Aves: http://www3.town.haboro.hokkaido.jp/seabird/
- Biological Information System for Marine Life (BISMaL): http://www.godac.jp/bismal/searchSpecies.jsf
- CMarZ-Asia Database: http://cmarz-asia.org/db/
- Database for aquatic-vertebrate science: http://research.kahaku.go.jp/zoology/photoDB/
- Illustrated Guide of Marine Mammals: http://svrsh1.kahaku.go.jp/m/mm/
- Japan Collection of Microorganisms: http://www.jcm.riken.jp/
- Japanese Biota Species Number Survey: http://research2.kahaku.go.jp/ujssb/search
- Japan Oceanographic Data Center (JODC): http://www.jodc.go.jp/index_j.html
- NaGISA Database: http://www.nagisa.coml.org/
- NITE Biological Resource Center: http://www.nbrc.nite.go.jp/
- One Hundred Seaweeds of Japan: http://research.kahaku.go.jp/botany/seaweeds/JS100Home.html
- Protist Information Server: http://protist.i.hosei.ac.jp/protist_menu.html
- Reptilia: http://mail2.nara-edu.ac.jp/~inoue/NNM/hatyuurui/wamei-h.html
- Tardigrades: http://homepage3.nifty.com/cxj11255/jtard/index.html

Of these, NaGISA, CMarZ-Asia and the JODC databases directly or indirectly link to OBIS. The BISMaL adopts a common Darwin Core schema to link to OBIS and the Global Biodiversity Information Facility. Most other databases are operated in the Japanese language and have different data schema, so it is not easy to link them to OBIS. To encourage linkages between Japan's databases and OBIS, we need to establish a regional OBIS node in Japan in the near future.

We expect rapid changes in the marine biota in Japanese waters: (1) declining wild fish catches, (2) increasing aquaculture, (3) changes in harvesting of specific species, (4) changes in harvested areas, (5) food web changes, (6) shifts in diversity at population, species, and genetic levels, (7) species extinction, population extirpation, (8) changes in species distribution: contraction, expansion, and range shifts, (9) changed traffic patterns of animal migrations, (10) introduction of exotic species, (11) changes in nutrient cycles, (12) changes in habitat provision, (13) changes in surface primary productivity and carbon fluxes to the seafloor, and so on. However, our knowledge is still too elementary for proper understanding of the roles played by marine life in ecosystem services and functioning. There are numerous unexplored areas, even in Japanese waters, especially in the deep sea. Japan is a so-called maritime nation and is in a particularly suitable geographic situation for marine biological investigations. In particular, deep-sea troughs and trenches are concentrated in Japanese waters. To investigate these deep-sea areas, several tools such as autonomous underwater vehicles (AUVs), HOVs, remotely operated vehicles (ROVs), and other research vessels have been developed and deployed by Japan. Additionally, the ocean drilling ship *Chikyu*, under the Integrated Ocean Drilling Program (IODP), also started operations in 2007. One of the targets of the IODP is to investigate the deep biosphere below the seafloor, the diversity of which remains unknown. Japan, as a maritime nation, has an important responsibility to contribute to our understanding of life in the oceans.

Finally, this study provides the baseline data for biodiversity studies in Japanese waters. This is an important contribution not only for science but also for the general public including NGO, NPO and policy-making stakeholders. Therefore, we have attached alternative language (Japanese) versions (Alternative Language Article S1 and S2).

## Supporting Information

Table S1 Brief history of marine biological activities in Japan.
(0.04 MB XLS)

Table S2 Contributors for species diversity estimation.
(0.03 MB XLS)

Table S3 List of species diversity including the number of described species (NDS), the number of endemic species (NES), the number of undescribed species (NUS), expected number of species (ENS), the number of introduced species (NIS), the number of taxonomic experts, the number of identification guides, and state of knowledge in each taxon in Japanese waters.
(0.25 MB XLS)

Table S4 Number of described species (NDS), number of identified but undescribed species (NUS) and expected number of species (ENS) in each phylum or division in Japanese waters.
(0.03 MB XLS)

Table S5 Number of endemic species (NES) and the percentage of endemic species in Japanese waters.
(0.03 MB XLS)

Table S6 List of species introduced into Japanese waters, their presumed primary mechanism of transportation and origin.
(0.02 MB XLS)

Alternative Language Article S1 Alternative Language Japanese Article S1, part 1 of 2
(7.55 MB PDF)

Alternative Language Article S2 Alternative Language Japanese Article S2, part 2 of 2
(5.80 MB PDF)
