# Supplementary material for: Marine Biodiversity in Japanese Waters
Source: PLoS One. 2010 Aug 2;5(8):e11836. doi: 10.1371/journal.pone.0011836 (PMC2914005; doi:10.1371/journal.pone.0011836)
Supplement: Alternative Language Article S1 — Alternative Language Japanese Article S1, part 1 of 2 (7.55 MB PDF) [file pone.0011836.s007.pdf]

Marine Biodiversity in Japanese Waters
（日本近海の生物多様性）

藤倉克則: 海洋研究開発機構 海洋・極限環境生物圏領域，〒237-0061 横須賀市夏島町 2-15, e-mail: fujikura@jamstec.go.jp

Dhugal Lindsay: 海洋研究開発機構 海洋・極限環境生物圏領域，〒237-0061 横須賀市夏島町 2-15, e-mail: dhugal@jamstec.go.jp

北里 洋: 海洋研究開発機構 海洋・極限環境生物圏領域，〒237-0061 横須賀市夏島町 2-15, e-mail: kitazatoh@jamstec.go.jp

西田周平: 東京大学大気海洋研究所，〒277-8564 千葉県柏市柏の葉 5-1-5, e-mail: nishida@aori.u-tokyo.ac.jp

白山義久，京都大学フィールド科学教育研究センター瀬戸臨海実験所，〒649-2211 和歌山県西牟婁郡白浜町 459, e-mail: meiobenthos2007@yahoo.co.jp

要旨 Abstract

日本近海（領海と排他的経済水域）の海洋生物多様性研究のベースライン情報を集めるために，我々は日本近海の海洋環境や地学背景を概観し，出現種数，海洋生物研究の歴史，分類群毎の研究進捗レベル，固有種数，将来出現が記録される予測種数，外来種数，分類学研究者数，種同定に用いられる文献数を評価した．そして，日本近海からは，これまでに33629種の生物が出現していることがわかった．分類群毎の研究進捗レベルは大きな差があり，とりわけ人と直接的な関係が少ないものや小型種を多く含む分類群において，データが著しく少ない傾向が見られた．今後，分類学研究や生態学研究が進み，日本近海から出現が記録される予測種数は121913種となった．これまで出現している種数と，予測種数をあわせると155542種となり，これが日本近海に生息している生物の総種数と評価された．同時に未だ70%以上の種が未記載種もしくは分布記録がないことが示唆された．自然分布では日本近海には出現しないが，人為的に移入してきた外来種は39種に達した．このような側面から，包括的に日本近海の海洋生物種数を評価する試みは本研究が初めてと思われる．日本近海の生物多様性データは，アジア–太平洋の中では比較的多いと思われるが，サンゴ礁，海溝，流氷，メタン湧水，熱水噴出など多様な生息環境があり，本研究で多様性や分布データが少ない分類群がまだ多数あることが示唆された．人間活動による地球環境の変化は，海洋生物の多様性や生態系に大きな影響を与えると考えられている．日本は，四方を海に囲まれ海洋生物研究に適した環境にある．また，研究調査船を多数運用していることからも，日本が海洋生物研究を推進し生物多様性や生態系の理解に貢献することが重要である．

はじめに Introduction

人類は海洋生物を利用している．人類と海洋生物が持続的に共存するためには，海の生物多様性や生態系の機能を理解し，地球環境の変化や人間活動が海に及ぼす影響を評価することが重要である．そのためには，海の生物多様性を分類し目録を作ることが不可欠であるが，十分に進んでいるとは言えない [1]． そこで，海の生物多様性や生態系の理解に貢献するために，海洋生物の多様性，分布，量を評価することを目的に国際プロジェクトネットワーク「海洋生物のセンサス」Census of Marine Life （CoML）が2000年から組織され活動がはじまった（http://www.coml.org/）．広大な海からデータを得るには，世界各国や地域の活動が重要でCoMLは12の国や地域にNational and Regional Implementation Committees （NRICs）を設置している．NRICsの役割の一つは，各国や地域の海洋生物多様性研究の情報を集めることである．本研究は，日本のNRICsの活動の一環として取り組んだものである．

日本では古くから海洋生物研究が行われてきており，近海には様々なタイプの生息環境があるため，種多様性は高いと言われている [2]．日本は，狭い国土に多くの人口がいるが，四方を海に囲まれるため伝統的に多くの海産物を食糧としてきた． FAOの統計によると，2001–2003年における日本人１人あたりの水産物消費量は66.9 kg/年で，これは世界平均の約4倍となっている．日本では海洋生物の重要性は広く認識されており，海洋生物を対象とした分類学，生態学，生理学といった研究は，積極的に取り組まれてきている．このような背景にある日本は，地球環境変動や人間活動に伴う海洋生態系変動の影響を強く受ける．本研究では日本近海の海洋生物多様性の現状を理解するためのベースラインを作るために，日本近海（領海と排他的経済水域EEZを含む海域）の海洋環境や地学背景を概観し，日本近海からの出現種数（NDS），固有種数（NES），将来出現が記録される予測種数（NUS），外来種数（NIS），海洋生物研究の歴史概要，分類群毎の研究進捗レベル，分類学研究者数，種同定に用いられる文献数を評価した （Table 1，2 に本研究で使用する略語のリストを示した）．

Table 1. 本研究で用いる略語一覧.

略語	説明
AUV	自律型無人探査機
EEZ	排他的経済水域
ENS	推定種数
HOV	有人潜水調査船
ND	データ無し
NDS	出現種数
NDSo	日本の EEZ 内から記録されている OBIS 内種数
NES	固有種数
NIS	外来種数
NUS	今後出現が記録される種数
PES	NDS に対する NES の割合
PRO	NDS に対する NDSo の割合
ROV	無人探査機
tNDS	日本近海の海洋生物総種数

Table 2. 本研究で用いる研究機関や組織名略語の一覧.

略語	研究機関や組織名
AMSL	阿嘉島臨海研究所
AIST	産業技術総合研究所
BIK	黒潮生物研究財団
BISMaL	Biological Information System for Marine Life
CoML	海洋生物のセンサス
GBIF	地球規模生物多様性情報機構
HJC	函館短期大学
IODP	統合国際深海掘削計画
ISU	石巻専修大学
JAMSTEC	海洋研究開発機構
JMA	気象庁
JODC	日本海洋データセンター
JSNFRI	水産総合研究センター
KMNH	北九州市立いのちのたび博物館
KMPC	串本海中公園センター
LBM	滋賀県立琵琶湖博物館
NHMIC	千葉県立中央博物館
NIES	国立環境研究所
NIPR	国立極地研究所
NITE	製品評価技術基盤機構
NMNS	国立科学博物館
NRICs	National and Regional Implementation Committees
NRIFS	中央水産研究所
OBIS	Ocean Biogeographic Information System
OMNH	大阪市立自然史博物館
ORI	東京大学海洋研究所
SFL	巣鴨有孔虫研究室
SNF	西海区水産研究所
TAT	東京農工大学
TNFRI	東北区水産研究所
TSM	富山市科学博物館
TUMSAT	東京海洋大学
UBC	ブリティッシュコロンビア大学
YNU	横浜国立大学

日本近海の概要 General description of Japanese waters

地学背景 Topographical and geological characteristics

日本は北半球の太平洋西側に位置し，陸域で他国と接していない．日本列島は，20°30′N から45°30′N，123°E に 150°E にあり，気候帯は亜寒帯から熱帯にわたる．日本のEEZは，ほぼ17°N から48°N, 122°E から158°Eの範囲にある．陸域の面積は3.78 x 10^5 km²と小さいが，EEZの面積は陸域の約11倍4.05 x 10^6km²となり，これは世界第6位の広さである．日本近海の最深部は伊豆・小笠原海溝の水深9780mである．

日本は島嶼国で，北から南へ主に北海道，本州，四国，九州が連なる．北海道の北東部には千島列島，本州の南には伊豆・小笠原諸島，九州の南には琉球列島が広がる．日本列島は太平洋北部と，オホーツク海，日本海，東シナ海といった縁海に囲まれ（Figure 1），海岸線の長さは約30,000kmになる．EEZ内における水深1000mごとの面積をみると，各水深帯の面積がほぼ等しい割合で存在している（Figure 2）．

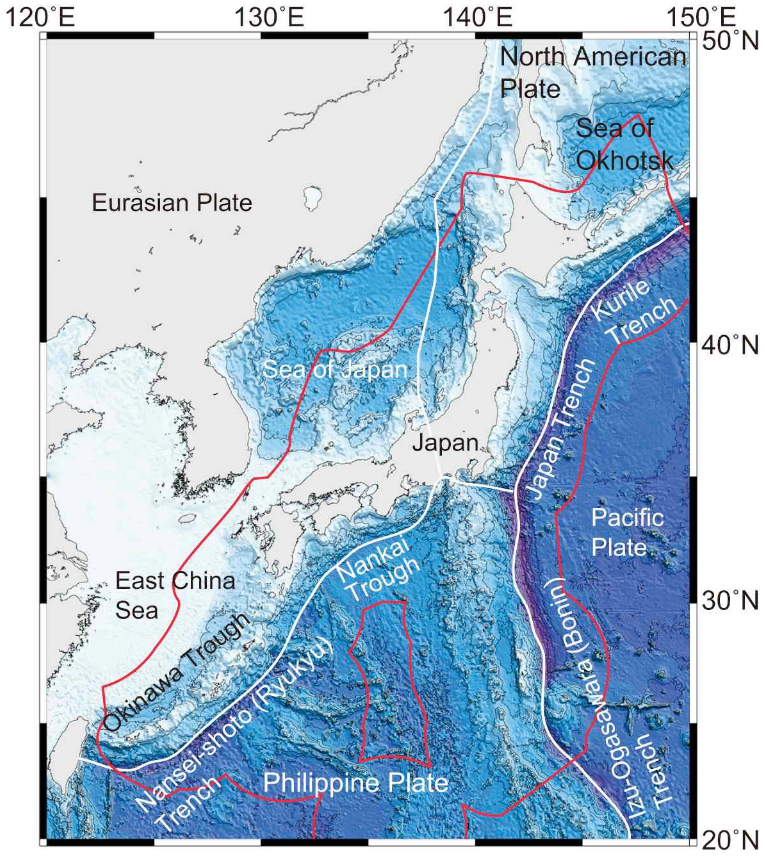

Figure 1. 日本近海の海底地形．白ラインはプレート境界，赤ラインは日本の排他的経済水域の境界を示す.

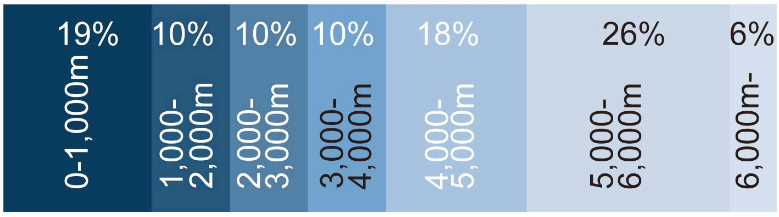

Figure 2. 日本の排他的経済水域内における水深1000m 毎の面積の割合.
http://www.sof.or.jp/jp/news/101-150/123_3.php#1から作図

日本近海では，沿岸から深海底にわたって多数の地学研究がある [3, 4]．海岸線には湾，砂浜，干潟，内海，岩礁といった様々な地形があり，都市部は埋め立て地が普通に見られる．大きな湾は，北海道に内浦湾，本州太平洋側に伊勢湾，三河湾，陸奥湾，相模湾，駿河湾，東京湾，本州日本海側に富山湾，若狭湾，四国に土佐湾，九州に有明湾，鹿児島湾がある．1ヘクタールを超す干潟は約30あり，有明湾の干潟が最も広い．代表的な内海は，本州，四国，九州に囲まれた瀬戸内海で，面積は約20000 km^2 で720の小さな島がある．

日本近海は，4枚のプレート（ユーラシアプレート，北米プレート，太平洋プレート，フィリピンプレート）上にある（Figure 1）．太平洋プレートは，東太平洋海膨で形成され日本側に移動し，一部は日本海溝や千島海溝で北米プレートの下に沈み込む．残りの部分は伊豆・小笠原海溝でフィリピンプレートの下に沈み込む．フィリピンプレートの北部は北米プレートの下に沈み込み，北西部は南海トラフや南西諸島（琉球）海溝でユーラシアプレートの下に沈み込む．これらのプレート収斂は島弧–海溝系させ，活火山，海溝，トラフを形成する．相模トラフ，駿河トラフ，そして多くの海底火山がある沖縄トラフや七島硫黄島海嶺もこの島弧–海溝系に起因する．全体的に見て，日本の海底地形は海溝やトラフが多数ある窪地地形が優占する．

物理・化学的特徴 Physical and chemical characteristics

日本近海に流れる主な海流は，暖流の黒潮と対馬海流，寒流の親潮である（Figure 3）． 黒潮は東シナ海から日本列島の太平洋側を走り，横幅約200km，深さ700mにまで達することもある太平洋で最大規模の海流である [5]．流速は，150–250cm/s，流量は5×10^7 ton/sと見積もられる．対馬海流は，九州沖で黒潮から枝分かれし日本海に流れ込む．親潮は，太平洋側を北海道沖から本州にかけて南下し，流速は20 cm/s，流量は黒潮に比べはるかに小さい．日本近海の表面水温は四季に応じて変化し，夏季が最も高く冬季が最も低い（Figure 4）．水温の鉛直構造は，太平洋側と日本海側で大きく異なり，日本海側が低くなる （Figure 5）．日本近海の気候は，オホーツク海や東部北海道沖が含まれる亜寒帯から，琉球列島，小笠原諸島の南部が含まれる熱帯の6区分にわたり（Figure 3），全体としてはインド–西太平洋区分に属する．

日本近海には様々なタイプの海洋生態系が，それぞれの環境に応じて形成されている．ユニークな生態系としては，例えば冬季のオホーツク海に認められる流氷生態系，琉球列島や小笠原諸島に代表されるサンゴ礁生態系，超深海底や中・深層を含む深海生態系などが挙げられる．また，プレート運動に伴って形成される湧水生物群集や熱水噴出孔生物群集といった化学合成生態系も，プレート境界域に沿って多数発見されている [6, 7]． 湧水生物群集は，日本海溝，南海トラフ，南西諸島（琉球）海溝，相模湾，駿河湾に多く，熱水噴出孔生物群集は伊豆・小笠原諸島海域や沖縄トラフに多い．

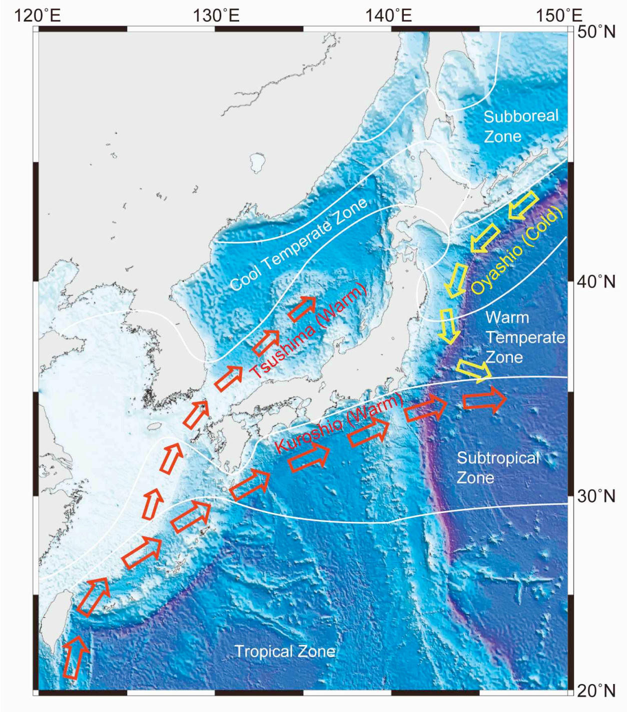

Figure 3. 日本近海の海流と気候区分．赤い矢印は暖流の黒潮と対馬海流．黄色の矢印は寒流の親潮．
