# Supplementary material for: Marine Biodiversity in Japanese Waters
Source: PLoS One. 2010 Aug 2;5(8):e11836. doi: 10.1371/journal.pone.0011836 (PMC2914005; doi:10.1371/journal.pone.0011836)
Supplement: Alternative Language Article S2 — Alternative Language Japanese Article S2, part 2 of 2 (5.80 MB PDF) [file pone.0011836.s008.pdf]

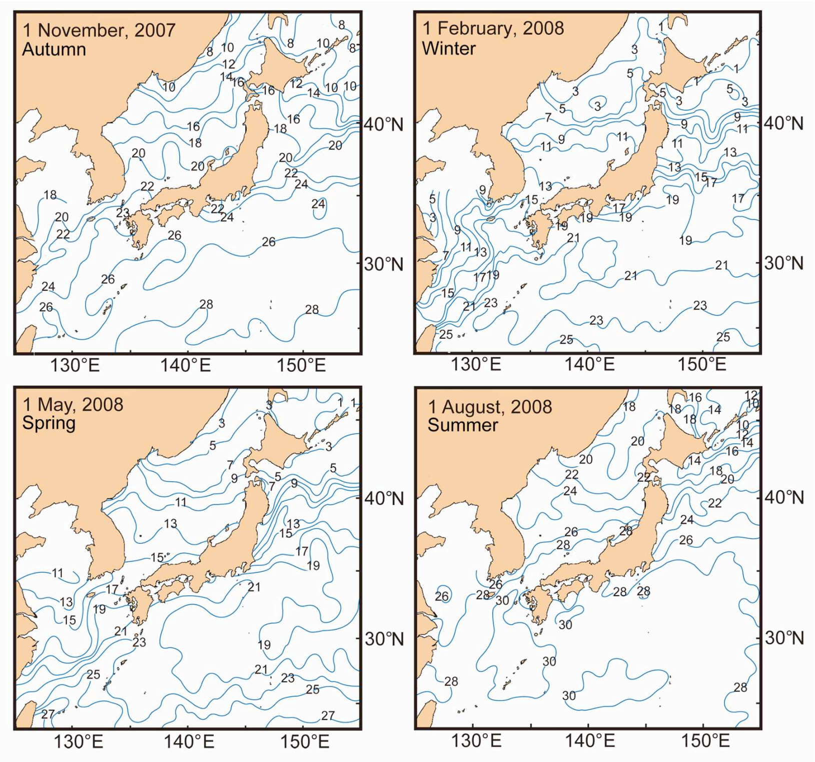

Figure 4. 日本近海の季節毎の表面水温.

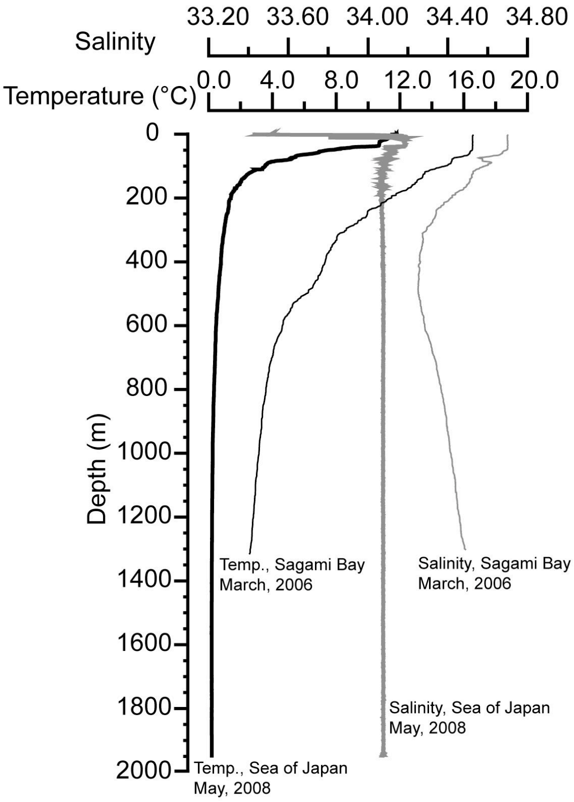

Figure 5. 太平洋側の相模湾と日本海の鉛直水温と塩分濃度の比較.

日本の海洋生物研究史（Table S1）Brief history of research in Japan

日本は，1616年から1858年まで鎖国制度により外国との交流を制限していた．この鎖国期間には，生物学データは本草学や俳句の季語として集積されていた．鎖国期間中も貿易を許されていたオランダは，日本近海の海洋生物を自国に持ち帰り，それらのサンプルはヨーロッパの研究者によって研究され，モノグラフとなった．1858年以降，ドイツとアメリカから何人かの研究者が日本の大学で博物学の講義を行った．彼らは，日本の海洋生物相を研究した．第二次世界大戦前には，博物館や海洋研究機関が設立され，日本における海洋生物研究の基礎が培われた．

1875年にはイギリスのチャレンジャー号によって，相模湾や房総沖の調査が行われ，またチャレンジャー号の研究者は市場などでサンプルとして様々な海洋生物を購入した．1906年にはアメリカのアルバトロス号によって相模湾や駿河湾の調査が行われた．1929年には海洋生物研究を目的とした世界初の有人潜水調査船「西村式豆潜水艇1号」が作られ，これにはディーゼルエンジン，サンプル採集装置，水中ライト，観察窓，水中通話器などが装備されていた．1955年以降には，大型の海洋研究船も運用され，大規模な海洋生物調査や国際プロジェクトへの参加が推進された．昭和天皇は，海洋生物研究者でもあり，共同研究者とともに節足動物，ホヤ類，刺胞動物，棘皮動物，軟体動物，海綿動物のモノグラフを積極的に出版した [8–18].

方法 Methods

種数評価 Species richness estimation

日本近海（領海とEEZ）の海洋生物種数を分類階級の目レベルで，出現種数（NDS），固有種数（NES），今後出現が記録される種数（NUS），外来種数（NIS）について評価した．目レベルに分類することができない分類群については，上目，下目，亜目もしくは科レベルで，それぞれの数を評価した．さらに，分類学研究者数，種同定に役立つ文献数（モノグラフ，図鑑，論文，ホームページ）についても情報を収集した．１つの分類群に多数の分類学研究者数を含む場合は2名を示した．種同定に用いられる文献は，種同定に参照できる図鑑やモノグラフを対象にした [1].

また，分類群毎の研究進捗レベルを以下の定義に基づき5段階に評価した．

Status 5：データがとても豊富な分類群．以下の条件を全て満たすもの．（1）日本近海に出現する種のうち80%以上もしくは100種以上が科学的文献に記録されていること．（2）種同定に役立つ文献が過去20年以内に公表されていること．（3）専門とする分類学研究者が少なくとも1人は日本にいること．

Status 4：データが豊富な分類群．（1）日本近海に出現する種のうち70%以上もしくは10種以上が科学的文献に記録されていること．（2）種同定に役立つ文献が公表されていること．（3）専門とする分類学研究者が少なくとも1人は日本にいること．

Status 3：データが少ない分類群．（1）日本近海に出現する種のうち50%以上もしくは10種以下が科学的文献に記録されていること．（2）種同定に役立つ文献が公表されていること．（3）専門とする分類学研究者が日本にいないこと．

Status 2：データがとても少ない分類群．以下の条件の少なくとも１つにあてはまるもの．（1）日本近海に出現する種のうち50%以下もしくは数種が科学的文献に記録されていること．（2）種同定に役立つ文献や専門研究者が認められないこと．

Status 1：不明な分類群．以下の条件のいずれかにあてはまるもの．（1）日本近海に出現記録がないこと．（2）関連する情報が認められないもの．

本研究に用いるデータは，Table S2に挙げた多くの研究者から提供を受けた．それぞれの分類群のNDSを積算し日本近海の海洋生物総種数（tNDS）とした．NUSは，Table 2Sに挙げた研究者が研究中のサンプル，経験，知識から予測した値を用いた．また，NDSとNUSを合算し，それぞれの分類群が含む推定種数（ENS）を評価した．そして，tNDSとすべてのNUSを合算し日本近海に生息する推定総種数を求めた．

これまでに日本近海にのみ出現記録がある種を固有種とした．NDSに対するNESの割合（PES）をPES = （NES/NDS） x 100に従い求めた．外来種は，自然環境下では日本近海に生息していない種が，人為的行為により日本近海に移入してきたものとした．外来種に関しては，種数（NIS）だけでなく種名，それぞれの移入過程，移入元を示した．

最新の日本近海に分布する海洋生物の多様性や分布を包括した文献，データベースが見あたらない．そこで，専門研究者のいない分類群は，日本分類学会連合がまとめた日本産生物種数調査や（http://research2.kahaku.go.jp/ujssb/search），文献などからのデータを集めた．海洋生物研究は，潮間帯だけでなく外洋や深海域でも行われるため，現在活動している調査船のうち500トン以上のものについてTable 3に示した．

Table 3. 日本における 500 トン以上の海洋調査船リスト.

海洋調査船名	大きさ(トン)	所属機関	主目的
望星丸	2,174	東海大学	Multi-purpose missions
ちきゅう	57,087	海洋研究開発機構	Drilling
白鳳丸	3,991	海洋研究開発機構	Multi-purpose missions
北光丸	568	水産庁	Fisheries science
かいれい	4,628	海洋研究開発機構	Support of remotely operated vehicle
かいよう	3,350	海洋研究開発機構	Multi-purpose missions
開洋丸	2,942	水産庁	Fisheries science
啓風丸	1,882	気象庁	Oceanography
敬天丸	860	鹿児島大学	Fisheries science, Oceanography
耕洋丸	2,703	水産庁	Fisheries science, Oceanography
みらい	8,687	海洋研究開発機構	Multi-purpose missions
長崎丸	842	長崎大学	Fisheries science, Oceanography
なつしま	1,739	海洋研究開発機構	Support of remotely operated vehicle
おしょろ丸	1,792	北海道大学	Fisheries science, Oceanography
凌風丸	1,380	気象庁	Oceanography
神鷹丸	649	東京海洋大学	Fisheries science, Oceanography
しらせ	12,500	国立極地研究所	Antarctic Expedition
照洋丸	2,494	水産庁	Fisheries science
俊鷹丸	1,228	水産庁	Fisheries science
蒼鷹丸	1,234	水産庁	Fisheries science
淡青丸	610	海洋研究開発機構	Multi-purpose missions
天鷹丸	1,020	水産庁	Fisheries science
海鷹丸	1,886	東京海洋大学	Fisheries science, Oceanography
若鷹丸	692	水産庁	Fisheries science
陽光丸	608	水産庁	Fisheries science
よこすか	4,439	海洋研究開発機構	Support of human occupied vehicle

OBIS に収録されている情報と NDS の比較 Comparisons between NDS and the number of species recorded from Japanese waters in OBIS

Ocean Biogeographic Information System （OBIS: http://www.iobis.org/）は「海洋生物のセンサス」（CoML）のもとに構築された海洋生物地理情報データベースで，世界中の海洋生物の多様性や分布を解析することに役立つ． 2009年6月6日にOBISを用いて，日本のEEZ内から記録されている各分類群のOBIS内種数（NDSo）を求め，さらにOBISのpolygonによって日本のEEZ内の総種数を求めた．そして，本研究で得られたNDSとNDSoを比較するために，NDSに対するNDSoの割合（PRO）をPRO = （NDSo / NDS） x 100で求めた．

結果 Results

日本近海の生物種数 Species richness in Japanese waters

日本近海の海洋生物種数に関するデータのうち，出現種数（NDS），分類群毎の研究進捗レベル，外来種数（NIS），分類学者数，種同定に役立つ文献数をTable 4に示した．目レベル（もしくは下位の分類群）のNDS，固有種数（NES），今後出現が記録される種数（NUS），推定種数（ENS），NIS，分類学者数，種同定に役立つ文献数，研究進捗レベルはTable S3に示した．日本近海の海洋生物総種数（tNDS）は33629となった．全79門のうち，66門が1種以上の種が認められたが，13門については情報が得られずNDSとNUSは評価できなかった（Table S4）．

真核生物Eukaryaの門は，人との係わりがあり大型で浅海に生息するため多くの種数が記録されている傾向にあった．軟体動物門MolluscaのNDSは8658となり最も高い値を示し，2番目が節足動物門Arthropoda，3

番目が脊索動物門Chordataとなった．NDSが大きな上位10までの門だけで，日本近海の海洋生物総種数（tNDS）の85%を占めた（Figure 6）．一方で，人との係わりが少なく小型種を多く含む門のNDSは小さくなった（Table S4）．

Table 4. 日本近海における海洋生物の出現種数，研究進捗レベル，外来種数，分類学研究者数，種同定に役立つ文献数の概要.

分類群		NDS[1]	State of knowledge[2]	NIS[3]	分類学研究者数	種同定に役立つ文献数
Domain Archaea		9	1-3	ND	10	>10
Domain Bacteria (including Cyanobacteria)		843	3-5	ND	10	>10
Domain Eukarya						
Kingdom Chromista	Phaeophyta (Phaeophyceae)	304	3, 4	1	2	>3
	Other Chromista	943	3-5	ND	2	>3
Kingdom Plantae	Chlorophyta	248	3, 4	1	2	>3
	Rhodophyta	898	3-5	0	2	>3
	Angiospermae	44	4	0	2	>3
	Other Plantae	5	3, 4	ND	2	>3
Kingdom Protista (Protozoa)	Dinomastigota (Dinoflagellata)	470	3-5	0	4	>1
	Foraminifera	2,321	3-5	0	5	6
	Other Protista	1,410	1-5	0	16	>50
Kingdom Fungi		367	1-4	0	2	3
Kingdom Animalia	Porifera	745	1-5	0	1	14
	Cnidaria	1,876	1-5	1	16	>10
	Platyhelminthes	188	1-5	0	2	1
	Mollusca	8,658	1-5	11	10	>10
	Annelida	1,076	1-5	10	7	4
	Crustacea	6,232	2-5	10	>20	>10
	Bryozoa	300	5	0	2	>1
	Echinodermata	1,052	3-5	0	6	2
	Urochordata (Tunicata)	384	4, 5	2	4	>3
	Other invertebrates	1,314	1-5	2	>10	>10
	Vertebrata (Pisces)	3,790	3-5	1	15	>50
	Other vertebrates	152	3-5	0	4	>50
	Sub-Total Eukarya	32,777		39		
	Total Regional Diversity[4]	33,629		39		

[1] 出現種数.
[2] 研究進捗レベル: 方法参照.
[3] 外来種数.
[4] 全ての分類群の詳細は Table S3.

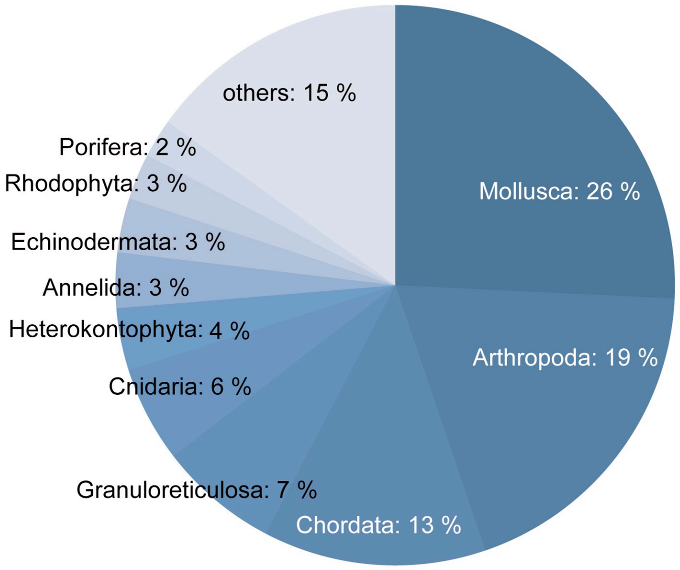

Figure 6. 生物門毎の日本近海出現種数（NDS）の割合．日本近海の海洋生物総種数（tNDS）に対するNDSが高い上位10位で全体の85%を占める．

固有種数（NES）の総数は，少なくとも1872となった（Table S5）．3つの綱，有孔虫綱Foraminifera，条鰭綱Actinopterygii，腹足綱GastropodaのNESはそれぞれ383，358，286種と高く，2つの目，アミ目Mysidaとヤギ目Gorgonaceaも高いNESを示し，NDSに対するNESの割合（PES）も約50%と高く，これらの分類群には固有種が多い傾向が認められた．節足動物門のプラティコピア目Platycopioida，胴甲動物門Loricifera，類線形動物門Nematomorphaからは1-2種しか出現しなかったが，それらは全て日本の固有種となった．ハプト植物門Haptophytaと環形動物門AnnelidaのサシバゴカイPhyllodocidaは，比較的高いNDSとなったにもかかわらず，NESは低く固有種が少ない傾向にあった．

今後出現が記録される種数（NUS）と推定種数（ENS）の総数は，それぞれ121913と155542となり（Table S4），このENS数155542が，本研究で評価した日本近海に生息する推定総種数となった．線虫動物門NematodaのNDSは70にもかかわらずNUSは115010にもなり（Table S4），日本近海の線虫動物門の種はほとんどが未記載種であった．比較的分類学研究が進んでいる脊索動物門，軟体動物門，節足動物門にも多数の未記載種が認められ（Tables S3，S4），例えば腹足綱の後鰓類Nudibranchia，節足動物門の端脚目Amphipodaや等脚目Isopoda，脊索動物門のハゼ科Gobiidaeには，200以上の未記載種が含まれていた．大型種を多く含む分類群でも，低次分類群になると研究の進捗レベルは様々であった．

日本近海における外来種数（NIS）の総数は39で，軟体動物門から10，環形動物門と節足動物門からそれぞれ10，脊索動物門から3，粘液胞子虫門Myxozoaから2，緑色植物門Chlorophyta，刺胞動物門Cnidaria，不等毛植物門Heterokontophytaからそれぞれ1種認められた（Table S6）．これら外来種の主な移入メカニズムは，船舶への付着やバラスト水への混入，輸入された水産物への混入であった．他方，日本ベントス学会によると40種以上の日本近海種が，他国の海域に外来種として侵入している．

研究進捗レベル State of knowledge

12の門（鉤頭動物門Acanthocephala，アメーバ動物門Amoebozoa，コウマクノウキン門Blastocladiomycota，ツボカビ門Chytridiomycota，有輪動物門Cycliophora，グロムス菌門Glomeromycota，太陽虫門Heliozoa，微胞子虫門Microsporidia，卵菌門Oomycota，オパリナ門Opalozoa，ペルコロゾア門Percolozoa，タウムアーキオータ門Thaumarchaeota）は，情報が得られず不明な分類群（Status 1）とした（Table S3）．これら不明な分類群の多くは，寄生性で真菌やいわゆる原生生物に属しているものであった．食用種や大型種を多く含む分類群は研究の進捗レベルが高くなったが，海綿動物門Porifera，刺胞動物門，軟体動物門，環形動物門といった比較的目に付きやすい分類群の中にも，低次分類群では研究の進捗レベルが高くないものが認められた（Tables S3, S4）．

OBIS に収録されている情報と NDS の比較 Comparisons between NDS and the number of species recorded from Japanese waters in OBIS

NDSがOBIS内にどれくらい収録されているかを解析するために求めた日本近海各分類群のOBIS内種数（NDSo）の割合（PRO）は，3つの門，線形動物門，箒虫動物門Phoronida，鰓曳動物門Priapulidaでほぼ100 %となった（Table 5）．これらの門においては，NDSとNDSoがほぼ同じことをであるが，学名を確認していないため同じ種を含んだ数かどうかは不明である．節足動物門，脊索動物門，棘皮動物門Echinodermata，不等毛植物門，軟体動物門のように高いNDSを示した門のPROは低い値となった．環形動物門のPROは中庸な値であった．日本近海の海洋生物総種数（tNDS） は33629になるが，OBIS内種数（NDSo）の総数はわずか2820であった．

Table 5. 日本のEEZ内から記録されている各分類群のOBIS内種数（NDSo）と出現種数（NDS）に対するNDSoの割合（PRO）．

**Phylum/Division**	**NDS[1]**	**NDSo**	**PRO (%)**
Nematoda	70	71	101
Phoronida	2	2	100
Priapulida	2	2	100
Cryptophyta	8	5	63
Annelida	1,076	529	49
Dinomastigota	470	187	40
Sipuncula	47	17	36
Ectoprocta/Bryozoa	300	85	28
Cyanobacteria	11	2	18
Hemichordata	11	2	18
Chlorophyta	248	42	17
Heterokontophyta	1,207	191	16
Arthropoda	6,393	663	10
Cnidaria	1,860	181	10
Echiura	21	2	10
Echinodermata	1,052	97	9
Chaetognatha	36	3	8
Chordata	4,330	242	6
Brachiopoda	73	4	5
Mollusca	8,658	415	5
Rhodophyta	898	39	4
Ctenophora	41	1	2
Ciliophora	530	12	2
Porifera	745	12	2
Haptophyta	304	3	1
Granuloreticulosa	2,321	11	0
	Total	2,820	

[1] 出現種数

考察 Discussion

OBISによると，現在記載されている地球上の全海洋生物種数は約230000種になる．日本近海の海洋生物総種数（tNDS） 33629は（Table 4），地球上の全海洋生物種数の 14.6%になる．日本の領海とEEZをあわせた近海面積は4.47 x $10^6 km^2$で，これは全海洋の面積360 x $10^6 km^2$の1.2%にすぎない．また，近海容積は12 x $10^6 km^3$で，これは全海洋の1370 x $10^6 km^3$の0.9%にすぎない．つまり，日本近海は全海洋のなかで占める面積

や容積は小さいが，種の多様性は高いことがわかる．日本近海が高い種多様性を持つのは，地形，地質，物理，化学的に多様な環境があることに起因すると思われる [19，"日本近海の概要" 参照]．他国にくらべ日本では多くの海洋生物を食料として利用してきたことから，海洋生物多様性や分布データが多数蓄積されているため，種多様性は高くなることも一因であろう．いずれにせよ，日本近海の種多様性は他の海域に比べ高いことは明確である．

2002年に日本分類学会連合は，日本の陸上・海洋における日本産生物種数調査を行った（http://research2.kahaku.go.jp/ujssb/search）．その評価は，日本の陸上と海洋をあわせた生物総種数は約90000としている[20]．昆虫綱が最も高い種多様性を示し，種数は約30000種と日本の全種数の1/3に達する．本研究で得られた日本近海の海洋生物総種数（33629） もまた日本の全種数の1/3に達する．日本の陸の面積3.78×10^5km²と近海（EEZと領海）面積4.47×10^6km²の比は約1:12である．単位面積1km²あたりの種数を評価すると，海は7.5×10^{-3}種/km²で陸の2.4種/km²に比して著しく低いが，門の数は海の方が多い．海洋生物を対象にした分類学や生態学研究は，調査しにくいため陸上に比べると情報が少ない．特に深海域の情報が少なく，例えば深海の熱水噴出孔生物群集からは，ここ30年間で500を超える新種が記載されている [21]．これは，陸上に比べ海洋には未記載種が多数存在し，種多様性が過小評価されていることを示唆している．つまり，今後，研究の進展に伴い海洋における種の多様性はさらに高くなることが予想される．

1981年に西村（1981）によって，日本近海の代表的な分類群について，出現種数（NDS）が評価されている [19]． それと28年後の本研究の値を比較すると，端脚目Amphipoda，ヒトデ目Asteroidea，頭足綱Cephalopoda，ヒドロ虫綱Hydrozoa，魚類Pisces，多板綱Polyplacophora，ウミグモ綱Pycnogonidaにおいて，分類学や生態学研究が進んだことでNDSは増加している（Table 6）．石灰海綿綱Calcarea，ウニ綱Echinoidea，鉢虫綱Scyphozoa，星口動物門SipunculaのNDSは1981年時と同じか少なくなっている．最近，石灰海綿綱，ウニ綱，鉢虫綱の分類学研究が日本で活動的になってきたため，近い将来これらのNDS値は増加すると思われる．しかし，星口動物門を専門とする研究者は1名しか見あたらない．

Table 6. いくつかの分類群における本研究と西村（1981）[19]のNDSの差.

分類群			**西村(1981) における NDS [19]**	**本研究の NDS**	**NDS の増加数 [2]**
門	綱	目			
Chordata	Pisces		2700	3790	1090
Cnidaria	Hydrozoa		315	523	208
Chordata	Ascidiacea		281	313	32
Echinodermata	Ophiuroidea		ca. 260	308	48
Echinodermata	Echinoidea		192	161	-31
Echinodermata	Asteroidea		167	280	113
Platyhelminthes	Polycladida	Polycladida	149	150	1
Porifera	Calcarea		130	130	0
Mollusca	Cephalopoda		125	204	79
Arthropoda	Pycnogonida		67	153	86
Sipuncula			58	47	-11
Arthropoda	Crustacea	Amphipoda	57	544	487
Mollusca	Polyplacophora		56	129	73
Brachiopoda			55	73	18
Arthropoda	Crustacea	Stomatopoda	41	56	15
Cnidaria	Scyphozoa		38	37	-1
Echiura			17	21	4

[1] 出現種数
[2] 本研究と西村（1981）[19] のNDSの差

日本近海の固有種数（NES）は日本の海洋生物総種数（tNDS）の5.6％でそれほど大きな値ではない（Table S5）．海洋生物は生活史の一時期に浮遊期もしくは漂泳期を持つものが多く，固有種数は一般に多

くはならない．ただし特殊な生息域，洞窟，熱水噴出域，メタン湧出域，鯨死骸域，沈木堆積域などでは多くの固有種が報告されている [7, 22, 23]．日本近海には大きな海流があり（Figure 3），それらは海洋生物の分散を促している．例えば，黒潮は赤道域付近の種を，親潮は北東太平洋の種を日本近海に移送する [2]．古生物学的な研究から，日本近海の十脚目Decapodaやウニ綱の種は固有性が高いと言われている [19]．しかし，本研究の十脚目におけるNDSに対するNESの割合（PES）は1.1%と低く，古生物学的な研究の結果とは一致しない．

今後出現が記録される種数（NUS）は93門のうち30門で評価できた（Table S4）． 日本近海におけるNUSの総計は121913となったが，人との係わりが少ない分類群や微小な種ではNUSの評価できなかったため，このNUSは過小評価値である． NUSの総計はtNDSの約4倍となっている．線虫動物門Nematodaでは，未記載種を含めると特に高い種多様性が指摘されている [24, 25]． 本研究においても，日本近海の線虫動物門のNUSは極めて高い値を示している．このような高いNUSにもかかわらず，日本に線虫動物門を専門とする分類学研究者はわずかである．NDSよりNUSが小さい分類群は，比較的分類学研究が進んでいると思われるが，例えば，環形動物門Annelida，節足動物門Arthropoda，脊索動物門Chordata，刺胞動物門Cnidaria，顆粒根足虫門Granuloreticulosa，軟体動物門Mollusca，放射虫門Radiozoaの中にも適した分類形質がないために多くの隠蔽種が含まれる可能性もある．輪形動物門Rotifera，ケルコゾア門Cercozoa，脊索動物門，シアノバクテリア門CyanobacteriaのNDSに対するNUSの割合は，それぞれ0.3%, 2.8%, 7.5%, 9.1%と低く研究進捗レベルは高いように見える．これらのうち，ケルコゾア門やシアノバクテリア門に含まれる生物種のサンプルは十分ではなく分類学研究が未着手な種も多数あるため過小評価と思われる．

魚類を含む脊索動物門は，日本近海で最も分類学研究が良く行われている分類群であるが，その中にも多数の未記載種が含まれている．特に，ハゼ科Gobiidae魚類は316の既知種に対し216の未記載種が認められる．ハゼ科魚類に未記載種が多い原因は，（1）サンプル採集が難しいこと，（2）種同定を行うための適した形質が少ないこと，（3）分類学研究のための研究予算が少ないことが挙げられている [26]．これらの原因は，NDSに対してNUSが高い（研究進捗レベルが低い分類群）に共通する．一方で，分類学研究が積極的に行われた結果，NDSに対してNUSが高くなった場合もある．例えば，1999年以前に28種のクダクラゲ類が日本近海から記載されていたが [27]，これらの多くは最新の分類学体系に合致しないものであった．1999年以降，分類学的再検討が行われ，日本近海から少なくとも65種のクダクラゲ類が出現し [28, 29]，少なくとも9種が未記載種となり中には属や科の新設が必要なものも認められる [28]．

船舶による貿易が増えることに伴い，世界的に外来種の問題が大きくなってきている．外来種は，在来生態系，水産業，船舶の運航，発電所に深刻な影響をもたらしている [30-33]．日本近海からは，少なくとも39種の外来種が認められたが（Table S6），実際例として，腹足綱のカラムシロ*Nassarius sinarus* は水産業に影響し [33]，サキグロタマツメタ *Euspira fortunei* は二枚貝綱の在来アサリ*Ruditapes philippinarum* への食害が報告されている [34]．軟体動物門のイガイダマシ *Mytilopsis sallei*，ムラサキイガイ*Mytilus galloprovincialis,* ミドリイガイPerna viridis，環形動物門のカサネカンザシゴカイ *Hydroides elegans*，ナデシコカンザシゴカイ*Hydroides dianthus*，節足動物門のタテジマフジツボ*Balanus amphitrite,* アメリカフジツボ*Balanus eburneus*は，水産養殖に被害を与えたり，船体や発電所の配水管への付着によって大きな影響を与えている．タテジマフジツボ，キタアメリカフジツボ*Balanus glandula*，チチュウカイミドリガニ*Carcinus aestuarii*，ムラサキイガイは，侵入先で在来種を駆逐している．緑色植物門のイチイヅタ*Caulerpa taxifolia*は“Killer Algae”と呼ばれ，オーストラリア沿岸のインド洋から地中海やアメリカ沿岸に移入され，在来海洋生態系に大きな影響を及ぼしているが，本種は最近日本近海でも出現が確認されていた．大型船舶のバラスト水への混入や船体付着による搬送は，主要な外来種の分散メカニズムとなっている．日本は世界有数の貿易国で，日本から持ち出されるバラスト水は世界の船舶が運ぶバラスト水の約10%を占める [32]．これは，日本から他の海域へ外来種として生物を分散させる可能性が高いことを意味する．そのようなことを少しでも避けるために，日本では2004年に特定外来生物による生態系等に係る被害の防止に関する法律（特定外来生物法）が制定されている．

研究の進捗レベル State of knowledge

人との関わりが深く比較的大型の種を多く含む分類群では，分類学・生態学的研究が進んでいる傾向にあるが，日本近海にも情報が得られず不明な分類群（Status 1）が含まれるものが多数ある（Table S3）．それらは，鉤頭動物門Acanthocephala，アメーバ動物門Amoebozoa，アピコンプレクサ門Apicomplexa，有輪動物門Cycliophora，太陽虫門Heliozoa，卵菌門Oomycota，オパリナ門Opalozoa，ペルコロゾア門Percolozoaで，鉤頭動物門を除けば，いずれも小型種からなる生物門である．これらの生物群は，小型であるため採集が難しい，分類学的形質が少ない，日本に専門研究者が少ない（世界的にも少ない）ため研究が進まない．最近の分子生物学や顕微鏡技術の発展は，分類学的形質が少ないことを補うことが期待されている．

近年，分子系統学研究の進展により，真核生物の高次分類群は，6つのスーパーグループに分類される傾向にある［35, 36］（Figure 7）．例えばアメーバ動物Amoebozoaはスーパーグループの1つとなるが，太陽虫門はどのスーパーグループに属するのかわかっていない．それぞれのスーパーグループは，いわゆる原生生物と呼ばれる小型種を多数含み，原生生物は多様な分類群にわたっていることが明らかになってきている[36]．したがって，真核生物の多様性や進化を理解するためには，これら小型生物種の系統分類を進めることが重要となる．

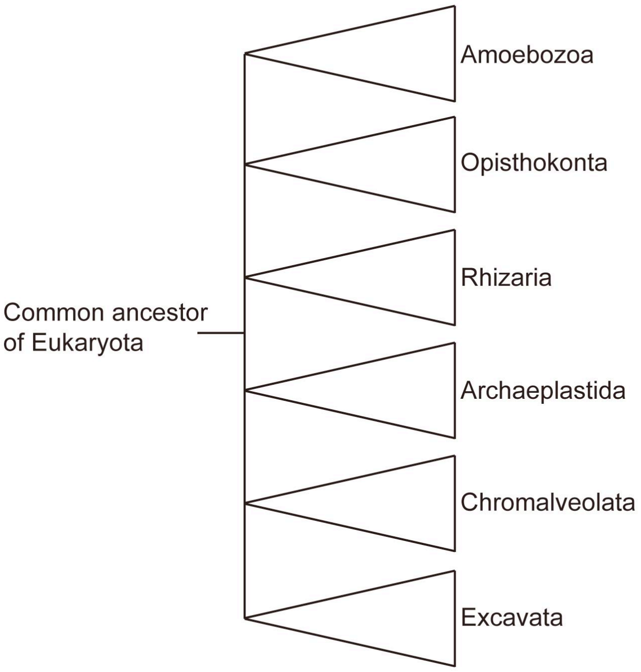

Figure 7. 分子系統解析による真核生物の6つのスーパーグループ．瀧下（2008）を改編［36].

本研究により，日本近海には分類学・生態学の側面から見て，良く研究が進んでいる分類群からほとんど情報がないものまであることがわかった．研究の進捗レベルをよりわかりやすくするため，門レベルで以下の定義によりKnown, Mostly unknown, Unknownの3つに分けた．

- Known：多くの上目，下目，亜目もしくは科において研究の進捗レベルが4か5にあるもの．
- Mostly unknown：KnownやUnknownに入らないもの．
- Unknown：多くの上目，下目，亜目もしくは科において研究の進捗レベルが1にあるもの．

そして，Known は22門，Mostly unknown は42門，Unknown は14門になった（Table 7）．つまり，比較的海洋生物研究が進んでいると思われた日本においても，全体的に見ると，分類学・生態学研究のデータが少ないことが示唆された．今後は，とりわけデータが少ない分類群の分類学・生態学研究者の育成が重要である．

Table 7. 門毎の分類学研究の進捗レベル（Known, Mostly unknown, Unknown）.

分類学研究の進捗レベル			
Known	Mostly unknown		Unknown
Acoelomorpha	Acidobacteria	Hemichordata	Acanthocephala
Bacteroides	Actinobacteria	Heterokontophyta	Amoebozoa
Cercozoa	Annelida	Kinorhyncha	Apicomplexa
Chaetognatha	Aquificae	Loricifera	Blastocladiomycota
Chlorophyta	Arthropoda	Metamonada	Chytridiomycota
Choanozoa	Ascomycota	Nematoda	Cycliophora
Chordata	Basidiomycota	Nematomorpha	Glomeromycota
Ctenophora	Brachiopoda	Nemertea	Heliozoa
Cyanobacteria	Ciliophora	Nitrospirae	Microsporidia
Dicyemida	Cnidaria	Orthonecta	Oomycota
Echinodermata	Crenarchaeota	Phoronida	Opalozoa
Ectoprocta/Bryozoa	Cryptophyta	Placozoa	Percolozoa
Firmicutes	Deferribacteres	Platyhelminthes	Priapulida
Granuloreticulosa	Deinococci	Porifera	Thaumarchaeota
Haptophyta	Dinomastigota	Proteobacteria	
Labyrinthulomycota	Echiura	Sipuncula	
Magnoliopsida	Entoprocta	Tardigrade	
Mollusca	Euglenophyta	Thermotogae	
Myxozoa	Euryarchaeota	Verrucomicrobia	
Radiozoa	Gastrotricha	Zygomycota	
Rhodophyta	Glaucophyta		
Rotifera	Gnathostomulida		

日本近海の海洋生物データベース Databases concerning marine life in Japan

OBIS は，海洋生物の生物地理学，生態学，分類学，変動予測などを研究する上で強力なツールである．しかし，日本近海各分類群のOBIS内種数（NDSo）の割合（PRO）はわずか8.4%と低い．日本にも海洋生物の多様性や分布を扱うデータベースはあり，それらのデータとOBISを連携することで，日本を含む全海洋生物研究の進展に貢献できると思われる．以下に日本のデータベースのいくつか示す．

- Algae resource database: http://www.shigen.nig.ac.jp/algae/
- 北海道海鳥センター: http://www3.town.haboro.hokkaido.jp/seabird/
- Biological Information System for Marine Life （BISMaL）: http://www.godac.jp/bismal/searchSpecies.jsf
- CMarZ-Asia Database: http://cmarz-asia.org/db/
- 魚類写真資料データベース: http://research.kahaku.go.jp/zoology/photoDB/
- 海棲哺乳類情報データベース: http://svrsh1.kahaku.go.jp/m/mm/
- Japan Collection of Microorganisms: http://www.jcm.riken.jp/
- 日本産生物種数調査: http://research2.kahaku.go.jp/ujssb/search
- 日本海洋データセンター: http://www.jodc.go.jp/index_j.html
- NaGISA Database: http://www.nagisa.coml.org/
- NITE Biological Resource Center: http://www.nbrc.nite.go.jp/
- 日本の海藻百選: http://research.kahaku.go.jp/botany/seaweeds/JS100Home.html
- 原生生物情報サーバ: http://protist.i.hosei.ac.jp/protist_menu.html
- 日本爬虫類標準和名: http://mail2.nara-edu.ac.jp/~inoue/NNM/hatyuurui/wamei-h.html
- 日本産クマムシ類: http://homepage3.nifty.com/cxj11255/jtard/index.html

これらのうち， NaGISA Database, CMarZ-Asia Database, 日本海洋データセンターのデータは直接もしくは間接的にOBISに反映されている．BISMaLもOBISやGlobal Biodiversity Information Facility （GBIF） にデー

タを反映できるように国際標準フォーマットであるダーウインコアスキーマでのデータを装備している．国内データベースの多くは，日本語やダーウインコアスキーマに準じていないことが多く，容易にOBISにデータを反映することができない．このような問題を解決するために，早急にOBISの日本ノードを整備する必要がある．

日本近海の生物多様性や生態系は，（1）天然水産資源の減少，（2）水産養殖の増加，（3）特定漁獲種の変化，（4）漁業海域の変化，（5）食物網の変化，（6）個体群・種・遺伝子レベルでの多様性の変化，（7）種や個体群の絶滅，（8）分布域の縮小拡大といった変化，（9）生物分散に関わる輸送様式の変化，（10）外来種の侵入，（11）栄養循環の変化，（12）表層における生物生産や海底への炭素フラックスの変化などに関し変動することが予想される．しかし，我々は海洋生態系のサービスや機能を十分理解するための基礎データを十分に得ていない．日本近海でも，深海域のように未調査領域がたくさん残っている．日本は，海洋国家であり海洋生物の調査研究を行うには適した地理環境にある．とりわけ，日本は海溝やトラフといった深海環境にも囲まれ，深海生物研究のための有人潜水調査船，無人探査機，自律型無人探査機といったファシリティも運用している．2007年からは，統合国際深海掘削計画（IODP）のもとに，地球深部探査船「ちきゅう」が活動をはじまった．IODPの目的の1つは，海底下の生物圏探査であり，海底下の生物多様性や生態系の研究は未着手領域である．このように，日本は海洋生物研究を推進する環境にあり，今後海洋生物の理解のために積極的に研究を行う必要がある．

謝辞 Acknowledgments

本研究はTable S2に挙げた多数の研究者の方からの情報を基に行った．また，「海洋生物のセンサス」から多大な支援があり，特にMichele DuRand博士, Patricia Miloslavich博士, Dale Langford博士, Charles Griffiths博士には有益な助言をいただいた．古島靖夫博士には作図を助けていただき，丸山正博士には多岐にわたる支援をいただいた．これらの方々に心より感謝する．

引用文献 References

1. Costello MJ, Bouchet P, Emblow CS, Legakis A（2006）European marine biodiversity inventory and taxonomic resources: state of the art and gaps in knowledge. Mar Eco Prog Ser 316: 257-268.
2. 沖山宗雄・鈴木克美 （1985）日本の海洋生物．東京：東海大学出版会. 160 p.
3. 日本海洋学会沿岸海洋研究部会（1985）日本全国沿岸海洋誌．東京，東海大学出版会. 1106 p.
4. 米倉 伸之・野上 道男・貝塚 爽平・ 鎮西 清高（2001）日本の地形〈1〉総説．東京：東京大学出版会. 376 p.
5. Su JL, Guan BX, Jiang JZ （1990）The Kuroshio.Part I. Physical Features. Oceanogr Mar Biol Annu Rev 28: 11-71.
6. Kojima S （2002）Deep-sea chemoautosynthesis-based communities in the Northwestern Pacific. J Oceanogr 58: 343-363.
7. 藤倉克則・奥谷喬司・丸山正（2008） 潜水調査船が観た深海生物．秦野：東海大学出版会. 487 p.
8. 馬場菊太郎（1949）相模湾産後鰓類図譜.東京：岩波書店. 194 p.
9. 時岡隆（1953）相模湾産海鞘類図譜．東京：岩波書店. 315 p.
10. 酒井恒（1965）相模湾産蟹類．東京：丸善. 206 p.
11. 江口元起（1968）相模湾産ヒドロ珊瑚類および石珊瑚類．東京：丸善. 197 p.
12. 黒田徳米・波部忠重・大山桂（1971） 相模湾産貝類．東京：丸善. 489 p.
13. 林良二（1973）相模湾産海星類．東京：保育社. 239 p.
14. 三宅貞祥（1978）相模湾産甲殻異尾類．東京：保育社. 200 p.
15. 入村精一（1982）相模湾産蛇尾類．東京：丸善. 95 p.
16. 重井陸夫（1986）相模湾産海胆類．東京：丸善. 204 p.
17. 中村光一郎（1987）相模湾産海蜘蛛類．東京：丸善. 43 p.
18. 谷田専治（1989）相模湾産尋常海綿類．東京：丸善. 197 p.
19. 西村三郎（1981）地球の海と生命．東京：海鳴社. 284 p.
20. 椛原宏（2004）日本分類学会連合による日本産生物種数調査．生物科学 55: 71-77.
21. Desbruyéres D, Segonzac M, Bright M（2006）Handbook of deep-sea hydrothermal vent fauna. Linz-Dornach: Densia 18, 544 p.
22. Van Dover CL（2000）The ecology of deep-sea hydrothermal vents. Princeton: Princeton University Press, 424 p.

23. Hayami I, Kase T（1996）Characteristics of submarine cave bivalves in the northwestern Pacific. Am Malac Bull 12: 59-65.
24. Lambshead PJD（1993）Recent developments in marine benthic biodiversity research. Oceanus 19: 5-24
25. Lambshead PJD, Boucher G（2003）Marine nematode deep-sea biodiversity - Hyperdiverse or hype? Jour Biogeogr 30: 475-485.
26. 松浦啓一・瀬能宏（2004）日本に魚は何種いるのか —既知種と未知種をめぐる問題—. 生物科学 55: 79-86.
27. 千原光雄・村野正昭（1997）日本産海洋プランクトン検索図説. 東京：東海大学出版会. 1574 p.
28. ドゥーグル リンズィー・三宅裕志（2009）日本近海に出現する中・深層性刺胞動物ならびに有櫛動物の目録. 月刊海洋 53: 417-438.
29. Kitamura M, Tanaka Y, Ishimaru T（2003）Coarse scale distribution and community structure of hydromedusae related to water mass structure in two locations of Japanese waters in early summer. Plankton Biol Ecol 50: 43-54.
30. 西栄二郎・加藤哲哉（2004）環形動物多毛類の移入と移出の現状.日本ベントス学会誌　59: 83-95.
31. 岩崎敬二・木村妙子・木下今日子・山口寿之・西川輝昭ほか（2004）日本における海産生物の人為的移入と分散：日本ベントス学会自然保全委員会によるアンケート調査の結果から.日本ベントス学会誌 59: 22-44.
32. 大谷道夫（2004）日本の海洋移入生物とその移入過程について.日本ベントス学会誌　59: 45-57.
33. 福田宏（2004）外来種と同定の問題.日本ベントス学会誌　59: 68-73.
34. 大越健嗣（2004）移入アサリに混入して移入する生物—食害生物サキグロタマツメタと非意図的移入種. 日本ベントス学会誌　59: 74-82.
35. Adl SM, Simpson AGB, Farmer MA, Andersen RA, Anderson OR et al.（2005）The new higher level classification of eukaryotes with emphasis on the taxonomy of protists. J Eukaryot Microbio 52: 367-376.
36. 瀧下清貴（2008）化学合成生態系における深海微生物−真核生物の本当の多様性−. In: 藤倉克則・奥谷喬司・丸山正　編，潜水調査船が観た深海生物. 秦野：東海大学出版会. pp. 204-206.

Table S1. 日本における海洋生物研究史概要.

Year	Item	Reference
1709	Ekiken Kaibara (Japan): Published as a Yamato Honzo (concerning Japanese fauna and flora)	http://www.ndl.go.jp/nature/varie/varie_2.html
1741	Gensen Kanda (Japan): Published as a Nitto-Gyofu, the first marine animal monographs in Japan	http://www.ndl.go.jp/nature/varie/varie_2.html
1833-1850	Wilhem De Haan (Nederland): Published as Fauna Japonica (Crustacea), 8 Volumes, from Rijksmuseum van Natuurlijke Historie	
1842-1850	Coenraad J. Temminck and Hermann Schlegel (Nederland): Published as Fauna Japonica (Pisces), 16 Volumes, from Rijksmuseum van Natuurlijke Historie	
1871	Establishment of a Museum in Yushima Seido as part of the observational facilities of the Ministry of Education Museum Division (Present NMNS[1])	
1873-1876	Franz Hilgendorf (German): Professor of natural history in Tokyo Medical School. Faunal study of Marine organisms	
1875	Oceanographic investigation by the HMS *Challenger* around Japan	
1877-1879	Edward S. Morse (USA): Professor of the University of Tokyo. Establishment of the first Marine Laboratory in Enoshima Island, Kanagawa in 1877	国立科学博物館（2007）相模湾動物誌. 秦野：東海大学出版会. 212 p.
1879-1881	Ludwig. H. P. Döderlein (German): Professor of natural history in The University of Tokyo Faculty of Medicine. Faunal study of Sagami Bay	国立科学博物館（2007）相模湾動物誌. 秦野：東海大学出版会. 212 p.
1886	Establishment of Misaki Marine Biological Station, The University of Tokyo, in Misaki, Kanagawa	
1888	Establishment of the Fisheries Training School (Present TUMSAT[2])	
1904-1905	Franz Doflein (German): Deep-sea biological investigation in Sagami Bay	国立科学博物館（2007）相模湾動物誌. 秦野：東海大学出版会. 212 p.
1922	Establishment of a Marine Biological Research Station of the Faculty of Science, Kyoto Imperial University (Present Seto Marine Biological Laboratory, Field Science Education and Research Center, Kyoto University)	
1922-1930	Fishery investigation in continental shelf around Japan by the Imperial Fisheries Institute	
1925	Establishment of a Biological Laboratory, Imperial Household. Twelve marine organism monographs in Sagami Bay	
1925	Start of investigations using Research Vessel Soyo-Maru by the Imperial Fisheries Institute	
1929	Start of investigations (mainly fisheries science) using human-occupied vehicle *Nishimura-shiki Mame Sensui-tei ichi-go*, maximum depth 200 m	
1929	The Fisheries Experimental Station of the Ministry of Agriculture and Forestry was established in Tokyo（Present NRIFS[3])	

1932	Establishment of the Japanese Society of Fisheries Science	
1941	Establishment of the Oceanographic Society of Japan	
1951-1958	Start of investigations using human-occupied vehicle *Kuroshio-go*, maximum depth 200 m. "Marine Snow" termed during this human-occupied vehicle investigation	
1962	Establishment of the ORI[4], The University of Tokyo	
1963	Research Vessel *Tansei-maru* was completed by ORI[4], The University of Tokyo	
1967	Research Vessel *Hakuho-maru* was completed by ORI[4], The University of Tokyo	
1971	Establishment of the Japan Marine Science and Technology Center (JAMSTEC[5], present Japan Agency for Marine-Earth Science and Technology)	
1973-	The National Survey on the Natural Environment by the Ministry of the Environment	
1981	Human-occupied vehicle *Shinkai 2000* was completed by JAMSTEC[5]	
1984	Discovery of deep-sea chemosynthesis-based community in Sagami Bay. This was the first discovery of chemosynthesis-based community around Japan	
1986-	Investigation of endangered species in Japan by the Ministry of the Environment	
1989	Human-occupied vehicle *Shinkai 6500* was completed by JAMSTEC[5]	
1993	Conclusion of Convention on Biological Diversity	
2001	Establishment of Global Biodiversity Information Facility Japan	
2002	Japanese Biota Species Number Survey by Union of Japanese Societies for Systematic Biology	
2007	Establishment of the law "Basic Act on Ocean Policy"	

[1, 2, 3, 4, 5] 略語説明は Table 2 参照.

Table S2. 本研究への協力研究者リスト.

分類群		協力者 (研究機関[1])
Archaea		山本啓之 (JAMSTEC)
Bacteria		能木裕一 (JAMSTEC), 山本啓之 (JAMSTEC)
Eukarya	Acoelomorpha	田近謙一（日本大学）
	Ascomycota	長濱統彦 (JAMSTEC)
	Annelida	西栄二郎 (YNU)
	Arthropoda	福岡弘紀 (SNF), Mark J. Grygier (LBM), 岩崎望（高知大学），駒井智幸 (NHMIC), 長澤和也（広島大学），中村光一郎, 大塚攻（広島大学），遠部卓 （広島大学），齋藤暢宏（水土舎），澤本彰三（東海大学）,下村通誉 (KMNH), 上田拓史（高知大学），山口寿之(千葉大学)
	Basidiomycota	長濱統彦 (JAMSTEC)
	Brachiopoda	柁原宏（北海道大学）
	Cercozoa	鈴木紀毅（東北大学）
	Chaetognatha	後藤太一郎（三重大学），黒田一紀 (JSNFRI)
	Chlorophyta	田中次郎 (TUMSAT), 鈴木秀和 (TUMSAT)
	Chordata	甲能　直樹 (NMNS), 松浦啓一 (NMNS), 村山司 （東海大学），西川淳（東京大学），志賀直信 (HJC)
	Ciliophora	中町美和 (TNFRI)
	Cnidaria	今原幸光 (BIK), 久保田信 (Kyoto University), 野村恵一 (KMPC), 立川浩之 (NHMIC), 柳研介 (NHMIC)
	Ctenophora	堀田拓史, 峯水亮 (峯水写真事務所)
	Dicyemida	古屋秀隆（大阪大学）
	Dinophyta	田中次郎 (TUMSAT), 鈴木秀和 (TUMSAT)
	Echinodermata	藤田敏彦 (NMNS)
	Ectoprocta	柁原宏（北海道大学）
	Entoprocta	伊勢戸徹 (JAMSTEC)
	Glaucophyta	田中次郎 (TUMSAT), 鈴木秀和 (TUMSAT)
	Granuloreticulosa	長谷川四郎 (熊本大学)
	Haptophyta	河地正伸 (NIES)
	Magnoliopsida	田中次郎 (TUMSAT), 鈴木秀和 (TUMSAT)
	Metamonada	雪吹直史 (UBC)
	Mollusca	奥谷喬司 (JAMSTEC), 齋藤寛 (NMNS)
	Myxozoa	横山博（東京大学）
	Nemertea	柁原宏（北海道大学）
	Ochrophyta	田中次郎 (TUMSAT), 鈴木秀和 (TUMSAT)
	Oomycota	稲葉重樹 (NITE)
	Orthonecta	古屋秀隆（大阪大学）
	Phoronida	柁原宏（北海道大学）
	Placozoa	柁原宏（北海道大学）
	Platyhelminthes	田近謙一（日本大学）
	Porifera	伊勢優史（東京大学）
	Radiozoa	鈴木紀毅（東北大学）
	Rhodophyta	田中次郎 (TUMSAT), 鈴木秀和 (TUMSAT)
	Streptophyta	田中次郎 (TUMSAT), 鈴木秀和 (TUMSAT)

[1] 略語説明は Table 2 参照.

Table S3. 分類階級目レベルの日本近海における海洋生物の出現種数（NDS），研究進捗レベル，固有種数（NES），今後出現が記録される種数（NUS），推定種数（ENS），外来種数（NIS），分類学研究者数，種同定に役立つ文献数．目レベルに分類することができない分類群については，上目，下目，亜目もしくは科レベルとした．

ドメイン/界/スーパーグループ	門	綱	目/上目/下目/亜目/科	NDS	研究進捗レベル	NES	NUS	ENS	NIS	分類学研究者数（氏名：所属 [1]）	種同定に役立つ文献数	引用
Archaea				9		ND	ND	9	0	10 (Yoichi Kamagata: AIST, Ken Takai: JAMSTEC et al.)	>10	http://www.jcm.riken.jp/, http://www.nbrc.nite.go.jp/e/index.html, http://ijs.sgmjournals.org, http://www.bacterio.cict.fr/
	Crenarchaeota			2	3	ND	ND	2	0			
	Euryarchaeota			7	3	ND	ND	7	0			
	Thaumarchaeota			ND	1	ND	ND	ND	0			
Bacteria				843		ND	1	844	0	15 (Shigeaki Harayama: NITE, Yuichi Nogi: JAMSTEC, et al.)	>10	http://www.jcm.riken.jp/, http://www.nbrc.nite.go.jp/e/index.html, http:// ijs. sgmjournals.org/, http://www.bacterio. cict.fr/, http:// www.ncbi.nlm.nih.gov/ Taxonomy/Browser/wwwtax.cgi?id=2, http://www.nbrp.jp/report/reportProject.jsp
	Acidobacteria			3		ND	ND	3	0			
		Acidobacteria		1		ND	ND	1	0			
			Acidobacteriales	1	3	ND	ND	1	0			
		Holophagae		2		ND	ND	2	0			
			Acanthopleuribacteraceae	1	3	ND	ND	1	0			
			Holophagales	1	3	ND	ND	1	0			
	Actinobacteria			123		ND	ND	123	0			
		Actinobacteria		123		ND	ND	123	0			
			Acidimicrobiale	1	3	ND	ND	1	0			
			Actinomycetales	121	5	ND	ND	121	0			
			Bifidobacteriales	1	3	ND	ND	1	0			
	Aquificae			2		ND	ND	2	0			
		Aquificae		2		ND	ND	2	0			
			Aquificales	2	3	ND	ND	2	0			
	Bacteroides			125		ND	ND	125	0			
		Bacteroidia		2		ND	ND	2	0			
			Bacteroidales	2	3	ND	ND	2	0			
		Flavobacteria		83		ND	ND	83	0			
			Flavobacteriales	83	4	ND	ND	83	0			
		Sphingobacteria		40		ND	ND	40	0			
			Sphingobacteriales	40	4	ND	ND	40	0			
	Cyanobacteria			11	5	ND	1	12	0			
	Deferribacteres			1		ND	ND	1	0			
		Deferribacteres		1		ND	ND	1	0			

			Deferribacterales	1	3	ND	ND	1	0			
	Deinococci			1		ND	ND	1	0			
		Deinococci		1		ND	ND	1	0			
			Deinococcales	1	3	ND	ND	1	0			
	Firmicutes			172		ND	ND	172	0			
		Bacilli		172		ND	ND	172	0			
			Bacillales	159	5	ND	ND	159	0			
			Lactobacillales	13	4	ND	ND	13	0			
	Nitrospirae			2		ND	ND	2	0			
		Nitrospira		2		ND	ND	2	0			
			Nitrospirales	2	3	ND	ND	2	0			
	Proteobacteria			395		ND	ND	395	0			
		Alphaproteobacteria		78		ND	ND	78	0			
			Caulobacterales	4	3	ND	ND	4	0			
			Rhizobiales	18	4	ND	ND	18	0			
			Rhodobacterales	41	4	ND	ND	41	0			
			Sphingomonadales	15	4	ND	ND	15	0			
		Betaproteobacteria		4		ND	ND	4	0			
			Burkholderiales	2	3	ND	ND	2	0			
			Neisseriales	2	3	ND	ND	2	0			
		Deltaproteobacteria		7		ND	ND	7	0			
			Desulfobacterales	2	3	ND	ND	2	0			
			Desulfovibrionales	2	3	ND	ND	2	0			
			Desulfuromonadales	2	3	ND	ND	2	0			
			Myxococcales	1	3	ND	ND	1	0			
		Epsilonproteobacteria		10		ND	ND	10	0			
			Campylobacterales	4	3	ND	ND	4	0			
			Nautiliales	2	3	ND	ND	2	0			
			incertae sedis	4	3	ND	ND	4	0			
		Gammaproteobacteria		296		ND	ND	296	0			
			Aeromonadales	3	3	ND	ND	3	0			
			Alteromonadales	91	4	ND	ND	91	0			
			Chromatiales	2	3	ND	ND	2	0			
			Enterobacteriales	8	3	ND	ND	8	0			
			Legionellales	3	3	ND	ND	3	0			
			Oceanospirillales	53	4	ND	ND	53	0			
			Pseudomonadales	58	4	ND	ND	58	0			

			Thiotrichales	6	3	ND	ND	6	0			
			Vibrionales	63	4	ND	ND	63	0			
			Xanthomonadales	4	3	ND	ND	4	0			
			incertae sedis	5	3	ND	ND	5	0			
	Thermotogae			4		ND	ND	4	0			
		Thermotogae		4		ND	ND	4	0			
			Thermotogales	4	3	ND	ND	4	0			
	Verrucomicrobia			4		ND	ND	4	0			
		Verrucomicrobiae		4		ND	ND	4	0			
			Verrucomicrobiales	4	3	ND	ND	4	0			
Eukarya/Chromista				1,247		ND	ND	1,247	1	2 (Hidekazu Suzuki: TUMSAT, Jiro Tanaka: TUMSAT)		
	Heterokontophyta/Ochrophyta			1,207		ND	ND	1,207	1			
		Bacillariophyceae		862		ND	ND	862	ND		3	千原光雄・村野正昭（1997）日本産海洋プランクトン検索図説. 東京：東海大学出版会. 1574 p., 小林弘・出井雅彦・真山茂樹・南雲保・長田敬五（2006）小林弘珪藻図鑑 第 1 巻. 東京：内田老鶴圃. 596 p.
			Centrales	425	5	ND	ND	425	ND			
			Pennales	437	5	ND	ND	437	ND			
		Chrysomerophyceae		2		ND	ND	2	ND		>3	千原光雄・村野正昭（1997）日本産海洋プランクトン検索図説. 東京：東海大学出版会. 1574 p., 小林弘・出井雅彦・真山茂樹・南雲保・長田敬五（2006）小林弘珪藻図鑑 第 1 巻. 東京：内田老鶴圃. 596 p.
			Chrysomeridales	2	3	ND	ND	2	ND			
		Chrysophyceae		5		ND	ND	5	0			
			Chromulinales	5	3	ND	ND	5	0			
		Dictyochophyceae		9		ND	ND	9	ND			
			Ciliophryales	1	3	ND	ND	1	ND			
			Dictyochales	5	3	ND	ND	5	ND			
			Pedinellales	2	3	ND	ND	2	ND			
			incertae sedis	1	3	ND	ND	1	ND			
		Eustigmatophyceae		5		ND	ND	5	ND			
			Eustigmatales	5	3	ND	ND	5	ND			
		Pelagophyceae		3		ND	ND	3	ND			
			Sarinochrysidales	3	3	ND	ND	3	ND			
		Phaeophyceae/Phaeophyta		304		ND	ND	304	ND			
			Chordariales	54	4	ND	ND	54	ND			

			Cutleriales	3	3	ND	ND	3	ND			
			Desmarestiales	3	3	ND	ND	3	ND			
			Dictyosiphonales	25	4	ND	ND	25	ND			
			Dictyotales	39	4	ND	ND	39	ND			
			Ectocarpales	24	4	ND	ND	24	ND			
			Fucales	69	4	ND	ND	69	ND			
			Laminariales	39	4	ND	ND	39	ND			
			Ralfsiales	17	4	ND	ND	17	ND			
			Scytosiphonales	17	4	ND	ND	17	ND			
			Sphacelariales	10	4	ND	ND	10	ND			
			Sporochnales	3	3	ND	ND	3	ND			
			Syringodermatales	1	3	ND	ND	1	ND			
		Pinguiophyceae		1		ND	ND	1	ND			
			Pinguiochrysidales	1	3	ND	ND	1	ND			
		Raphidophyceae		15		ND	ND	15	ND			
			Raphidomonadales	15	4	ND	ND	15	ND			
		Xanthophyceae		1		ND	ND	1	1			
			Vaucheriales	1	3	ND	ND	1	1			
	Labyrinthulomycota			40	4	ND	ND	40	0	2	ND	杉山純多（2005）菌類・細菌・ウイルスの多様性と系統. 東京：裳華房. 492 p.
	Oomycota			ND	1	ND	ND	ND	ND	1 (Shigeki Inaba: NITE)		http://research2.kahaku.go.jp/ujssb/search
	Opalozoa			ND	1	ND	ND	ND	ND	ND		
Eukarya/Plantae				1,195		ND	ND	1,195	1	2 (Jiro Tanaka: TUMSAT, Hidekazu Suzuki: TUMSAT)	>3	http://research2.kahaku.go.jp/ujssb/search
	Angiospermae/Magnoliopsida			44		ND	ND	44	ND			
		Liliopsida		44		ND	ND	44	ND			
			Najadales	44	4	ND	ND	44	ND			
	Chlorophyta			248		ND	ND	248	1			
		Chlorophyceae		2		ND	ND	2	0			
			Chlamydomonadales	1	3	ND	ND	1	0			
			Chlorococcales	1	3	ND	ND	1	0			
		Ulvophyceae		246		ND	ND	246	1			
			Caulerpales	86	4	ND	ND	86	1			
			Cladophorales	86	4	ND	ND	86	0			
			Dasycladales	16	4	ND	ND	16	0			
			Ulotrichales	27	4	ND	ND	27	0			
			Ulvales	31	4	ND	ND	31	0			

	Glaucophyta			4		ND	ND	4	0			
		Glaucophyceae		4		ND	ND	4	0			
			Cyanophorales	3	3	ND	ND	3	0			
			Glaucocystales	1	3	ND	ND	1	0			
	Rhodophyta			898		ND	ND	898	0			
		Bangiophyceae		42		ND	ND	42	0			
			Bangiales	30	4	ND	ND	30	0			
			Compsopogonales	5	3	ND	ND	5	0			
			Erythropeltidales	7	3	ND	ND	7	0			
		Cyanidiophyceae		2		ND	ND	2	0			
			Cyanidiales	2	3	ND	ND	2	0			
		Florideophyceae		841		ND	ND	841	0			
			Acrochaetiales	37	4	ND	ND	37	0			
			Ahnfeltiales	1	3	ND	ND	1	0			
			Batrachospermales	27	4	ND	ND	27	0			
			Bonnemaisoniales	4	3	ND	ND	4	0			
			Ceramiales	320	5	ND	ND	320	0			
			Corallinales	106	5	ND	ND	106	0			
			Gelidiales	27	4	ND	ND	27	0			
			Gigartinales	127	5	ND	ND	127	0			
			Gracilariales	22	4	ND	ND	22	0			
			Halymeniales	38	4	ND	ND	38	0			
			Hildenbrandiales	3	3	ND	ND	3	0			
			Nemaliales	56	4	ND	ND	56	0			
			Nemastomatales	11	4	ND	ND	11	0			
			Palmales	11	4	ND	ND	11	0			
			Plocamiales	5	3	ND	ND	5	0			
			Rhodymeniales	43	4	ND	ND	43	0			
			Thoreales	3	3	ND	ND	3	0			
		Rhodellophyceae		13		ND	ND	13	0			
			Goniotrichales	8	3	ND	ND	8	0			
			Porphyridiales	5	3	ND	ND	5	0			
	incertae sedis			1		ND	ND	1	0			
		Mesostigmatophyceae		1		ND	ND	1	0			
			Mesostigmatales	1	3	ND	ND	1	0			
Eukarya/Protista				4,201		383	594	4,795	0			
Eukarya/Protista/Al veolata				1,000		ND	ND	1,000	0			

	Apicomplexa			ND		ND	ND	ND	0	ND	ND	
		Sporozoea		ND	1	ND	ND	ND	0			
	Ciliophora			530		ND	ND	530	0	4 (Miwa Nakamachi: TNFRI, Takashi Ota: ISU, et al.)	1	http://protist.i.hosei.ac.jp/protist_menu.html, http://www.jodc.go.jp/data/biology_j.html
		Heterotrichea		16		ND	ND	16	0			
			Heterotrichida	10	4	ND	ND	10	0			
			Licnophorida	1	3	ND	ND	1	0			
			Plagiotomida	1	3	ND	ND	1	0			
			Peritromida	4	3	ND	ND	4	0			
		Karyorelictea		6		ND	ND	6	0			
			Loxodida	2	3	ND	ND	2	0			
			Protostomatida	3	3	ND	ND	3	0			
			Protoheterotrichida	1	3	ND	ND	1	0			
		Litostomatea		14		ND	ND	14	0			
			Haptorida	8	3	ND	ND	8	0			
			Pleurostomatida	6	3	ND	ND	6	0			
		Nassophorea		15		ND	ND	15	0			
			Nassulida	7	3	ND	ND	7	0			
			Synhymeniida	8	3	ND	ND	8	0			
		Oligohymenophora		34		ND	ND	34	0			
			Hymenostomatida	3	3	ND	ND	3	0			
			Mobilida	2	3	ND	ND	2	0			
			Sessilida	7	3	ND	ND	7	0			
			Peniculida	6	3	ND	ND	6	0			
			Scuticocilatida	15	4	ND	ND	15	0			
			Plagiopylida	1	3	ND	ND	1	0			
		Phyllopharyngea		11		ND	ND	11	0			
			Cyrtophorida	11	4	ND	ND	11	0			
		Prostomatea		6		ND	ND	6	0			
			Prorodontida	6	3	ND	ND	6	0			
		Spirotrichea		428		ND	ND	428	0			
			Choreotrichida	349	5	ND	ND	349	0			
			Stichotrichida	6	3	ND	ND	6	0			
			Oligotrichida	63	4	ND	ND	63	0			
			Euplotida	9	3	ND	ND	9	0			
			Armophorida	1	3	ND	ND	1	0			
	Dinomastigota/Dinoflagellata/Dinophyta			470		ND	ND	470	0	4 (Jiro Tanaka: TUMSAT, Hidekazu Suzuki: TUMSAT, et	>1	千原光雄・村野正昭（1997）日本産海洋プランクトン検索図説．東京：東海

										al.)		大学出版会. 1574 p.
		Dinophyceae		461		ND	ND	461	0			
			Dinophysiales	57	4	ND	ND	57	0			
			Gonyaulacales	124	5	ND	ND	124	0			
			Gymnodiniales	86	4	ND	ND	86	0			
			Kolkwitziellales	1	3	ND	ND	1	0			
			Peridiniales	166	5	ND	ND	166	0			
			Phytodiniales	2	3	ND	ND	2	0			
			Prorocentrales	24	4	ND	ND	24	0			
			Thoracosphaerales	1	3	ND	ND	1	0			
		Noctilciphyceae		9		ND	ND	9	0			
			Noctilcales	9	3	ND	ND	9	0			
Eukarya/Protista/Amoebobiota				ND		ND	ND	ND	ND	ND	ND	
	Amoebozoa			ND	1	ND	ND	ND	ND			
Eukarya/Protista/Chromalveolata				312		0	ND	312	0			
	Cryptophyta			8		ND	ND	8	0	1 (Mayumi Erata: Global Environmental Forum)	>1	千原光雄・村野正昭（1997）日本産海洋プランクトン検索図説. 東京：東海大学出版会. 1574 p.
		Cryptophyceae		8		ND	ND	8	0			
			Cryptomonadales	8	3	ND	ND	8	0			
	Haptophyta			304		0	ND	304	0	5 (Masanobu Kawachi: NIES, Takeshi Nakayama: University of Tsukuba, et al.)	10	Jordan RW, Cros L, Young JR（2004）A revised classification scheme for living haptophytes. Micropaleontol 50: 55-79.
		Pavlovophyceae		11		0	ND	11	0			
			Pavlovales	11	4	0	ND	11	0			
		Prymnesiophyceae		293		0	ND	293	0			
			Coccolithales	26	4	0	ND	26	0			
			Isochrysidales	20	4	0	ND	20	0			
			Phaeocystidales	9	3	0	ND	9	0			
			Prymnesiales	71	4	0	ND	71	0			
			Syracosphaerales	99	4	0	ND	99	0			
			Zygodiscales	67	4	0	ND	67	0			
			incertae sedis	1	3	0	ND	1	0			
Eukarya/Protista/Excavata				20		ND	ND	20	0			
	Euglenophyta			14		ND	ND	14	0	1 (Minoru Saito: YNU)	1	千原光雄・村野正昭（1997）日本産海洋プランクトン検索図説. 東京：東海大学出版会. 1574 p.
		Euglenophyceae		14		ND	ND	14	0			
			Euglenales	8	3	ND	ND	8	0			

			Eutreptiales	6	3	ND	ND	6	0			
	Metamonada			6		ND	ND	6	0	2 (Naoji Yubuki: UBC, Kiyotaka Takishita: JAMSTEC)	0	
			Carpediemonadida	3	3	ND	ND	3	0			
			Diplomonadida	1	3	ND	ND	1	0			
			Retortamonadida	1	3	ND	ND	1	0			
			Trichomonadida	1	3	ND	ND	1	0			
	Percolozoa			ND	1	ND	ND	ND	0	ND	ND	
Eukarya/Protista/Rhizaria				2,869		383	594	3,463	0			
	Cercozoa			143		ND	4	147	0	1 (Kozo Takahashi: Kyushu University)	5	Takahashi K, Anderson OR （2000） Class Phaeodaria. In: Lee JJ, Leedale GF, Bradbury P, editors. An Illustrated Guide to the Protozoa（2nd edition）. Organisms traditionally referred to as Protozoa, or newly discovered groups. Kansas: Society of Protozoologists. pp. 981-994.
			Phaeodaria	143	5	ND	4	147	0			
	Granuloreticulosa			2,321		383	490	2,811	0	5 (Shiro Hasegawa: Kumamoto University, Yasumochi Matoba: SFL, et al.)	6	Nomura R（1987）List of recent benthic foraminifera reported from the seas around the Japanese Islands, 1982-1997. Mem Fac Edu, Shimane Univ, Nat Sci 31: 1-25.
		Foraminifera		2,321		383	490	2,811	0			
			Allogromiina	2	3	0	1	3	0			
			Carterinina	1	5	0	0	1	0			
			Globigerinina	47	5	0	4	51	0			
			Involutinina	4	5	1	0	4	0			
			Lagenina	404	5	72	66	470	0			
			Miliolina	340	5	33	77	417	0			
			Robertinida	19	4	4	6	25	0			
			Rotaliina	989	5	204	189	1,178	0			
			Spirillinina	15	5	0	3	18	0			
			Textulariina	500	5	69	144	644	0			
	Radiozoa			405		ND	100	505	0		35	
		Acantharea		66		ND	ND	66	0	0	10	Bernstein RE, Kling SK, Boltovskoy D （1999）Acantharia. In: Boltovskoy E, editor. South Atlantic zooplankton. Leiden: Backhuys Publishers. pp. 75-147.
			Arthracanthida	51	4	ND	ND	51	0			
			Chaunacanthida	3	3	ND	ND	3	0			
			Holacanthida	4	3	ND	ND	4	0			
			Symphyacanthida	8	3	ND	ND	8	0			
		Polycystinea		338		ND	100	438	0	5 (Noritoshi Suzuki: Tohoku University, Atsushi Matsuoka: Niigata University et al.)	20	Itaki T（2009）Late glacial to Holocene Polycystine radiolarians from the Japan Sea, Special Volume. News Osaka Micropaleontol 14: 43-89.

			Collodaria	31	4	ND	20	51	0			
			Entactinaria	25	4	ND	20	45	0			
			Nassellaria	168	5	ND	20	188	0			
			Spumellaria	114	5	ND	40	154	0			
		incertae sedis		1		ND	0	1	0	0	1	
			Taxopodia	1	5	ND	0	1	0			
Eukarya/Protista/incertae sedis				ND		ND	ND	ND	ND	ND	ND	
	Heliozoa			ND	1	ND	ND	ND	ND			
Eukarya/Fungi/Opisthokonta				367		ND	ND	367	0	2 (Takahiko Nagahama: JAMSTEC, Akira Nakagiri: NITE)	3	Benson, D. A., Karsch-Mizrachi, I., Lipman, D. J., Ostell, J., & Wheeler, D. L. (2008). GenBank. Nucleic Acids Research, 36(Database issue), D25-30. doi: 10.1093/nar/gkm929., http://www.cbs.knaw.nl/databases/, http://www.nbrc.nite.go.jp/NBRC2/NBRCDispSearchServlet?lang=en
	Ascomycota			294		ND	ND	294	0			
		Dothideomycetes		38		ND	ND	38	0			
			Capnodiales	2	3	ND	ND	2	0			
			Dothideales	2	3	ND	ND	2	0			
			Pleosporales	31	4	ND	ND	31	0			
			Tubeufiaceae	3	3	ND	ND	3	0			
		Dothideomycetes et Chaetothyriomycetes		6		ND	ND	6	0			
			Cucurbitariaceae	3	3	ND	ND	3	0			
			Dothioraceae	3	3	ND	ND	3	0			
		Eurotiomycetes		30		ND	ND	30	0			
			Chaetothyriales	4	3	ND	ND	4	0			
			Eurotiales	22	4	ND	ND	22	0			
			Onygenales	4	3	ND	ND	4	0			
		Lecanoromycetes		1		ND	ND	1	0			
			Lecanorales	1	3	ND	ND	1	0			
		Leotiomycetes		24		ND	ND	24	0			
			Helotiales	18	4	ND	ND	18	0			
			Myxotrichaceae	1	3	ND	ND	1	0			
			Pseudeurotiaceae	5	3	ND	ND	5	0			
		Orbiliomycetes		1		ND	ND	1	0			
			Orbiliales	1	3	ND	ND	1	0			
		Saccharomycetes		47		ND	ND	47	0			
			Saccharomycetales	47	4	ND	ND	47	0			
		Sordariomycetes		138		ND	ND	138	0			

			Chaetosphaeriales	1	3	ND	ND	1	0			
			Diaporthales	1	3	ND	ND	1	0			
			Halosphaeriales	43	4	ND	ND	43	0			
			Hypocreales	26	4	ND	ND	26	0			
			Hypocreomycetidae	3	3	ND	ND	3	0			
			Lulworthiales	9	3	ND	ND	9	0			
			Melanosporales	2	3	ND	ND	2	0			
			Microascales	19	4	ND	ND	19	0			
			Phyllachorales	2	3	ND	ND	2	0			
			Sordariales	12	4	ND	ND	12	0			
			Xylariales	16	4	ND	ND	16	0			
			Apiosporaceae	4	3	ND	ND	4	0			
		incertae sedis		9		ND	ND	9	0			
			“Dematiaceae”	3	3	ND	ND	3	0			
			“Myxotrichaceae”	1	3	ND	ND	1	0			
			“Hyphomycetes”	5	3	ND	ND	5	0			
	Basidiomycota			65		ND	ND	65	0			
		Agaricomycetes		6		ND	ND	6	0			
			Agaricales	2	3	ND	ND	2	0			
			Auriculariales	1	3	ND	ND	1	0			
			Polyporales	2	3	ND	ND	2	0			
			Russulales	1	3	ND	ND	1	0			
		Agaricostilbomycetes		3		ND	ND	3	0			
			Agaricostilbomycetes	3	3	ND	ND	3	0			
		Cystobasidiomycetes		9		ND	ND	9	0			
			Erythrobasidiales	9	3	ND	ND	9	0			
		Exobasidiomycetes		3		ND	ND	3	0			
			Exobasidiales	1	3	ND	ND	1	0			
			Georgefischeriales	1	3	ND	ND	1	0			
			Microstromatales	1	3	ND	ND	1	0			
		Microbotryomycetes		16		ND	ND	16	0			
			Leucosporidiales	1	3	ND	ND	1	0			
			Sporidiobolales	14	4	ND	ND	14	0			
			Microbotryomycetes	1	3	ND	ND	1	0			
		Tremellomycetes		27		ND	ND	27	0			
			Cystofilobasidiales	5	3	ND	ND	5	0			
			Filobasidiales	9	3	ND	ND	9	0			

			Tremellales	13	3	ND	ND	13	0			
		Ustilaginomycetes		1		ND	ND	1	0			
			Ustilaginales	1	3	ND	ND	1	0			
	Blastocladiomycota			ND	1	ND	ND	ND	0			
	Chytridiomycota			ND	1	ND	ND	ND	0			
	Glomeromycota			ND	1	ND	ND	ND	0			
	Microsporidia			ND	1	ND	ND	ND	0			
	Zygomycota			8		ND	ND	8	0			
		"Mucoromycotina"		8		ND	ND	8	0			
			Mortierellales	2	3	ND	ND	2	0			
			Mucorales	6	3	ND	ND	6	0			
Eukarya/Animalia/ Opisthokonta				25,767		1,489	121,318	147,085	37			
	Acanthocephala			ND	1	ND	ND	ND	ND	ND	ND	
	Acoelomorpha			10		ND	50	60	0	1 (Ken-ichi Tajika: Nihon University)	1	http://research2.kahaku.go.jp/ujssb/search
		Acoela		10		ND	50	60	0			
			Acoela	10	4	ND	50	60	0			
	Annelida			1,076		6	124	1,200	10			
		Hirudinea		10	4	ND	ND	10	0	1 (Yoshikazu Takashima)	ND	
		Oligochaeta		20	4	ND	ND	20	0	ND	1	高島義和（1999）日本産海産イトミミズ類の分類（1）海産貧毛類の研究の現状.海洋と生物 124: 394-398.
		Polychaeta		1,046		6	124	1,170	10	6 (Eijiro Nishi: YNU, Tetsuya Kato: Kyoto University, et al.)	4	西栄二郎・加藤哲哉・高島義和（2003）環形動物. In: 西田睦・鹿谷法一・諸喜田茂充, 編. 琉球列島の陸水生物.東京, 東海大学出版会. pp.181-189.
			Amphinomida	21	4	ND	ND	21	0			
			Capitellida	48	4	ND	ND	48	0			
			Cossurida	3	4	ND	ND	3	0			
			Ctenodrilidae	ND	1	ND	ND	ND	0			
			Diurodrilida	1	3	ND	ND	1	0			
			Eunicida	129	5	ND	5	134	0			
			Flabelligerida	6	5	ND	1	7	0			
			Myzostomida	30	4	2	ND	30	0			
			Opheliida	12	4	ND	ND	12	0			
			Orbiniida	23	4	ND	ND	23	0			
			Oweniida	6	3	ND	ND	6	0			
			Parergodrilida	ND	1	ND	ND	ND	0			
			Phyllodocida	466	5	1	ND	466	4			

			Poeobiida	1	3	ND	ND	1	0			
			Polygordiida	2	3	1	ND	2	0			
			Protodrilida	2	3	ND	ND	2	0			
			Psammodrilida	ND	1	ND	ND	ND	0			
			Qeustida	ND	1	ND	ND	ND	0			
			Sabellida	130	4	ND	40	170	3			
			Spintherida	3	3	ND	ND	3	0			
			Spionida	89	4	2	42	131	3			
			Sternaspida	1	3	ND	1	2	0			
			Terebellidae	73	4	ND	35	108	0			
			Yndolacida	ND	1	ND	ND	ND	0			
	Arthropoda			6,393		292	1,677	8,070	10			
		Branchiopoda		7		ND	ND	7	0	1 (Takashi Onbé: Hiroshima University)	2	Egloff DA, Fofonoff PW, Onbé T（1997）Reproductive biology of marine Cladocerans. Adv Mar Biol. 31: 79-167.
			Podonidae	6	3	ND	ND	6	0			
			Sididae	1	3	ND	ND	1	0			
		Cephalocarida		1		ND	ND	1	0	ND	ND	http://research2.kahaku.go.jp/ujssb/search
			Brachypoda	1	2	ND	ND	1	0			
		Insecta		7		ND	ND	7	0			
			Hemiptera	4	2	ND	ND	4	0			
			Diptera	3	2	ND	ND	3	0			
		Malacostraca		4,074		195	1,539	5,613	3			
			Amphionidacea	1	2	ND	ND	1	0	ND	ND	http://research2.kahaku.go.jp/ujssb/search
			Amphipoda	544	4	ND	864	1,408	0		>2	
			Bathynellacea	15	4	ND	ND	15	0			
			Cumacea	80	4	ND	ND	80	0			菊地知彦（1997）クーマ類. In: 奥谷喬司・今福道夫・武田正倫　編.日本動物大百科 7 無脊椎動物，東京：平凡社. p. 139.
			Decapoda	2,501	5	28	ND	2,501	3	5 (Komai Tomoyuki: NHMIC, Hironori Komatsu: NMNS, et al.)	8	
			Euphausiacea	86	5	49	5	91	0	3 (Shozo Sawamoto: Tokai University, Nobunari Endo: Tohoku University, et al.)	5	Sawamoto S（1979）Geographical distribution of euphausiids in the western part of the subtropical North Pacific Ocean. The Kuroshio IV, Pro 4th CSK Symp. Tokyo. 651-664.
			Leptostraca	4	3	ND	ND	4	0	ND	>2	菊地知彦（1997）クーマ類. In: 奥谷喬司・今福道夫・武田正倫　編.日本動物大百科 7 無脊椎動物，東京：平凡社. p. 139.
			Lophogastrida	14	4	1	ND	14	0	2 (Kouki Fukuoka: SNF, Masaaki Murano)	1	千原光雄・村野正昭（1997）日本産海洋プランクトン検索図説. 東京：東海大学出版会. 1574 p.

			Mysida	226	5	117	ND	226	0		3	
			Stomatopoda	56	4	ND	ND	56	0	ND	>2	http://research2.kahaku.go.jp/ujssb/search
			Tanaidacea	92	4	ND	ND	92	0	3 (Nobuhiro Saito: Suido-sha, Michitaka Shimomura: KMNH, et al.)	4	Larsen K, Shimomura M（2006）Tanaidacea （Crustacea: Peracarida）from Japan. I. Apseudomorpha from the East China Sea, Seto Inland Sea, and Nansei Islands. Zootaxa 1341: 29-48.
			Thermosbaenacea	1	3	ND	ND	1	0	1 (Michitaka Shimomura: KMNH)	ND	Shimomura M, Fujita Y（2009）First record of the thermosbaenacean genus *Halosbaena* from Asia: *H. daitoensis* sp. nov. （Peracarida: Thermosbaenacea: Halosbaenidae） from an anchihaline cave of Minamidaito-jima Is., in Okinawa, southern Japan. Zootaxa. 1990: 55-64.
			Isopoda	454	5	ND	670	1,124	0	7 (Michitaka Shimomura: KMNH, Noboru Nunomura: TSM, et al.)	3	
		Maxillopoda		2,150		37	118	2,268	7			
			Arguloida	5	3	ND	ND	5	0	1 (Kazuya Nagasawa: Hiroshima University)	2	http://www.jodc.go.jp/data/biology.html
			Calanoida	743	5	22	ND	743	0	5 (Susumu Ohtsuka: Hiroshima University, Shuhei Nishida: University of Tokyo, et al.)		
			Cyclopoida	78	4	ND	ND	78	0	4 (Shuhei Nishida: University of Tokyo, Hiroshi Ueda: Kochi University, et al.)	3	
			Dendrogastrida	3	3	1	8	11	0	2 (Mark J. Grygier: LBM, Miéko Komatsu: University of Toyama)	2	Haramoto S, Komatsu M, Yamazaki Y（2007）Patterns of asexual reproduction in the fissiparous seastar *Coscinasterias acutispina*（Asteroidea: Echinodermata）in Japan. Zool Sci 24: 1075-1081.
			Facetotecta	10	4	ND	40	50	0	2 (Mark J. Grygier: LBM, Tomohiko Kikuchi: YNU)	1	Glenner H, Høeg JT, Grygier MJ, Fujita Y（2008）Induced metamorphosis in crustacean y-larvae: Towards a solution to a 100-year-old riddle. BMC Biology 6: 21.
			Halocyprida	11	4	ND	ND	11	0	1 (Akira Tsukagoshi: Shizuoka University)	>2	http://research2.kahaku.go.jp/ujssb/search
			Harpacticoida	140	5	ND	ND	140	0	3 (Nozomu Iwasaki: Kochi University, Shinji Shimode: University of Tokyo, et al.)	3	http://www.jodc.go.jp/data/biology.html
			Laurida	10	4	3	13	23	0	1 (Mark J. Grygier: LBM)	2	Grygier MJ（1996）A world list of coral reef-associated Ascothoracida (Crustacea), with remarks on Lauridae, Petrarcidae, and Dendrogastridae from Okinawa, Japan. Galaxea 13: 15-33.
			Lernaeodiscidae	2	3	ND	ND	2	0	ND	>1	http://research2.kahaku.go.jp/ujssb/search
			Misophrioida	3	3	1	ND	3	0	1 (Susumu Ohtsuka: Hiroshima University)	3	http://www.jodc.go.jp/data/biology.html
			Monstrilloida	12	4	5	ND	12	0	2 (Susumu Ohtsuka: Hiroshima University, Mark J. Grygier: LBM)		
			Mormonilloida	2	3	0	ND	2	0	1 (Susumu Ohtsuka: Hiroshima University)		
			Myodocopida	25	4	ND	ND	25	0	1 (Akira Tsukagoshi: Shizuoka University)	2	http://research2.kahaku.go.jp/ujssb/search
			Pedunculata	66	4	ND	28	94	0	1 (Toshiyuki Yamaguchi: Chiba University)	>2	

			Peltogastriidae	4	3	ND	ND	4	0	ND	>1	
			Platycopida	2	3	ND	ND	2	0	1 (Akira Tsukagoshi: Shizuoka University)		
			Platycopioida	2	3	2	ND	2	0	1 (Susumu Ohtsuka: Hiroshima University)	3	http://www.jodc.go.jp/data/biology.html
			Podocopida	284	5	ND	ND	284	0	1 (Akira Tsukagoshi: Shizuoka University)	>1	http://research2.kahaku.go.jp/ujssb/search
			Poecilostomatoida	321	5	ND	ND	321	0	5 (Kazuya Nagasawa: Hiroshima University, Shuhei Nishida: University of Tokyo et al.)	3	http://www.jodc.go.jp/data/biology.html
			Sessilia	154	5	ND	29	183	6	1 (Toshiyuki Yamaguchi: Chiba University)	>2	http://research2.kahaku.go.jp/ujssb/search
			Siphonostomatoida	253	5	ND	ND	253	1	2 (Kazuya Nagasawa: Hiroshima University, Susumu Ohtsuka: Hiroshima University)	1	http://www.jodc.go.jp/data/biology.html
			Succulinidae	13	4	ND	ND	13	0	ND	>1	http://research2.kahaku.go.jp/ujssb/search
			Sylonidae	1	3	ND	ND	1	0			
			Tantulocarida	4	3	3	ND	4	0	1 (Susumu Ohtsuka: Hiroshima University)	3	http://www.jodc.go.jp/data/biology.html
			Thompsoniidae	2	3	ND	ND	2	0	ND	>1	http://research2.kahaku.go.jp/ujssb/search
		Merostoma		1		ND	0	1	0			
			Xiphosurida	1	5	ND	0	1	0			
		Pycnogonida		153		60	20	173	0	1 (Ko-ichiro Nakamura)	3	Nakamura K, Child CA（1991）Pycnogonida from waters adjacent to Japan. Smithsonian Cont Zool. 512.
			Pantopoda	153	5	60	20	173	0			
	Brachiopoda			73		ND	27	100	0	2 (Hiroshi Kajihara: Hokkaido University, Shunsuke Mawatari: Hokkaido University)	>1	http://research2.kahaku.go.jp/ujssb/search
		Craniata		1		ND	2	3	0			
			Craniida	1	3	ND	2	3	0			
		Lingulata		5		ND	4	9	0			
			Lingulida	5	3	ND	4	9	0			
		Rhynchonellata		67		ND	21	88	0			
			Rhynchonellida	13	5	ND	2	15	0			
			Terebratulida	53	4	ND	16	69	0			
			Thecideida	1	3	ND	3	4	0			
	Chaetognatha			36		5	6	42	0	4 (Taichiro Goto: Mie University, Kazuki Kuroda: JSNFRI, et al.)	1	
		Sagittoidea		36		5	6	42	0			
			Aphragmophora	25	5	0	0	25	0			
			Biphragmophora	1	3	1	1	2	0			
			Monophragmophora	10	4	4	5	15	0			
	Choanozoa			54	4	ND	ND	54	0	ND	>1	http://research2.kahaku.go.jp/ujssb/search
	Chordata			4,330		364	326	4,656	3			

		Ascidiacea		313		ND	0	313	2	1 (Teruaki Nishikawa: Toho University)	>2	http://research2.kahaku.go.jp/ujssb/search
			Enterogona	173	5	ND	0	173	0			
			Pleurogona	140	5	ND	0	140	2			
		Appendicularia		31		0	6	37	0	2 (Naonobu Shiga: HJC, Riki Sato: AMSL)	3	千原光雄・村野正昭（1997）日本産海洋プランクトン検索図説. 東京：東海大学出版会. 1574 p.
			Fritillariidae	15	5	0	2	17	0			
			Kowalevskiidae	1	5	0	0	1	0			
			Oikopleuridae	15	4	0	4	19	0			
		Aves		84		ND	0	84	0	>1	>50	http://www3.town.haboro.hokkaido.jp/seabird/
			Charadriiformes	49	5	ND	0	49	0			
			Pelecaniformes	12	5	ND	0	12	0			
			Procellariiformes	23	5	ND	0	23	0			
		Leptocerdia		4		ND	ND	4	0	1 (Teruaki Nishikawa: Toho University)	>1	http://research2.kahaku.go.jp/ujssb/search
			Branchiostomatidae	4	3	ND	ND	4	0			
		Mammalia		46		0	1	47	0			
			Cetartiodactyla	39	5	0	1	40	0	3 (Tadasu Yamada: NMNS, Masao Amano: Nagoya University, et al.)	>5	http://www.iwcoffice.org/conservation/cetacea.htm, http://www.iucnredlist.org/
			Carnivora	7	5	0	0	7	0	3 (Naoki Kohno: NMNS, Kazuo Wada: TAT, et al.)		
		Pisces		3,790		364	317	4,107	1	15 (Keiichi Matsuura: NMNS, Tetsuji Nakabo: Kyoto University, et al.)	>50	http://research2.kahaku.go.jp/ujssb/search
			Acanthuridae	39	5	0	0	39	0			
			Acipenseridae	3	5	0	0	3	0			
			Acropomatidae	12	5	2	0	12	0			
			Agonidae	23	5	4	0	23	0			
			Albulidae	1	5	0	0	1	0			
			Alepisauridae	2	5	0	0	2	0			
			Alepocephalidae	20	5	5	0	20	0			
			Alopiidae	3	5	0	0	3	0			
			Ambassidae	5	5	0	0	5	0			
			Ammodytidae	4	5	1	0	4	0			
			Anarhichadidae	1	5	1	0	1	0			
			Anguillidae	2	5	0	0	2	0			
			Anomalopidae	1	5	0	0	1	0			
			Anoplogastridae	1	5	0	0	1	0			
			Anoplopomatidae	2	5	0	0	2	0			
			Antennariidae	14	5	1	0	14	0			

			Aphyonidae	1	5	0	0	1	0			
			Aploactinidae	6	4	4	3	9	0			
			Apogonidae	87	5	1	0	87	0			
			Aracanidae	2	4	0	1	3	0			
			Argentinidae	3	5	0	0	3	0			
			Ariidae	3	5	0	0	3	0			
			Ariommatidae	3	5	0	0	3	0			
			Astronesthidae	16	5	0	0	16	0			
			Ateleopodidae	5	5	1	0	5	0			
			Atherinidae	6	4	0	3	9	0			
			Aulopidae	4	5	0	0	4	0			
			Aulorhynchidae	1	5	0	0	1	0			
			Aulostomidae	1	5	0	0	1	0			
			Balistidae	21	5	1	1	22	0			
			Banjosidae	1	5	0	0	1	0			
			Barbourisiidae	1	5	0	0	1	0			
			Bathyclupeidae	1	5	0	0	1	0			
			Bathymasteridae	3	5	0	0	3	0			
			Bathysauridae	1	5	0	0	1	0			
			Bathysauroides	1	5	0	0	1	0			
			Belonidae	6	5	0	0	6	0			
			Bembridae	2	5	0	0	2	0			
			Berycidae	4	5	0	0	4	0			
			Blenniidae	73	5	3	0	73	0			
			Bothidae	36	5	4	0	36	0			
			Bramidae	10	5	0	0	10	0			
			Branchiostegidae	5	5	0	1	6	0			
			Bregmacerotidae	6	5	1	0	6	0			
			Bythitidae	9	5	2	0	9	0			
			Caesionidae	10	5	0	0	10	0			
			Callanthiidae	3	5	0	0	3	0			
			Callionymidae	37	5	7	0	37	0			
			Caproidae	3	5	0	0	3	0			
			Caracanthidae	2	5	0	0	2	0			
			Carangidae	56	5	0	1	57	0			
			Carapidae	12	5	3	0	12	0			
			Carcharhinidae	22	5	0	2	24	0			

			Caristiidae	1	5	0	0	1	0			
			Caulophrynidae	2	5	0	0	2	0			
			Centriscidae	2	5	0	0	2	0			
			Centrogenysidae	1	5	0	0	1	0			
			Centrolophidae	4	5	1	0	4	0			
			Centrophoridae	8	5	3	0	8	0			
			Cepolidae	8	5	2	0	8	0			
			Ceratiidae	3	5	0	0	3	0			
			Cetomimidae	7	5	1	0	7	0			
			Cetorhinidae	1	5	0	0	1	0			
			Chaenopsidae	6	5	3	0	6	0			
			Chaetodontidae	51	5	0	0	51	0			
			Champsodontidae	4	5	0	0	4	0			
			Chanidae	1	5	0	0	1	0			
			Chauliodontidae	2	5	0	0	2	0			
			Chaunacidae	3	5	0	0	3	0			
			Cheilodactylidae	3	5	2	0	3	0			
			Chiasmodontidae	6	5	0	0	6	0			
			Chimaeridae	8	5	5	0	8	0			
			Chirocentridae	1	5	0	0	1	0			
			Chlamydoselachidae	1	5	0	0	1	0			
			Chlopsidae	4	5	0	0	4	0			
			Chlorophthalmidae	7	5	1	0	7	0			
			Cirrhitidae	14	5	0	0	14	0			
			Citharidae	3	5	0	0	3	0			
			Clinidae	2	5	1	0	2	0			
			Clupeidae	17	5	0	0	17	0			
			Colocongridae	2	5	0	0	2	0			
			Congridae	25	5	5	0	25	0			
			Congrogadidae	1	5	0	0	1	0			
			Coryphaenidae	2	5	0	0	2	0			
			Cottidae	80	5	37	6	86	0			
			Creediidae	4	5	1	0	4	0			
			Cryptacanthodidae	1	5	1	0	1	0			
			Cyclopteridae	10	5	0	0	10	0			
			Cyematidae	1	5	0	0	1	0			
			Cynoglossidae	18	5	1	0	18	0			

			Dactylopteridae	3	5	0	0	3	0			
			Dalatiidae	5	5	0	0	5	0			
			Dasyatidae	15	5	2	0	15	0			
			Derichthyidae	2	5	0	0	2	0			
			Diceratiidae	1	5	0	0	1	0			
			Diodontidae	7	5	0	0	7	0			
			Diretmidae	2	5	0	0	2	0			
			Draconettidae	4	5	0	0	4	0			
			Drepanidae	2	5	0	0	2	0			
			Echeneidae	8	5	0	0	8	0			
			Echinorhinidae	2	5	0	0	2	0			
			Eleotridae	12	5	0	3	15	0			
			Elopidae	1	5	0	0	1	0			
			Embiotocidae	3	5	1	0	3	0			
			Emmelichthyidae	4	5	1	0	4	0			
			Engraulidae	9	5	1	0	9	0			
			Ephippididae	5	5	0	0	5	0			
			Epigonidae	5	5	1	0	5	0			
			Ereuniidae	2	5	2	0	2	0			
			Etmopteridae	12	5	1	0	12	0			
			Eurypharyngidae	1	5	0	0	1	0			
			Evermannellidae	4	5	0	0	4	0			
			Exocoetidae	30	5	1	0	30	0			
			Fistulariidae	2	5	0	0	2	0			
			Gadidae	4	5	0	0	4	0			
			Gasterosteidae	8	5	2	0	8	0			
			Gempylidae	12	5	0	0	12	0			
			Gerreidae	10	4	0	3	13	0			
			Gigantactinidae	5	5	0	0	5	0			
			Giganthiidae	1	5	0	0	1	0			
			Girellidae	3	5	0	0	3	0			
			Glaucosomatidae	1	5	0	0	1	0			
			Gobiesocidae	11	5	2	0	11	0			
			Gobiidae	367	5	28	216	583	0			
			Gonorynchidae	1	5	0	0	1	0			
			Gonostomatidae	12	5	0	0	12	0			
			Grammicolepididae	2	5	0	0	2	0			

			Gymnuridae	2	5	0	0	2	0			
			Haemulidae	20	5	0	0	20	0			
			Halosauridae	2	5	0	0	2	0			
			Hemiramphidae	9	5	0	0	9	0			
			Hemiscyllidae	4	5	0	0	4	0			
			Hemitripteridae	4	5	0	0	4	0			
			Heterodontidae	2	5	1	0	2	0			
			Hexagrammidae	7	5	0	0	7	0			
			Hexanchidae	3	5	0	0	3	0			
			Hexatrygonidae	1	5	0	0	1	0			
			Himantolophidae	2	5	1	0	2	0			
			Holocentridae	38	5	2	1	39	0			
			Hoplichthyidae	5	5	1	0	5	0			
			Howellidae	4	5	1	1	5	0			
			Hypoptychidae	1	5	0	0	1	0			
			Icosteidae	1	5	0	0	1	0			
			Idiacanthidae	2	5	0	0	2	0			
			Ipnopidae	2	5	0	0	2	0			
			Istiophoridae	5	5	0	0	5	0			
			Kraemeriidae	3	5	0	0	3	0			
			Kuhliidae	4	5	0	0	4	0			
			Kyphosidae	5	5	0	0	5	0			
			Labridae	144	5	0	0	144	0			
			Lamnidae	4	5	0	0	4	0			
			Lampridae	1	5	0	0	1	0			
			Latidae	2	5	1	0	2	0			
			Leiognathidae	11	5	0	1	12	0			
			Lethrinidae	27	5	0	1	28	0			
			Linophrynidae	2	5	0	0	2	0			
			Liparidae	46	5	13	4	50	0			
			Lipogenyidae	1	5	0	0	1	0			
			Lobotidae	1	5	0	0	1	0			
			Lophiidae	7	5	0	0	7	0			
			Lophotidae	2	5	0	0	2	0			
			Loricariidae	1	5	0	0	1	0			
			Lutjanidae	49	5	0	2	51	0			
			Luvaridae	1	5	0	0	1	0			

			Macroramphosidae	1	5	0	0	1	0			
			Macrouridae	68	5	25	0	68	0			
			Macrouroididae	1	5	0	0	1	0			
			Macrurocyttidae	1	5	0	0	1	0			
			Malacanthidae	5	5	0	0	5	0			
			Malacosteidae	3	5	0	0	3	0			
			Megachasmidae	1	5	0	0	1	0			
			Megalomycteridae	1	5	0	0	1	0			
			Megalopidae	1	5	0	0	1	0			
			Melamphaidae	10	5	0	0	10	0			
			Melanocetidae	2	5	0	0	2	0			
			Melanonidae	1	5	0	0	1	0			
			Melanostomiidae	35	5	2	0	35	0			
			Menidae	1	5	0	0	1	0			
			Merlucciidae	1	5	0	0	1	0			
			Microcanthidae	1	5	0	0	1	0			
			Microdesmidae	24	5	0	2	26	0			
			Microstomatidae	8	5	1	0	8	0			
			Mirapinnidae	1	5	0	0	1	0			
			Mitsukurinidae	1	5	0	0	1	0			
			Molidae	3	5	0	0	3	0			
			Monacanthidae	31	5	1	1	32	0			
			Monocentridae	1	5	0	0	1	0			
			Monodactylidae	1	5	0	0	1	0			
			Monognathidae	1	5	1	0	1	0			
			Moridae	18	5	9	0	18	0			
			Moringuidae	4	5	0	0	4	0			
			Moronidae	3	5	1	0	3	1			
			Mugilidae	15	5	0	0	15	0			
			Mullidae	22	5	0	2	24	0			
			Muraenesocidae	4	5	0	0	4	0			
			Muraenidae	54	5	0	0	54	0			
			Myctophidae	88	5	1	0	88	0			
			Myliobatidae	9	5	0	0	9	0			
			Myrocongridae	1	5	0	0	1	0			
			Myxinidae	5	5	1	0	5	0			
			Nemichthyidae	3	5	0	0	3	0			

			Nemipteridae	21	5	0	0	21	0			
			Neoscopelidae	4	5	0	0	4	0			
			Nettastomatidae	5	5	0	0	5	0			
			Nomeidae	8	5	0	0	8	0			
			Notacanthidae	3	5	0	0	3	0			
			Notocheiridae	1	5	1	0	1	0			
			Notorynchidae	1	5	0	0	1	0			
			Notosudidae	5	5	0	0	5	0			
			Odontaspididae	2	5	0	0	2	0			
			Odontobutidae	1	3	2	1	2	0			
			Ogcocephalidae	19	5	1	1	20	0			
			Oneirodidae	6	5	0	0	6	0			
			Ophichthidae	40	5	13	0	40	0			
			Ophidiidae	30	5	10	0	30	0			
			Opisthoproctidae	5	5	0	0	5	0			
			Opistognathiae	9	5	5	0	9	0			
			Oplegnathidae	2	5	0	0	2	0			
			Orectolobidae	3	5	0	0	3	0			
			Oreosomatidae	1	5	0	0	1	0			
			Osmeridae	7	5	0	0	7	0			
			Ostraciidae	9	5	0	0	9	0			
			Ostracoberycidae	2	5	1	0	2	0			
			Oxynotidae	1	5	1	0	1	0			
			Parabembridae	1	5	0	0	1	0			
			Parabrotulidae	2	5	1	0	2	0			
			Paralepididae	21	5	1	0	21	0			
			Paralichthyidae	10	5	1	0	10	0			
			Parascylliidae	2	5	1	0	2	0			
			Paraulopidae	4	5	2	0	4	0			
			Parazenidae	1	5	0	0	1	0			
			Pegasidae	3	5	0	0	3	0			
			Pempheridae	5	5	0	0	5	0			
			Pentacerotidae	4	5	0	0	4	0			
			Percophidae	13	5	0	0	13	0			
			Peristediidae	15	5	4	0	15	0			
			Petromyzontidae	4	5	0	0	4	0			
			Pholidae	6	5	1	0	6	0			

			Phosichthyidae	9	5	0	0	9	0			
			Pinguipedidae	24	5	0	0	24	0			
			Platycephalidae	17	5	0	0	17	0			
			Platyrhinidae	1	5	0	0	1	0			
			Platytroctidae	3	5	0	0	3	0			
			Plecoglossidae	2	5	1	0	2	0			
			Plesiobatididae	1	5	0	0	1	0			
			Plesiopidae	9	5	0	0	9	0			
			Pleuronectidae	33	5	0	0	33	0			
			Plotosidae	1	5	0	0	1	0			
			Poeciliidae	2	5	0	0	2	0			
			Poecilopsettidae	1	5	0	0	1	0			
			Polymixiidae	4	5	1	0	4	0			
			Polynemidae	4	5	0	0	4	0			
			Polyprionidae	2	5	1	0	2	0			
			Pomacanthidae	30	5	1	0	30	0			
			Pomacentridae	99	5	2	0	99	0			
			Priacanthidae	10	5	0	1	11	0			
			Pristidae	1	5	0	0	1	0			
			Pristiophoridae	1	5	0	0	1	0			
			Proscylliidae	2	5	1	0	2	0			
			Pseudocarcharinidae	1	5	0	0	1	0			
			Pseudochromidae	7	5	0	0	7	0			
			Pseudoplesiopidae	1	5	0	0	1	0			
			Pseudotriakidae	1	5	0	0	1	0			
			Pseudotrichonotidae	1	5	1	0	1	0			
			Psychrolutidae	11	5	3	1	12	0			
			Pterothrissidae	1	5	1	0	1	0			
			Ptilichthyidae	1	5	0	0	1	0			
			Rachycentridae	1	5	0	0	1	0			
			Rajidae	33	5	9	0	33	0			
			Regalecidae	1	5	0	0	1	0			
			Rhamphocottidae	1	5	0	0	1	0			
			Rhincodontidae	3	5	0	0	3	0			
			Rhinobatidae	3	5	0	0	3	0			
			Rhinochimaeridae	3	5	0	0	3	0			
			Rhyacichthyidae	1	5	0	0	1	0			

			Rhynchobatidae	2	5	0	0	2	0			
			Rondeletiidae	1	5	0	0	1	0			
			Salangidae	4	5	2	0	4	0			
			Salmonidae	20	5	6	0	20	0			
			Samaridae	5	5	1	0	5	0			
			Scaridae	34	5	0	0	34	0			
			Scatophagidae	1	5	0	0	1	0			
			Schindleriidae	1	3	0	8	9	0			
			Sciaenidae	17	5	0	0	17	0			
			Scomberesocidae	1	5	0	0	1	0			
			Scombridae	21	5	0	0	21	0			
			Scombrolabracidae	1	5	0	0	1	0			
			Scombropidae	2	5	1	0	2	0			
			Scopelarchidae	11	5	0	0	11	0			
			Scorpaenidae	99	5	15	12	111	0			
			Scorpididae	1	5	0	0	1	0			
			Scyliorhinidae	16	5	5	0	16	0			
			Serranidae	129	5	8	0	129	0			
			Serrivomeridae	2	5	0	0	2	0			
			Siganidae	12	5	0	0	12	0			
			Sillaginidae	4	5	0	0	4	0			
			Sinipercidae	1	5	0	0	1	0			
			Soleidae	17	5	2	0	17	0			
			Solenostomidae	3	5	0	0	3	0			
			Somniosidae	6	5	1	0	6	0			
			Sparidae	13	5	0	1	14	0			
			Sphyraenidae	9	5	0	0	9	0			
			Sphyrnidae	3	5	0	0	3	0			
			Squalidae	8	5	2	0	8	0			
			Squatinidae	2	5	0	0	2	0			
			Sternoptychidae	17	5	2	0	17	0			
			Stichaeidae	34	5	6	1	35	0			
			Stomiidae	3	5	0	0	3	0			
			Stromateidae	4	5	0	0	4	0			
			Symphysanodontidae	2	5	0	0	2	0			
			Synanceiidae	9	5	0	0	9	0			
			Synaphobranchidae	10	5	0	0	10	0			

			Synbranchidae	1	5	0	0	1	0			
			Syngnathidae	51	5	2	0	51	0			
			Synodontidae	23	5	0	0	23	0			
			Teraponidae	7	5	1	0	7	0			
			Tetragonuridae	2	5	0	0	2	0			
			Tetraodontidae	51	5	2	4	55	0			
			Tetrarogidae	8	5	3	0	8	0			
			Torpedinidae	6	5	1	0	6	0			
			Toxotidae	1	5	0	0	1	0			
			Trachichthyidae	6	5	3	0	6	0			
			Trachipteridae	5	5	0	0	5	0			
			Triacanthidae	1	5	0	0	1	0			
			Triacanthodidae	7	5	0	0	7	0			
			Triakidae	5	5	0	0	5	0			
			Trichiuridae	12	5	1	0	12	0			
			Trichodontidae	1	5	0	0	1	0			
			Trichonotidae	3	5	0	0	3	0			
			Triglidae	19	5	2	0	19	0			
			Triodontidae	1	5	0	0	1	0			
			Tripterygiidae	22	5	2	2	24	0			
			Uranoscopidae	7	5	0	0	7	0			
			Urolophidae	1	5	0	0	1	0			
			Veliferidae	2	5	0	0	2	0			
			Xenisthmidae	4	3	0	6	10	0			
			Xiphiidae	1	5	0	0	1	0			
			Zanclidae	1	5	0	0	1	0			
			Zaproridae	1	5	0	0	1	0			
			Zeidae	4	3	0	5	9	0			
			Zoarcidae	67	4	21	19	86	0			
		Reptilia		22		ND	0	22	0			
			Cheloniidae	5	5	ND	0	5	0	>1	>3	http://research2.kahaku.go.jp/ujssb/search
			Dermochelyidae	1	5	ND	0	1	0			
			Squamata	16	5	ND	0	16	0			http://mail2.nara-edu.ac.jp/ ~inoue/NNM/ hatyuurui/ wamei-h.html
		Thaliacea		40		0	2	42	0	1 (Jun Nishikawa: University of Tokyo)	1	西川淳（1997）脊索動物門，尾索動物亜門，被嚢動物亜門，タリア綱. In: 千原光雄・村野正昭，編. 日本産海洋プランクトン検索図説.東京，東海大学出版会. pp. 1351-1392, 1525-1528.

			Doliolida	4	5	0	1	5	0			
			Pyrosomatida	8	5	0	0	8	0			
			Salpida	28	5	0	1	29	0			
	Cnidaria			1,876		350	364	2,240	1			
		Anthozoa		1,296		238	355	1,651	0			
			Actiniaria	174	5	26	30	204	0	2 (Kensuke Yanagi: NHMIC, Hiroomi Uchida: KMPC)	4	Yanagi K (2006) Sea anemones around the Sagami Sea with the list of Japanese species. Mem Nat Sci Mus Tokyo 40: 113-173.
			Alcyonacea	223	5	50	120	343	0	4 (Yukimitsu Imahara: BIK, Fumihito Iwase BIK, et al.)	7	Imahara Y (2006) Preliminary report on the Alcyonacean and Pennatulacean octocorals collected by the Natural History Research of the Sagami Sea. Mem Nat Sci Mus Tokyo 40: 91-101.
			Antipatharia	ND	1	ND	ND	ND	0			
			Ceriantharia	4	3	ND	ND	4	0	1 (Hiroomi Uchida: KMPC)	>5	http://research2.kahaku.go.jp/ujssb/search
			Corallimorpharia	11	4	ND	ND	11	0			
			Gorgonacea	241	5	115	70	311	0	4 (Yukimitsu Imahara: BIK, Fumihito Iwase BIK, et al.)	8	Imahara Y（1996）Previously recorded octocorals from Japan and adjacent seas. Precious Corals Octocoral Res 17-44.
			Helioporacea	1	3	0	ND	1	0		3	
			Pennatulacea	71	4	24	20	91	0		5	Imahara Y (2006) Preliminary report on the Alcyonacean and Pennatulacean octocorals collected by the Natural History Research of the Sagami Sea. Mem Nat Sci Mus Tokyo 40: 91-101.
			Scleractinia	530	5	10	100	630	0	10 (Hironobu Fukami: Kyoto University, Keiichi Nomura: KMPC, et al.)	>10	西平守孝・Veron JEN（1995）日本の造礁サンゴ類. 東京：海游舎. 440 p.
			Stolonifera	18	4	8	10	28	0	1 (Yukimitsu Imahara: BIK)	3	Imahara Y (2006) Preliminary report on the Alcyonacean and Pennatulacean octocorals collected by the Natural History Research of the Sagami Sea. Mem Nat Sci Mus Tokyo 40: 91-101.
			Telestacea	8	3	5	5	13	0		4	Imahara Y（1996）Previously recorded octocorals from Japan and adjacent seas. Precious Corals Octocoral Res 17-44.
			Zoantharia	15	4	ND	ND	15	0	1 (James Davis Reimer: University of the Ryukyus)	>5	http://research2.kahaku.go.jp/ujssb/search
		Cubozoa		4		3	ND	4	0	2 (Shin Kubota: Kyoto University, Hiroshi Namikawa: NMNS)	>5	
			Cubomedusae	4	4	3	ND	4	0			
		Hydrozoa		539		105	9	539	0			
			Actinulida	1	3	ND	ND	1	0			
			Athecata	130	5	53	ND	130	0			
			Laingiomedusa	1	3	0	ND	1	0			
			Limnomedusae	8	3	2	ND	8	0			
			Milleporina	10	4	ND	ND	10	0			
			Narcomedusae	9	3	0	ND	9	0			

			Siphonophora	54	4	4	9	54	0			
			Stylasterina	24	4	ND	ND	24	0			
			Thecata	286	5	46	ND	286	0			
			Trachymedusae	16	4	0	ND	16	0			
		Scyphozoa		37		4	ND	37	1			
			Coronatae	7	3	2	ND	7	0			
			Rhizostomeae	10	4	2	ND	10	1			
			Semaeostomeae	12	4	0	ND	12	0			
			Stauromedusae	8	3	0	ND	8	0			
	Ctenophora			41		15	13	54	0	4 (Takushi Horita, Dhugal J. Lindsay: JAMSTEC, et al.)	3	http://www.catalogueoflife.org/annual-checklist/search.php
		Nuda		6		2	0	6	0			
			Beroida	6	5	2	0	6	0			
		Tentaculata		35		13	13	48	0			
			Cestida	1	5	0	0	1	0			
			Cydippida	12	4	0	6	18	0			
			Ganeshida	0	1	0	1	1	0			
			Lobata	13	5	6	3	16	0			
			Platyctenida	8	5	7	1	9	0			
			Thalassocalycida	1	5	0	0	1	0			
			incertae sedis	ND	1	ND	2	ND	0			
	Cycliophora			ND	1	ND	ND	ND	ND	-		
	Dicyemida			120	5	40	30	150	0	1 (Hidetaka Furuya: Osaka University)	>1	
	Echinodermata			1,052		ND	ND	1,052	0			
		Asteroidea		280		ND	ND	280	0	2 (Toshihiko Fujita: NMNS, Masaki Saba)	1	林良二（1973）相模湾産海星類. 東京：保育社. 239 p.
			Brisingida	15	4	ND	ND	15	0			http://research2.kahaku.go.jp/ujssb/search
			Forcipulitida	47	4	ND	ND	47	0			
			Notomyotida	10	4	ND	ND	10	0			
			Spinulosida	30	4	ND	ND	30	0			
			Paxillosida	51	4	ND	ND	51	0			Saba M, Fujita T（2006）Asteroidea（Echinodermata）from the Sagami Sea, central Japan. 1. Paxillosida and Valvatida. Mem Nat Sci Mus Tokyo 41: 251-287.
			Valvatida	105	5	ND	ND	105	0			
			Velatida	22	4	ND	ND	22	0			http://research2.kahaku.go.jp/ujssb/search
		Crinoidea		111		ND	ND	111	0	2 (Toshihiko Fujita: NMNS, Ichizo Kogo: OMNH)	2	
			Bourgueticrinida	2	3	ND	ND	2	0			http://research2.kahaku.go.jp/ujssb/search
			Comatulida	96	4	ND	ND	96	0			Kogo I（2006）Comatulid Fauna

												（Echinodermata: Crinoidea: Comatulida）of the Sagami Sea and a Part of Izu Islands, central Japan. Mem Nat Sci Mus Tokyo 41: 223-287.
			Hyocrinida	3	3	ND	ND	3	0			Kogo I（1998）Crinoids from Japan and its adjacent waters. Special publication. Osaka Mus Nat Hist 30: 1-148.
			Isocrinida	10	4	ND	ND	10	0			http://research2.kahaku.go.jp/ujssb/search
		Echinoidea		161		ND	ND	161	0	1 (Michio Shigei)	2	Shigei M（2006）A systematical study on the echinoids （Echinoermata, Echinoidea） from the Sagami Sea. Mem Nat Sci Mus, Tokyo 41: 305-327.
			Cassiduloida	4	3	ND	ND	4	0			http://research2.kahaku.go.jp/ujssb/search
			Cidaroida	25	4	ND	ND	25	0			
			Clypeasteroida	25	4	ND	ND	25	0			
			Diadematoida	18	4	ND	ND	18	0			
			Echinoida	35	4	ND	ND	35	0			
			Echinothurioida	11	4	ND	ND	11	0			
			Holectypoida	2	3	ND	ND	2	0			
			Spatangoida	41	4	ND	ND	41	0			
		Holothuroidea		192		ND	ND	192	0	1 (Tohru Imaoka)	2	
			Apodida	31	4	ND	ND	31	0			
			Aspidochirotida	65	4	ND	ND	65	0			
			Dactylochirotida	5	3	ND	ND	5	0			
			Dendrochirotida	50	4	ND	ND	50	0			
			Elapsipodida	26	4	ND	ND	26	0			
			Molpadiida	15	4	ND	ND	15	0			
		Ophiuroidea		308		ND	ND	308	0	2 (Toshihiko Fujita: NMNS, Seiichi Irimura: NMNS)	1	入村精一（1982）相模湾産蛇尾類. 東京：丸善. 95 p.
			Ophiurida	256	5	ND	ND	256	0			
			Euryalida	52	4	ND	ND	52	0			
	Echiura			21		ND	ND	21	0	1 (Teruaki Nishikawa: Toho University)	>2	http://research2.kahaku.go.jp/ujssb/search
		Echiura		21		ND	ND	21	0			
			Echiuroinea	20	4	ND	ND	20	0			
			Xenopneusta	1	3	ND	ND	1	0			
	Ectoprocta/Bryozoa			300	5	ND	900	1,200	0	2 (Hiroshi Kajihara: Hokkaido University, Shunsuke Mawatari: Hokkaido University)	ND	
	Entoprocta			41		33	210	251	0	1 (Tohru Iseto: Kyoto University)	1	
			Barentsiidae	8	3	2	5	13	0			
			Loxokalypodidae	0	1	0	1	1	0			
			Loxosomatidae	30	4	30	200	230	0			

			Pedicellinidae	3	3	1	4	7	0			
	Gastrotricha			12		3	100	112	0	1 (Yoshihisa Shirayama: Kyoto University)	0	
		Gastrotricha		12		3	100	112	0			
			Chaetonotida	6	3	2	50	56	0			
			Macrodasyida	6	3	1	50	56	0			
	Gnathostomulida			5		0	100	105	0	1 (Yoshihisa Shirayama: Kyoto University)		Wolfgang Sterr, Personal Communication
			Bursovaginoidea	5	3	0	95	100	0			
			Filospermoidea	0	1	0	5	5	0			
	Hemichordata			11		ND	10	21	0	1 (Teruaki Nishikawa: Toho University)	>2	http://research2.kahaku.go.jp/ujssb/search
		Enteropneusta		8		ND	ND	8	0			
			Harrimaniidae	2	3	ND	ND	2	0			
			Ptychoderidae	3	3	ND	ND	3	0			
			Saxipendiidae	1	3	ND	ND	1	0			
			Spengeliidae	2	3	ND	ND	2	0			
		Pterobranchia		3	3	ND	ND	3	0			
	Kinorhyncha			7		4	100	107	0	1 (Yoshihisa Shirayama: Kyoto University)	0	
		Kinorhyncha		7	3	4	100	107	0			
	Loricifera			1		1	10	11	0			
		Loricifera		1		1	10	11	0			
			Nanaloricida	1	3	1	10	11	0			
	Mollusca			8,658		334	1,180	9,838	11			
		Bivalvia		1,252		46	64	1,316	7	3 (Takashi Okutani: JAMSTEC, Akihiko Matsukuma: Kyushu University, et al.)	>10	Higo S, Callomon P, Goto Y（1999）Catalogue and bibliography of the marine shell-bearing Mollusca of Japan. Osaka: Elle Scientific Publications. 749 p.
			Arcoida	79	5	ND	9	88	0			
			Limoida	34	5	ND	3	37	0			
			Myoida	70	5	ND	2	72	0			
			Mytiloida	96	5	ND	5	101	4			
			Nuculoida	79	5	ND	2	81	0			
			Ostreoida	25	5	ND	0	25	0			
			Pectinoida	114	5	ND	2	116	0			
			Pholadomyoida	96	5	ND	0	96	0			
			Pterioida	44	5	ND	3	47	0			
			Solemyoida	9	5	ND	1	10	0			
			Veneroida	606	5	ND	37	643	3			
		Caudofoveata		3		ND	25	28	0	1 (Hiroshi Saito: NMNS)		http://research2.kahaku.go.jp/ujssb/search
			Chaetodermatida	3	3	ND	25	28	0			

		Cephalopoda		204		ND	5	209	0	3 (Takashi Okutani: JAMSTEC, Tsunemi Kubodera: NMNS, et al.)		土屋光太郎・阿部秀樹・山本典暎（2002）イカ・タコガイドブック. 東京：TBS ブリタニカ. 139 p.
			Nautiloida	1	5	ND	0	1	0			
			Octopoda	61	5	ND	2	63	0			
			Sepioida	22	5	ND	1	23	0			
			Sepiolida	15	5	ND	0	15	0			
			Teuthoida	104	5	ND	2	106	0			
			Vampyromorpha	1	5	ND	0	1	0			
		Gastropoda		7,013		286	965	7,978	4	6 (Takashi Okutani: JAMSTEC, Takenori Sasaki: University of Tokyo, et al.)	>10	Higo S, Callomon P, Goto Y（1999）Catalogue and bibliography of the marine shell-bearing Mollusca of Japan. Osaka: Elle Scientific Publications. 749 p.
			Acochlidiacea	0	5	ND	2	2	0			
			Aplysiomorpha	21	5	ND	5	26	0			
			Basommatophora	13	5	ND	0	13	0			
			Cephalaspidea	279	4	ND	72	351	0			
			Cocculiniformia	14	5	ND	3	17	0			
			Discopoda	973	5	ND	182	1,155	3			
			Eupulmonata	32	5	ND	0	32	0			
			Gymnosomata	9	5	ND	0	9	0			
			Heteropoda	23	5	ND	0	23	0			
			Heterostropha	583	5	ND	137	720	0			
			Neogastropoda	2,034	5	ND	140	2,174	0			
			Neomphalina	1	5	ND	0	1	0			
			Neritimorpha	97	5	ND	12	109	0			
			Nudibranchia	435	4	ND	200	635	1			
			Patellogastropoda	52	5	ND	1	53	0			
			Pleurobranchomorpha	16	4	ND	5	21	0			
			Ptenoglossa	465	5	ND	95	560	0			
			Runcinacea	2	5	ND	0	2	0			
			Saccoglossa	69	5	ND	10	79	0			
			Sorbeoconcha	1,375	5	ND	1	1,376	0			
			Systellommatophora	6	3	ND	5	11	0			
			Thecosomata	33	5	ND	0	33	0			
			Vetigastropoda	481	5	ND	95	576	0			
		Polyplacophora		129		ND	48	177	0	1 (Hiroshi Saito: NMNS)	>2	瀧巖（1965）多板綱. In: 岡田要・内田清之助・内田亨， 編. 新日本動物図鑑（中）. 東京：北隆館 803 p.
			Neoloricata	129	4	ND	48	177	0			

		Scaphopoda		47		2	0	47	0	1 (Takashi Okutani: JAMSTEC)		http://research2.kahaku.go.jp/ujssb/search
			Dentaliida	25	5	0	0	25	0			
			Gadiliida	22	5	2	0	22	0			
		Solenogastres		10		ND	73	83	0	1 (Hiroshi Saito: NMNS)	0	
			Cavibelonia	9	3	ND	60	69	0			
			Neomeniamorpha	1	3	ND	7	8	0			
			Pholidoskepia	0	1	ND	5	5	0			
			Sterrofustia	0	1	ND	1	1	0			
	Myxozoa			62		6	ND	62	2	1 (Hiroshi Yokoyama:University of Tokyo)	6	Eiras JC（2006）Synopsis of the species of *Ceratomyxa* Thelohan, 1892 （Myxozoa: Myxosporea: Ceratomyxidae）. Syst Parasit 65: 49-71.
		Myxosporea		62		6	ND	62	2			
			Bivalvulida	47	4	6	ND	47	1			
			Multivalvulida	15	4	ND	ND	15	1			
	Nematoda			70		35	115,010	115,080	0	1 (Yoshihisa Shirayama: Kyoto University)	>1	Yoshikawa N（2002）Brief review of Japanese researches on free living marine nematodes. In: Nakasone K, editor. Progress in Nematology, Tsukuba: Jpn Soc Nemato. pp. 64-70.
		Adenophorea		69		35	115,000	115,069	0			
			Araeolaimida	2	3	1	10,000	10,002	0			
			Chromadorida	29	4	16	30,000	30,029	0			
			Desmodora	9	3	6	20,000	20,009	0			
			Desmoscolecida	0	1	0	5,000	5,000	0			
			Enoplida	18	4	9	20,000	20,018	0			
			Monhysterida	11	4	3	30,000	30,011	0			
		Secernentea		1		0	10	11	0			
			Rhabditida	1	3	0	10	11	0			
	Nematomorpha			1		1	100	101	0		0	
		Nematomorpha		1		1	100	101	0			
			Nectonematoida	1	3	1	100	101	0			
	Nemertea			120		ND	58	178	0	1 (Hiroshi Kajihara: Hokkaido University)	ND	http://research2.kahaku.go.jp/ujssb/search
		Hoplonemertea		57		ND	36	93	0			
			Amphiporidae	14	4	ND	10	24	0			
			Carcinonemertidae	1	5	ND	0	1	0			
			Cratenemertidae	3	3	ND	3	6	0			
			Emplectonematidae	10	4	ND	3	13	0			
			Malacobdellidae	1	5	ND	0	1	0			
			Ototyphlonemertidae	3	3	ND	3	6	0			

			Pelagica	2	5	ND	0	2	0			
			Reptantia	3	3	ND	4	7	0			
			Poseidonemertidae	1	3	ND	4	5	0			
			Prosorhochmidae	2	3	ND	3	5	0			
			Tetrastemmatidae	17	4	ND	6	23	0			
		Palaeonemertea		11		ND	4	15	0			
			Callineridae	1	4	ND	ND	ND	0			
			Cephalotrichidae	3	3	ND	2	5	0			
			Tubulanidae	7	4	ND	2	9	0			
		Pilidiophora		52		ND	18	70	0			
			Gorgonorhynchidae	0	1	ND	1	1	0			
			Hubrechtiidae	3	4	ND	1	4	0			
			Lineidae	42	4	ND	15	57	0			
			Valenciniidae	7	5	ND	1	8	0			
	Orthonecta			1	3	ND	3	4	0	1 (Hidetaka Furuya: Osaka University)	ND	
	Phoronida			2		ND	ND	2	0	2 (Hiroshi Kajihara: Hokkaido University, Shunsuke Mawatari: Hokkaido University)	ND	http://research2.kahaku.go.jp/ujssb/search
		Phoranidea		2		ND	ND	2	0			
			Phoronidae	2	3	ND	ND	2	0			
	Placozoa			1	3	ND	ND	1	0	1 (Hiroshi Kajihara: Hokkaido University)	ND	
	Platyhelminthes			188		ND	350	538	0	2 (Ken-ichi Tajika: Nihon University, Morio Hagiya: Atsugi-nishi High School)	1	
		Catenulida		0		ND	50	50	0			
			Catenulida	0	1	ND	50	50	0			
		Eulecithophora		37		ND	100	137	0			
			Dalyellioida	5	3	ND	ND	5	0			
			Tricladida	6	3	ND	ND	6	0			
			Proseriata	26	4	ND	100	126	0			
			Urastomatida	ND	1	ND	ND	ND	0			
		Lecithoepitheliata		0		ND	50	50	0			
			Prorhynchidae	0	1	ND	50	50	0			
		Macrostomorpha		1		ND	50	51	0			
			Macrostomida	1	3	ND	50	51	0			
		Polycladida		150		ND	100	250	0			
			Polycladida	150	5	ND	100	250	0			
	Porifera			745		ND	540	1,285	0	1 (Yuji Ise: University of Tokyo)	2	
		Calcarea		130		ND	20	150	0			Ogawa K（2006）Porifera of the Sagami

												Sea. Mem Nat Sci Mus Tokyo 40: 23–56.
			Baerida	2	3	ND	1	3	0			
			Clathrinida	17	4	ND	8	25	0			
			Leucosolenida	110	5	ND	10	120	0			
			Lithonida	1	3	ND	1	2	0			
		Demospongiae		537		ND	500	1,037	0		3	http://research2.kahaku.go.jp/ujssb/search
			Agelasida	2	3	ND	10	12	0			Hoshino T（1985）Description of two new species in the genus *Agelas*（Demospongia）from Zamami Island, the Ryukyus, Japan. Pro Jap Spci Syst Zool 30: 1–10.
			Astrophorida	83	4	ND	30	113	0			谷田専治（1989）相模湾産尋常海綿類. 東京：丸善. 197 p.
			Chondrosida	3	3	ND	5	8	0			
			Dendroceratida	3	3	ND	15	18	0			
			Dictyoceratida	20	4	ND	15	35	0			
			Hadromerida	63	4	ND	100	163	0			
			Halichondrida	65	4	ND	80	145	0			
			Halisarcida	0	1	ND	2	2	0			
			Haplosclerida	105	5	ND	80	185	0			
			Homoscleromorpha	4	3	ND	3	7	0			
			"Lithistida"	11	4	ND	30	41	0			
			Poecilosclerida	158	5	ND	100	258	0			
			Spirophorida	20	4	ND	20	40	0			
			Verongida	0	1	ND	10	10	0			
		Hexactinellida		78		ND	20	98	0		14	Hoshino T（1985）Description of two new species in the genus *Agelas*（Demospongia）from Zamami Island, the Ryukyus, Japan. Pro Jap Spci Syst Zool 30: 1–10.
			Amphidiscosida	16	4	ND	6	22	0			
			Hexactinosida	21	4	ND	7	28	0			
			Lyssacinosida	41	4	ND	7	48	0			
	Priapulida			2		0	20	22	0	1 (Yoshihisa Shirayama: Kyoto University)	0	http://research2.kahaku.go.jp/ujssb/search
		Priapula		2		0	20	22	0			
			Maccabeidae	0	1	0	5	5	0			
			Priapulidae	2	3	0	10	12	0			
			Tubiluchidae	0	1	0	5	5	0			
	Rotifera			397		ND	1	398	0	1 (Minoru Sudzuki: Nihon University)	0	
		Archeorotatoria		83		ND	0	83	0			
			Bdelloidea	83	5	ND	0	83	0			

		Eurotatoria		313		ND	0	313	0			
			Antrorsiramida	15	5	ND	0	15	0			
			Protoramida	30	5	ND	0	30	0			
			Salitiramida	13	5	ND	0	13	0			
			Septiramida	48	5	ND	0	48	0			
			Transversiramida	207	5	ND	1	208	0			
		Hemirotatoria		1		ND	0	1	0			
			Paedotrochida	1	3	ND	0	1	0			
	Sipuncula			47		ND	ND	47	0	1 (Teruaki Nishikawa: Toho University)	>2	
		Sipunculidea		28		ND	ND	28	0			
			Golfingiiformes	23	4	ND	ND	23	0			
			Sipunculiformes	5	3	ND	ND	5	0			
		Phascolosomatidea		19		ND	ND	19	0			
			Aspidosiphoniformes	10	4	ND	ND	10	0			
			Phascolosomatiformes	9	3	ND	ND	9	0			
	Tardigrade			14		ND	9	23	0	3	ND	http://homepage3.nifty.com/cxj11255/jtard/index.html
		Heterotardigrada		13		ND	9	22	0			
			Arthrotardigrada	13	4	ND	9	22	0			
		Eutardigrada		1		ND	ND	1	0			
			Parachela	1	3	ND	ND	1	0			
			Total	33,629		1,872	121,913	155,542	39			

[1] 略語説明は Table 2 参照.

Table S4. 各門における日本近海の出現種数（NDS），今後出現が記録される種数（NUS），推定種数（ENS）.

Domain	Phylum/Division	NDS	NUS	ENS
Eukarya	Mollusca	8,658	1,180	9,838
Eukarya	Arthropoda	6,393	1,677	8,070
Eukarya	Chordata	4,330	326	4,656
Eukarya	Granuloreticulosa	2,321	490	2,811
Eukarya	Cnidaria	1,876	364	2,240
Eukarya	Heterokontophyta	1,207	ND	1,207
Eukarya	Annelida	1,076	124	1,200
Eukarya	Echinodermata	1,052	ND	1,052
Eukarya	Rhodophyta	898	ND	898
Eukarya	Porifera	745	540	1,285
Eukarya	Ciliophora	530	ND	530
Eukarya	Dinomastigota	470	ND	470
Eukarya	Radiozoa	405	100	505
Eukarya	Rotifera	397	1	398
Bacteria	Proteobacteria	395	ND	395
Eukarya	Haptophyta	304	ND	304
Eukarya	Ectoprocta	300	900	1,200
Eukarya	Ascomycota	294	ND	294
Eukarya	Chlorophyta	248	ND	248
Eukarya	Platyhelminthes	188	350	538
Bacteria	Firmicutes	172	ND	172
Eukarya	Cercozoa	143	4	147
Bacteria	Bacteroides	125	ND	125
Bacteria	Actinobacteria	123	ND	123
Eukarya	Dicyemida	120	30	150
Eukarya	Nemertea	120	58	178
Eukarya	Brachiopoda	73	27	100
Eukarya	Nematoda	70	115,010	115,080
Eukarya	Basidiomycota	65	ND	65
Eukarya	Myxozoa	62	ND	62
Eukarya	Choanozoa	54	ND	54
Eukarya	Sipuncula	47	ND	47
Eukarya	Magnoliopsida	44	ND	44
Eukarya	Ctenophora	41	13	54
Eukarya	Entoprocta	41	210	251
Eukarya	Labyrinthulomycota	40	ND	40
Eukarya	Chaetognatha	36	6	42
Eukarya	Echiura	21	ND	21
Eukarya	Euglenophyta	14	ND	14
Eukarya	Tardigrade	14	9	23
Eukarya	Gastrotricha	12	100	112
Bacteria	Cyanobacteria	11	1	12
Eukarya	Hemichordata	11	10	21

Eukarya	Acoelomorpha	10	50	60
Eukarya	Cryptophyta	8	ND	8
Eukarya	Zygomycota	8	ND	8
Archaea	Euryarchaeota	7	ND	7
Eukarya	Kinorhyncha	7	100	107
Eukarya	Metamonada	6	ND	6
Eukarya	Gnathostomulida	5	100	105
Bacteria	Thermotogae	4	ND	4
Bacteria	Verrucomicrobia	4	ND	4
Eukarya	Glaucophyta	4	ND	4
Bacteria	Acidobacteria	3	ND	3
Archaea	Crenarchaeota	2	ND	2
Bacteria	Aquificae	2	ND	2
Bacteria	Nitrospirae	2	ND	2
Eukarya	Phoronida	2	ND	2
Eukarya	Priapulida	2	20	22
Bacteria	Deferribacteres	1	ND	1
Bacteria	Deinococci	1	ND	1
Eukarya	Mesostigmatophyceae (Class)	1	ND	1
Eukarya	Loricifera	1	10	11
Eukarya	Nematomorpha	1	100	101
Eukarya	Orthonecta	1	3	4
Eukarya	Placozoa	1	ND	1
Archaea	Thaumarchaeota	ND	ND	ND
Eukarya	Apicomplexa	ND	ND	ND
Eukarya	Percolozoa	ND	ND	ND
Eukarya	Amoebozoa	ND	ND	ND
Eukarya	Heliozoa	ND	ND	ND
Eukarya	Blastocladiomycota	ND	ND	ND
Eukarya	Chytridiomycota	ND	ND	ND
Eukarya	Glomeromycota	ND	ND	ND
Eukarya	Microsporidia	ND	ND	ND
Eukarya	Oomycota	ND	ND	ND
Eukarya	Opalozoa	ND	ND	ND
Eukarya	Acanthocephala	ND	ND	ND
Eukarya	Cycliophora	ND	ND	ND
	Total	33,629	121,913	155,542

ND=No Data

Table S5. 日本近海固有種数 (NES) と出現種数（NDS）に対する固有種数の割合（PES）.

分類群			NES	NDS[1]	PES (%)[2]
門	綱	目			
Granuloreticulosa	Foraminifera		383	2,321	17
Chordata	Actinopterygii		358	3,770	9
Mollusca	Gastropoda		286	7,013	4
Arthropoda	Malacostraca	Mysida	117	226	52
Cnidaria	Anthozoa	Gorgonacea	115	241	48
Arthropoda	Pycnogonida		60	153	39
Cnidaria	Hydrozoa	Athecata	53	130	41
Cnidaria	Anthozoa	Alcyonacea	50	223	22
Arthropoda	Malacostraca	Euphausiacea	49	86	57
Cnidaria	Hydrozoa	Thecata	46	286	16
Mollusca	Bivalvia		46	1,252	4
Dicyemida			40	120	33
Nematoda			35	70	50
Entoprocta			33	41	80
Arthropoda	Malacostraca	Decapoda	28	2,501	1
Cnidaria	Anthozoa	Actiniaria	26	174	15
Cnidaria	Anthozoa	Pennatulacea	24	71	34
Arthropoda	Maxillopoda	Calanoida	22	743	3
Ctenophora			15	41	37
Cnidaria	Anthozoa	Scleractinia	10	530	2
Cnidaria	Anthozoa	Stolonifera	8	18	44
Myxozoa	Myxosporea	Bivalvulida	6	47	13
Arthropoda	Maxillopoda	Monstrilloida	5	12	42
Chaetognatha			5	36	14
Cnidaria	Anthozoa	Telestacea	5	8	63
Chordata	Chondrichthyes		5	11	45
Cnidaria	Hydrozoa	Siphonophora	4	54	7
Cnidaria	Scyphozoa		4	37	11
Kinorhyncha			4	7	57
Arthropoda	Maxillopoda	Laurida	3	10	30
Arthropoda	Maxillopoda	Tantulocarida	3	4	75
Cnidaria	Cubozoa	Cubomedusae	3	4	75
Gastrotricha			3	12	25
Annelida	Polychaeta	Myzostomida	2	30	7
Annelida	Polychaeta	Spionida	2	89	2
Arthropoda	Maxillopoda	Platycopioida	2	2	100
Cnidaria	Hydrozoa	Limnomedusae	2	8	25
Mollusca	Scaphopoda		2	47	4
Annelida	Polychaeta	Phyllodocida	1	466	0
Annelida	Polychaeta	Polygordiida	1	2	50
Arthropoda	Maxillopoda	Misophrioida	1	3	33
Arthropoda	Maxillopoda	Dendrogastrida	1	3	33
Arthropoda	Malacostraca	Lophogastrida	1	14	7
Chordata	Myxini		1	5	20
Loricifera			1	1	100
Nematomorpha			1	1	100

Arthropoda	Maxillopoda	Mormonilloida	0	2	0
Chordata	Appendicularia		0	31	0
Chordata	Cephalaspidomorphi		0	4	0
Chordata	Mammalia		0	46	0
Chordata	Thaliacea		0	40	0
Cnidaria	Anthozoa	Helioporacea	0	1	0
Cnidaria	Hydrozoa	Laingiomedusa	0	1	0
Cnidaria	Hydrozoa	Narcomedusae	0	9	0
Cnidaria	Hydrozoa	Trachymedusae	0	16	0
Gnathostomulida			0	5	0
Haptophyta			0	304	0
Priapulida			0	2	0
Others			ND	12,247	ND
Total			1,872	33,629	

[1] 出現種数
[2] それぞれの分類群における出現種数に対する固有種数の割合

Table S6. 日本近海への外来種と移入メカニズムおよび原産域の推定

分類群		推定される移入メカニズム	推定される原産域	引用文献
門	種			
Annelida	*Boccardia proboscidea*	Ballast water, Fisheries	Unknown	
Annelida	*Ficopomatus enigmaticus*	Hull fouling	Unknown	西栄二郎・加藤哲哉（2004）環形動物多毛類の移入と移出の現状.日本ベントス学会誌　59: 83-95.,　Link H, Nishi E, K. Tanaka, R. Bastida-zavala, E. K. Kupriyanova et al.（2009）*Hydroides dianthus*　（Polychaeta: Serpulidae）, an alien species introduced into Tokyo Bay, Japan. Mar Biodiv Rec: doi:10.1017/S1755267209000931
Annelida	*Harmothoe imbricata*	Unknown	Unknown	Link H, Nishi E, K. Tanaka, R. Bastida-zavala, E. K. Kupriyanova et al.（2009）*Hydroides dianthus*　（Polychaeta: Serpulidae）, an alien species introduced into Tokyo Bay, Japan. Mar Biodiv Rec: doi:10.1017/S1755267209000931
Annelida	*Hydroides elegans*	Hull fouling	Unknown	西栄二郎・加藤哲哉（2004）環形動物多毛類の移入と移出の現状.日本ベントス学会誌　59: 83-95.,　Link H, Nishi E, K. Tanaka, R. Bastida-zavala, E. K. Kupriyanova et al.（2009）*Hydroides dianthus*　（Polychaeta: Serpulidae）, an alien species introduced into Tokyo Bay, Japan. Mar Biodiv Rec: doi:10.1017/S1755267209000931
Annelida	*Hydroides dianthus*	Unknown	Unknown	西栄二郎・加藤哲哉（2004）環形動物多毛類の移入と移出の現状.日本ベントス学会誌　59: 83-95.,　Link H, Nishi E, K. Tanaka, R. Bastida-zavala, E. K. Kupriyanova et al.（2009）*Hydroides dianthus*　（Polychaeta: Serpulidae）, an alien species introduced into Tokyo Bay, Japan. Mar Biodiv Rec: doi:10.1017/S1755267209000931
Annelida	*Neanthes succenea*	Unknown	Unknown	Link H, Nishi E, K. Tanaka, R. Bastida-zavala, E. K. Kupriyanova et al.（2009）*Hydroides dianthus*　（Polychaeta: Serpulidae）, an alien species introduced into Tokyo Bay, Japan. Mar Biodiv Rec: doi:10.1017/S1755267209000931
Annelida	*Perinereis aibuhitensis*	Unknown	Unknown	Link H, Nishi E, K. Tanaka, R. Bastida-zavala, E. K. Kupriyanova et

				al.（2009）*Hydroides dianthus* （Polychaeta: Serpulidae）, an alien species introduced into Tokyo Bay, Japan. Mar Biodiv Rec: doi:10.1017/S1755267209000931
Annelida	Phyllodocida sp.	Unknown	Unknown	Link H, Nishi E, K. Tanaka, R. Bastida-zavala, E. K. Kupriyanova et al.（2009）*Hydroides dianthus* （Polychaeta: Serpulidae）, an alien species introduced into Tokyo Bay, Japan. Mar Biodiv Rec: doi:10.1017/S1755267209000931
Annelida	*Polydora cornuta*	Ballast water, Fisheries	Unknown	
Annelida	*Polydora* sp.	Ballast water, Fisheries	Unknown	
Arthropoda	*Balanus glandula*	Hull fouling	North-east Pacific	岩崎敬二・木村妙子・木下今日子・山口寿之・西川輝昭ほか（2004）日本における海産生物の人為的移入と分散：日本ベントス学会自然保全委員会によるアンケート調査の結果から.日本ベントス学会誌　59: 22-44., 大谷道夫（2004）日本の海洋移入生物とその移入過程について.日本ベントス学会誌　59: 45-57.
Arthropoda	*Balanus improvisus*	Hull fouling	North-east Atlantic	岩崎敬二・木村妙子・木下今日子・山口寿之・西川輝昭ほか（2004）日本における海産生物の人為的移入と分散：日本ベントス学会自然保全委員会によるアンケート調査の結果から.日本ベントス学会誌　59: 22-44., 大谷道夫（2004）日本の海洋移入生物とその移入過程について.日本ベントス学会誌　59: 45-57.
Arthropoda	*Balanus eburneus*	Hull fouling	West Atlantic	岩崎敬二・木村妙子・木下今日子・山口寿之・西川輝昭ほか（2004）日本における海産生物の人為的移入と分散：日本ベントス学会自然保全委員会によるアンケート調査の結果から.日本ベントス学会誌　59: 22-44., 大谷道夫（2004）日本の海洋移入生物とその移入過程について.日本ベントス学会誌　59: 45-57.
Arthropoda	*Balanus amphitrite*	Ballast water, Hull fouling	Unknown	岩崎敬二・木村妙子・木下今日子・山口寿之・西川輝昭ほか（2004）日本における海産生物の人為的移入と分散：日本ベントス学会自然保全委員会によるアンケート調査の結果から.日本ベントス学会誌　59: 22-44., 大谷道夫（2004）日本の海洋移入生物とその移入過程について.日本ベントス学会誌　59: 45-57.
Arthropoda	*Balanus variegatus cirratus*	Unknown	Unknown	岩崎敬二・木村妙子・木下今日子・山口寿之・西川輝昭ほか（2004）日本における海産生物の人為的移入と分散：日本ベントス学会自然保全委員会によるアンケート調査の結果から.日本ベントス学会誌　59: 22-44., 大谷道夫（2004）日本の海洋移入生物とその移入過程について.日本ベントス学会誌　59: 45-57.
Arthropoda	*Balanus venustus*	Unknown	Unknown	岩崎敬二・木村妙子・木下今日子・山口寿之・西川輝昭ほか（2004）日本における海産生物の人為的移入と分散：日本ベントス学会自然保全委員会によるアンケート調査の結果から.日本

Arthropoda	*Balanus venustus*	Unknown	Unknown	岩崎敬二・木村妙子・木下今日子・山口寿之・西川輝昭ほか（2004）日本における海産生物の人為的移入と分散：日本ベントス学会自然保全委員会によるアンケート調査の結果から.日本ベントス学会誌　59: 22-44., 大谷道夫（2004）日本の海洋移入生物とその移入過程について.日本ベントス学会誌　59: 45-57.
Arthropoda	*Callinectes sapidus*	Hull fouling	Atlantic, Caribbean Sea	岩崎敬二・木村妙子・木下今日子・山口寿之・西川輝昭ほか（2004）日本における海産生物の人為的移入と分散：日本ベントス学会自然保全委員会によるアンケート調査の結果から.日本ベントス学会誌　59: 22-44., 大谷道夫（2004）日本の海洋移入生物とその移入過程について.日本ベントス学会誌　59: 45-57.
Arthropoda	*Carcinus aestuarii*	Hull fouling	Mediterranean, Black Sea	岩崎敬二・木村妙子・木下今日子・山口寿之・西川輝昭ほか（2004）日本における海産生物の人為的移入と分散：日本ベントス学会自然保全委員会によるアンケート調査の結果から.日本ベントス学会誌　59: 22-44., 大谷道夫（2004）日本の海洋移入生物とその移入過程について.日本ベントス学会誌　59: 45-57.
Arthropoda	*Pyromaia tuberculata*	Hull fouling	East Pacific	岩崎敬二・木村妙子・木下今日子・山口寿之・西川輝昭ほか（2004）日本における海産生物の人為的移入と分散：日本ベントス学会自然保全委員会によるアンケート調査の結果から.日本ベントス学会誌　59: 22-44., 大谷道夫（2004）日本の海洋移入生物とその移入過程について.日本ベントス学会誌　59: 45-57.
Arthropoda	Siphonostomatoida sp.	Unknown	Unknown	岩崎敬二・木村妙子・木下今日子・山口寿之・西川輝昭ほか（2004）日本における海産生物の人為的移入と分散：日本ベントス学会自然保全委員会によるアンケート調査の結果から.日本ベントス学会誌　59: 22-44., 大谷道夫（2004）日本の海洋移入生物とその移入過程について.日本ベントス学会誌　59: 45-57.
Chlorophyta	*Caulerpa taxifolia*	Accidental release?	Indian Ocean, Caribbean Sea, Pacific	岩崎敬二・木村妙子・木下今日子・山口寿之・西川輝昭ほか（2004）日本における海産生物の人為的移入と分散：日本ベントス学会自然保全委員会によるアンケート調査の結果から.日本ベントス学会誌　59: 22-44., 大谷道夫（2004）日本の海洋移入生物とその移入過程について.日本ベントス学会誌　59: 45-57.
Chordata	*Lateolabrax* sp.	Fisheries	China, Korea	
Chordata	*Molgula manhattensis*	Hull fouling	Unknown	
Chordata	*Polyandrocarpa zorritensis*	Hull fouling	Atlantic	
Cnidaria	*Nemopilema nomurai*	Unknown	Unknown	
Heterokontophyta	*Cutleria multifida*	Hull fouling	Indian Ocean	岩崎敬二・木村妙子・木下今日子・山口寿之・西川輝昭ほか（2004）日本における海産生物の人為的移入と分散：日本ベン

Mollusca	*Mercenaria mercenaria*	Unknown	West Atlantic	岩崎敬二・木村妙子・木下今日子・山口寿之・西川輝昭ほか（2004）日本における海産生物の人為的移入と分散：日本ベントス学会自然保全委員会によるアンケート調査の結果から.日本ベントス学会誌　59: 22-44., 大谷道夫（2004）日本の海洋移入生物とその移入過程について.日本ベントス学会誌　59: 45-57.
Mollusca	*Meretrix petechialis*	Fisheries	China, Korea	
Mollusca	*Mytilopsis sallei*	Hull fouling, Cargo fouling	Caribbean Sea	岩崎敬二・木村妙子・木下今日子・山口寿之・西川輝昭ほか（2004）日本における海産生物の人為的移入と分散：日本ベントス学会自然保全委員会によるアンケート調査の結果から.日本ベントス学会誌　59: 22-44., 大谷道夫（2004）日本の海洋移入生物とその移入過程について.日本ベントス学会誌　59: 45-57.
Mollusca	*Mytilus galloprovincialis*	Hull fouling	Mediterranean, East Atlantic	岩崎敬二・木村妙子・木下今日子・山口寿之・西川輝昭ほか（2004）日本における海産生物の人為的移入と分散：日本ベントス学会自然保全委員会によるアンケート調査の結果から.日本ベントス学会誌　59: 22-44., 大谷道夫（2004）日本の海洋移入生物とその移入過程について.日本ベントス学会誌　59: 45-57.
Mollusca	*Perna viridis*	Hull fouling	Indian Ocean, South-east Asia	岩崎敬二・木村妙子・木下今日子・山口寿之・西川輝昭ほか（2004）日本における海産生物の人為的移入と分散：日本ベントス学会自然保全委員会によるアンケート調査の結果から.日本ベントス学会誌　59: 22-44., 大谷道夫（2004）日本の海洋移入生物とその移入過程について.日本ベントス学会誌　59: 45-57.
Mollusca	*Ptericola* sp. cf. *lithophaga*	Unknown	Unknown	岩崎敬二・木村妙子・木下今日子・山口寿之・西川輝昭ほか（2004）日本における海産生物の人為的移入と分散：日本ベントス学会自然保全委員会によるアンケート調査の結果から.日本ベントス学会誌　59: 22-44., 大谷道夫（2004）日本の海洋移入生物とその移入過程について.日本ベントス学会誌　59: 45-57.
Mollusca	*Xenostrobus securis*	Hull fouling	Oceania	岩崎敬二・木村妙子・木下今日子・山口寿之・西川輝昭ほか（2004）日本における海産生物の人為的移入と分散：日本ベントス学会自然保全委員会によるアンケート調査の結果から.日本ベントス学会誌　59: 22-44., 大谷道夫（2004）日本の海洋移入生物とその移入過程について.日本ベントス学会誌　59: 45-57.
Mollusca	*Crepidula onyx*	Hull fouling	East Pacific	岩崎敬二・木村妙子・木下今日子・山口寿之・西川輝昭ほか（2004）日本における海産生物の人為的移入と分散：日本ベントス学会自然保全委員会によるアンケート調査の結果から.日本ベントス学会誌　59: 22-44., 大谷道夫（2004）日本の海洋移入生物とその移入過程について.日本ベントス学会誌　59: 45-57.

Mollusca	*Cuthona perca*	Hull fouling	Brazil, Caribbean Sea	岩崎敬二・木村妙子・木下今日子・山口寿之・西川輝昭ほか（2004）日本における海産生物の人為的移入と分散：日本ベントス学会自然保全委員会によるアンケート調査の結果から.日本ベントス学会誌　59: 22-44., 大谷道夫（2004）日本の海洋移入生物とその移入過程について.日本ベントス学会誌　59: 45-57.
Mollusca	*Nassarius sinarus*	Fisheries	China	岩崎敬二・木村妙子・木下今日子・山口寿之・西川輝昭ほか（2004）日本における海産生物の人為的移入と分散：日本ベントス学会自然保全委員会によるアンケート調査の結果から.日本ベントス学会誌　59: 22-44., 大谷道夫（2004）日本の海洋移入生物とその移入過程について.日本ベントス学会誌　59: 45-57.
Mollusca	*Stenothyra* sp.	Fisheries	Unknown	岩崎敬二・木村妙子・木下今日子・山口寿之・西川輝昭ほか（2004）日本における海産生物の人為的移入と分散：日本ベントス学会自然保全委員会によるアンケート調査の結果から.日本ベントス学会誌　59: 22-44., 大谷道夫（2004）日本の海洋移入生物とその移入過程について.日本ベントス学会誌　59: 45-57.
Myxozoa	*Henneguya lateolabracis*	Fisheries	Unknown	小川和夫（2005）魚類寄生虫病.日本水産学会誌 71: 650-653.
Myxozoa	*Septemcapsula yasunagai*?	Fisheries	Unknown	小川和夫（2005）魚類寄生虫病.日本水産学会誌 71: 650-653.
